# Impact of COVID-19 on gastrointestinal disease in vaccinated and unvaccinated individuals: a population-based cohort study in England

**DOI:** 10.1136/bmjph-2025-004709

**Published:** 2026-10-20

**Authors:** Marwa Al Arab, Genevieve Cezard, Colin Crooks, Yinghui Wei, Elsie Horne, Tom M Palmer, Jose I Cuitun Coronado, Alex J Walker, Millie Green, Louis Fisher, Jon Massey, Simon Davy, Amir Mehrkar, Sebastian Bacon, Ben Goldacre, Nishi Chaturvedi, Angela Wood, Jonathan A C Sterne, Venexia Walker, Rachel Denholm

**Affiliations:** 1 Population Health Science, University of Bristol, Bristol, UK; 2 British Heart Foundation Cardiovascular Epidemiology Unit, Department of Public Health and Primary Care, University of Cambridge, Cambridge, UK; 3 Victor Phillip Dahdaleh Heart and Lung Research Institute, University of Cambridge, Cambridge, UK; 4 Translational Medical Sciences, University of Nottingham School of Medicine, Nottingham, UK; 5 Gastrointestinal and Liver Theme, National Institute for Health Research (NIHR) Nottingham Biomedical Research Centre (BRC), University of Nottingham, Nottingham, UK; 6 Faculty of Health and Life Sciences, University of Exeter, Exeter, UK; 7 Academic Unit of Ageing and Stroke Research, University of Leeds, Leeds, UK; 8 Bennett Institute for Applied Data Science, Nuffield Department of Primary Care Health Sciences, University of Oxford, Oxford, UK; 9 MRC Unit for Lifelong Health & Ageing, University College London, London, UK; 10 NIHR Bristol Biomedical Research Centre, Bristol, UK; 11 Department of Surgery, University of Pennsylvania Perelman School of Medicine, Philadelphia, Pennsylvania, USA

**Keywords:** COVID-19, Humans, SARS-CoV-2, Epidemiology, Vaccination

## Abstract

**Background:**

COVID-19 is associated with higher rates of gastrointestinal diseases, although the duration of this effect and the roles of vaccination and COVID-19 severity remain uncertain.

**Objective:**

To investigate the relationship between COVID-19 and gastrointestinal diseases by vaccination status in hospitalised and non-hospitalised patients.

**Design:**

With NHS England approval, OpenSAFELY-TPP was used to access linked data from 24 million English adults. We defined three cohorts: ‘prevaccination’ (January 2020–June 2021), ‘vaccinated’ and ‘unvaccinated’ (June–December 2021). We estimated adjusted HRs (aHRs) comparing the incidence of 10 gastrointestinal diseases, including upper and lower gastrointestinal bleeding, after vs before or without a COVID-19 diagnosis overall and by COVID-19 severity.

**Results:**

COVID-19 diagnosis was associated with elevated incidence of gastrointestinal diseases, particularly after hospitalised disease. In the prevaccination cohort (n=18 422 781), the aHRs for upper gastrointestinal bleeding after hospitalised COVID-19 were 20.1 (95% CI 18.4 to 22.1) in weeks 1–4 and 1.90 (1.63 to 2.22) in weeks 53–102. The corresponding aHRs after non-hospitalised COVID-19 were 1.92 (1.74 to 2.11) and 1.34 (1.23 to 1.45), respectively. Across time periods, aHRs for gastrointestinal diseases were lower in vaccinated (n=14 948 727) than in unvaccinated (n=3 479 043) individuals. These patterns were similar across gastrointestinal diseases.

**Conclusion:**

The incidence of gastrointestinal disease is elevated for up to 2 years among individuals hospitalised following a COVID-19 diagnosis, especially in the first few weeks after diagnosis. Increases in incidence are attenuated among vaccinated individuals. Monitoring for gastrointestinal diseases after severe COVID-19 and promotion of vaccination in vulnerable groups are key to reducing long-term burden.

**WHAT IS ALREADY KNOWN ON THIS TOPIC**

- Acute symptoms of COVID-19 include gastrointestinal problems, and there is existing evidence that those with a COVID-19 diagnosis are more likely to experience long-term gastrointestinal complications, especially if they were hospitalised.
- Previous studies were either limited in size, lacked representativeness of the population or had short follow-up (eg, during hospitalisation).
- The role of vaccination, COVID-19 severity or people’s characteristics (eg, age or sex) were not considered.

**WHAT THIS STUDY ADDS**

- Based on population-wide data on 18.4 million individuals, rates of 10 gastrointestinal diseases are elevated after COVID-19, compared with rates before or without COVID-19 diagnosis.
- The elevation in rates of gastrointestinal diseases after COVID-19 diagnosis was less marked in people who were vaccinated before COVID-19 diagnosis.
- The elevation in rates was markedly higher, and persisted for up to 2 years, among individuals hospitalised with COVID-19, compared with those who were not hospitalised.

**HOW THIS STUDY MIGHT AFFECT RESEARCH, PRACTICE OR POLICY**

- We show for the first time that the incidence of both acute and chronic gastrointestinal diseases remains elevated for up to 2 years after severe COVID-19 leading to hospitalisation.
- Our findings are consistent with lower post-COVID-19 gastrointestinal disease incidence among vaccinated individuals, supporting vaccination as an important public health intervention in reducing severe COVID-19 outcomes.
- Individuals who had severe COVID-19, regardless of vaccination status, should be prioritised for clinical monitoring for gastrointestinal diseases.

## Introduction

 COVID-19 can lead to long-term adverse outcomes that affect several body systems, including the gastrointestinal system.^1,2^ Potential mechanisms include direct infection of gastrointestinal epithelial cells via ACE2 receptors,^3^ immune dysregulation, persistent viral presence in the gut or microvascular injury.^4,5^ Previous studies have identified associations of COVID-19 with gastrointestinal diseases in both hospitalised^6–12^ and non-hospitalised individuals,^13–17^ for up to 2.6 years after infection.^18,19^ A recent meta-analysis estimated that up to 22% of patients with long covid experience gastrointestinal symptoms, including dyspepsia, abdominal pain, loss of appetite and irritable bowel syndrome (IBS).^20^ However, published studies are limited by single-centre data, short follow-up time or non-representative samples, and many focused only on symptoms or a narrow range of diseases.

COVID-19 vaccination substantially reduces the risk of severe COVID-19,^21^ but there is mixed evidence on its protective effect against postacute sequelae. Some studies found that vaccination reduced the risk of postacute sequelae of COVID-19 including neurological and gastrointestinal symptoms, such as abdominal pain, loss of appetite, constipation and diarrhoea.^22^ Other studies found no protection by COVID-19 vaccination against postacute sequelae, such as anxiety, sleep disorders and type 2 diabetes, particularly in older individuals.^23^ Evidence on the impact of vaccination on postacute gastrointestinal diseases following COVID-19 remains limited.

Using linked electronic health records (EHRs) from England, we compared rates of 10 acute and chronic gastrointestinal diseases after a COVID-19 diagnosis with rates before or without COVID-19 across three cohorts defined by vaccination status (prevaccination, vaccinated and unvaccinated). We also examined associations in subgroups defined by COVID-19 severity, age, sex, ethnicity and prior gastrointestinal history.

## Methods

### Study design and data sources

We analysed data available through the OpenSAFELY platform, which provides secure, privacy-protecting access to linked EHRs for 24 million people registered with English general practices (GPs) using the TPP SystmOne software—approximately 40% of the English population. Primary care data were linked via pseudonymised National Health Service (NHS) number to Secondary Uses Service (SUS) secondary care data, Office for National Statistics (ONS) Death Registry, Second Generation Surveillance System (SGSS) COVID-19 testing data and the Index of Multiple Deprivation (IMD). COVID-19 vaccination records (National Immunisation Management System) are available within TPP primary care data.

Gastrointestinal disease diagnoses and acute events included upper and lower gastrointestinal bleeding, acute pancreatitis, appendicitis, gastro-oesophageal reflux, dyspepsia, peptic ulcer, non-alcoholic steatohepatitis, gallstones and IBS. Symptoms were not included as outcomes due to inconsistent recording in primary care. Established codelists identified from OpenCodelists were used where available. New codelists were defined through structured searches followed by manual clinical review (online supplemental table 1 and 2).^24^ Date of outcome was defined as the earliest of a relevant SNOMED CT code recorded in primary care; the start of a secondary care episode with a corresponding International Classification of Diseases, 10th Revision (ICD-10) diagnosis code in any position or death with an ICD-10 code indicating the diagnosis as either a primary or underlying cause.

Date of COVID-19 diagnosis was defined as the earliest of positive SARS-CoV-2 PCR or antigen test recorded in SGSS; confirmed COVID-19 diagnosis recorded in primary care; start of an episode with a confirmed diagnosis in any position for a hospital admission record in SUS or death with COVID-19 listed as primary or underlying cause of death in the ONS registry. People with a hospital admission record in SUS that included a confirmed COVID-19 in the primary position and within 28 days of first COVID-19 diagnosis were defined as having had ‘hospitalised COVID-19’. All other COVID-19 diagnoses were classified as ‘non-hospitalised COVID-19’. COVID-19 hospitalisation was used as a proxy for disease severity, with hospitalised cases representing more severe disease and non-hospitalised cases representing less severe disease.

Covariates identified as potential confounders at baseline included age, sex, ethnicity, area-level socioeconomic deprivation (IMD quintiles), smoking status, obesity, alcohol consumption above the recommended limit, care home residence, healthcare worker and number of GP-patient interactions in 2019 and comorbidities. Comorbidities were selected with a clinician following a literature review, and included prior gastrointestinal symptoms and diseases, *Helicobacter pylori* infection, gastrointestinal surgical procedures and medication use (non-steroidal anti-inflammatory drugs, aspirin, anticoagulants and antidepressants), which are known to be associated with gastrointestinal outcomes. Binary indicators for these covariates were included (online supplemental table 1 and 2, online supplemental figure 2).

### Study population

We defined three cohorts based on vaccination status and predominant SARS-CoV-2 variant (online supplemental table 3, online supplemental figures 1 and 3). In the ‘prevaccination’ cohort, when Alpha variant was dominant, exposure was defined as a COVID-19 diagnosis recorded between 1 January 2020 (index date (time zero)) and the earliest of eligibility for COVID-19 vaccination, date of first vaccination or 18 June 2021 (when all adults became eligible for vaccination). Outcomes were ascertained from baseline until the earliest of outcome event date, date of death or 14 December 2021. In the ‘vaccinated’ and ‘unvaccinated’ cohorts, the follow-up period was defined between 1 June 2021 (start date), when the Delta variant was dominant (>50% of variance sequenced infections) and 14 December 2021, when Omicron became the dominant variant.^25^ In the ‘vaccinated’ cohort, index date was defined as the later of baseline or 2 weeks after a second COVID-19 vaccination and ended at the earliest of the outcome event date, date of death or 14 December 2021. In the ‘unvaccinated’ cohort, individuals were included if they had not received a COVID-19 vaccine within 12 weeks after becoming eligible. Index date was defined as the later of baseline or 12 weeks after vaccination eligibility and ended at the earliest of outcome event date, date of death, date of first vaccination or 14 December 2021.

In each cohort, individuals were eligible if they were alive with a known age (between 18 and 110 years), sex, deprivation and region at baseline, and were registered with a GP in England for at least 6 months before the study start date. Individuals were excluded if they had any record of COVID-19 diagnosis before baseline. In the vaccinated cohort, individuals were excluded if they received a vaccination before the start of the vaccine rollout on 8 December 2020 (indicating an error or participation in a randomised trial); their second dose was dated before their first dose vaccination (contradictory vaccine record); their second dose was <3 weeks after their first dose or they received mixed vaccine brands before this was permitted on 7 May 2021. In the unvaccinated cohort, individuals were excluded if they had any record of a COVID-19 vaccination before 1 June 2021. Vaccine eligibility was defined using the Joint Committee on Vaccination and Immunisation (JCVI) groupings. Individuals who could not be assigned to a JCVI group were excluded. We also excluded individuals with a history of Crohn’s disease, coeliac disease or cirrhosis, as we were interested in incident diagnosis and they are known to have a high baseline risk of gastrointestinal complications; and for the appendicitis analyses, those with prior diagnoses of appendicitis.

### Statistical analyses

Baseline demographics and clinical characteristics were summarised for each cohort. For each outcome, we calculated the number of events, person-years follow-up and incidence rates per 100 000 person-years before and after COVID-19, separately for individuals with hospitalised, non-hospitalised COVID-19 and no COVID-19 across each cohort.

For each outcome, we conducted time to first event analyses using Cox models with calendar timescale, with the cohort-specific baseline as the origin. We estimated HRs comparing follow-up after with follow-up before or without COVID-19. Follow-up after COVID-19 was split into the day of COVID-19 diagnosis (‘day 0’), the remainder of 1–4 weeks and 5–28 weeks after COVID-19 diagnosis for all cohorts, and additionally 29–52 and 53–102 weeks after COVID-19 diagnosis for the prevaccination cohort. We focused inferences on events after day 0, because the ordering of exposure and outcome events on day 0 cannot be determined.

For computational efficiency, we included all individuals with the outcome event or exposure and a 20% random sample of those without either. We used inverse probability weights to adjust for the sampling and used robust SEs to derive CIs to account for sampling design.

For each outcome across the three cohorts, we reported both age-adjusted and sex-adjusted and maximally adjusted (for all potential confounders) HRs. Age was modelled using restricted cubic splines unless otherwise specified. All models were stratified by region to account for variation in baseline hazard across regions. Subgroup analyses according to age, sex, ethnicity, pre-existing gastrointestinal diseases and prior gastrointestinal surgical procedures were conducted.

We calculated absolute excess risk 28 weeks after COVID-19 diagnosis, excluding events recorded on the day of COVID-19 diagnosis (‘day 0’) and weighted by the proportion of people in age strata in the prevaccination cohort. Additional details are provided in the online supplemental methods.

### Patient and public involvement and engagement

OpenSAFELY has involved patients and the public in various ways: we developed a public website that provides a detailed description of the platform in language suitable for a lay audience (https://opensafely.org); we have participated in two citizen juries exploring public trust in OpenSAFELY; we have co-developed an explainer video (https://www.opensafely.org/about/; we have patient representation who are experts by experience on our OpenSAFELY Oversight Board; we have partnered with Understanding Patient Data to produce lay explainers on the importance of large datasets for research; we have presented at various online public engagement events to key communities (eg, Healthcare Excellence Through Technology; Faculty of Clinical Informatics annual conference; NHS Assembly; Health Data Research UK symposium) and more. To ensure the patient voice is represented, we are working closely to decide on language choices with appropriate medical research charities (eg, Association of Medical Research Charities). We will share information and interpretation of our findings through press releases, social media channels and plain language summaries.

## Results

Outcomes are presented based on acuity, starting with acute events associated with hospital admissions (upper gastrointestinal bleeding, lower gastrointestinal bleeding, acute pancreatitis and appendicitis), followed by more chronic diseases (gastro-oesophageal reflux, dyspepsia, peptic ulcer, non-alcoholic steatohepatitis, gallstones and IBS). Outcomes with insufficient event counts to run models in all cohorts and by hospitalised COVID-19 status are included in the online supplemental material only, with findings briefly summarised in the text.

### Cohort characteristics

The prevaccination cohort included 18 422 781 people, of whom 1 000 437 (5.43%) received a COVID-19 diagnosis (table 1, online supplemental figure 4). The median (IQR) age was 49 (34–64) years. The cohort was 50.1% female and 79.6% white, 6.4% South Asian and 2.2% black ethnicities were recorded. Among 14 948 727 individuals in the vaccinated cohort, 858 903 (5.75%) were diagnosed with COVID-19, compared with 149 649 (4.31%) of the 3 479 043 individuals in the unvaccinated cohort. Individuals in the vaccinated cohort were more likely to be older, female, of white ethnicity, people who did not smoke and from less deprived areas than those in the unvaccinated cohort (table 1).^25^

**Table 1:** Patient characteristics in the prevaccination, vaccinated and unvaccinated cohorts

Characteristic		Prevaccination cohort (1 January 2020 to 14 December 2021)		Vaccinated cohort (1 June to 14 December 2021)		Unvaccinated cohort (1 June to 14 December 2021)	
		N (%)	COVID-19 diagnoses	N (%)	COVID-19 diagnoses	N (%)	COVID-19 diagnoses
All		18 422 781	1 000 437	14 948 727	858 903	3 479 043	149 649
Sex	Female	9 232 485 (50.1)	536 361 (53.6)	7 795 041 (52.1)	476 889 (55.5)	1 483 269 (42.6)	79 089 (52.8)
	Male	9 190 299 (49.9)	464 073 (46.4)	7 153 683 (47.9)	382 011 (44.5)	1 995 771 (57.4)	70 557 (47.1)
Age (years)	18–29	3 255 969 (17.7)	243 117 (24.3)	2 035 587 (13.6)	93 309 (10.9)	1 106 505 (31.8)	40 125 (26.8)
	30–39	3 198 483 (17.4)	202 173 (20.2)	2 152 167 (14.4)	147 555 (17.2)	1 036 929 (29.8)	50 655 (33.8)
	40–49	3 026 439 (16.4)	188 055 (18.8)	2 380 899 (15.9)	234 345 (27.3)	638 175 (18.3)	33 441 (22.3)
	50–59	3 217 857 (17.5)	181 695 (18.2)	2 875 101 (19.2)	202 167 (23.5)	386 961 (11.1)	16 737 (11.2)
	60–69	2 509 407 (13.6)	91 107 (9.1)	2 382 951 (15.9)	104 619 (12.2)	189 645 (5.5)	5619 (3.8)
	70–79	2 030 103 (11)	50 349 (5)	1 997 001 (13.4)	53 793 (6.3)	81 357 (2.3)	1935 (1.3)
	80–89	974 907 (5.3)	31 353 (3.1)	927 939 (6.2)	18 561 (2.2)	30 807 (0.9)	909 (0.6)
	90+	209 607 (1.1)	12 591 (1.3)	197 073 (1.3)	4557 (0.5)	8667 (0.2)	231 (0.2)
Ethnicity	White	14 661 387 (79.6)	763 557 (76.3)	12 434 337 (83.2)	741 459 (86.3)	2 188 215 (62.9)	115 299 (77)
	Mixed	215 679 (1.2)	14 145 (1.4)	141 975 (0.9)	7473 (0.9)	88 257 (2.5)	3867 (2.6)
	South Asian	1 181 109 (6.4)	117 141 (11.7)	899 217 (6)	41 391 (4.8)	354 735 (10.2)	9603 (6.4)
	Black	398 733 (2.2)	26 973 (2.7)	240 585 (1.6)	9573 (1.1)	186 399 (5.4)	7677 (5.1)
	Other	421 011 (2.3)	20 169 (2)	257 331 (1.7)	10 635 (1.2)	198 021 (5.7)	4053 (2.7)
	Missing	1 544 859 (8.4)	58 449 (5.8)	975 285 (6.5)	48 363 (5.6)	463 413 (13.3)	9141 (6.1)
IMD quintile	1	3 532 713 (19.2)	244 479 (24.4)	2 478 633 (16.6)	134 967 (15.7)	1 039 347 (29.9)	45 843 (30.6)
	2	3 647 499 (19.8)	216 567 (21.6)	2 812 839 (18.8)	158 157 (18.4)	831 717 (23.9)	35 433 (23.7)
	3	3 993 903 (21.7)	200 643 (20.1)	3 297 957 (22.1)	185 121 (21.6)	695 805 (20)	29 601 (19.8)
	4	3 762 423 (20.4)	180 867 (18.1)	3 238 347 (21.7)	189 741 (22.1)	531 519 (15.3)	22 461 (15)
	5	3 486 249 (18.9)	157 875 (15.8)	3 120 945 (20.9)	190 911 (22.2)	380 655 (10.9)	16 305 (10.9)
Smoking status	Missing	757 743 (4.1)	40 533 (4.1)	488 355 (3.3)	16 299 (1.9)	397 317 (11.4)	8421 (5.6)
	People who have never smoked	8 476 947 (46)	499 737 (50)	7 029 399 (47)	418 809 (48.8)	1 456 293 (41.9)	59 601 (39.8)
	People who previously smoked	6 043 497 (32.8)	324 345 (32.4)	5 374 977 (36)	330 483 (38.5)	698 025 (20.1)	43 875 (29.3)
	People who smoke	3 144 591 (17.1)	135 819 (13.6)	2 055 999 (13.8)	93 303 (10.9)	927 405 (26.7)	37 749 (25.2)
Region	East	4 254 147 (23.1)	224 865 (22.5)	3 479 745 (23.3)	180 867 (21.1)	781 797 (22.5)	33 747 (22.6)
	East Midlands	3 222 339 (17.5)	189 603 (19)	2 645 907 (17.7)	161 463 (18.8)	569 937 (16.4)	28 839 (19.3)
	London	1 235 547 (6.7)	67 149 (6.7)	792 069 (5.3)	37 185 (4.3)	479 103 (13.8)	11 055 (7.4)
	North East	877 989 (4.8)	59 589 (6)	718 257 (4.8)	48 267 (5.6)	150 165 (4.3)	7209 (4.8)
	North West	1 636 665 (8.9)	108 531 (10.8)	1 375 317 (9.2)	87 153 (10.1)	244 155 (7)	12 057 (8.1)
	South East	1 233 825 (6.7)	49 773 (5)	1 015 635 (6.8)	56 415 (6.6)	206 403 (5.9)	8853 (5.9)
	South West	2 620 803 (14.2)	79 635 (8)	2 283 933 (15.3)	131 631 (15.3)	349 923 (10.1)	17 835 (11.9)
	West Midlands	752 241 (4.1)	55 737 (5.6)	547 257 (3.7)	29 613 (3.4)	198 651 (5.7)	8193 (5.5)
	Yorkshire/Humber	2 589 219 (14.1)	165 549 (16.5)	2 090 595 (14)	126 297 (14.7)	498 903 (14.3)	21 849 (14.6)
Care home resident		88 563 (0.5)	15 057 (1.5)	78 591 (0.5)	2907 (0.3)	3633 (0.1)	111 (0.1)

1: most deprived, 5: least deprived. Median (IQR) age was 49 (34–64) years in the prevaccination cohort, 53 (38–67) in the vaccinated cohort and 35 (27–46) in the unvaccinated cohort.

IMD, Index of Multiple Deprivation.

### Overall incidence

In each cohort, the incidence of gastrointestinal disease was higher after a diagnosis of COVID-19 than before or without a COVID-19 diagnosis, with the highest incidence rates after hospitalised COVID-19 (table 2). In the prevaccination cohort, the total numbers of events (total after COVID-19) were: upper gastrointestinal bleeding 143 769 (7764), lower gastrointestinal bleeding 177 909 (8526), acute pancreatitis 26 937 (1314), appendicitis 27 801 (1290), gastro-oesophageal reflux 413 727 (19 860), dyspepsia 165 399 (7254), peptic ulcer 24 957 (1122), non-alcoholic steatohepatitis 4443 (300), gallstones 152 367 (7164) and IBS 151 527 (7314).

**Table 2:** Number of gastrointestinal disease events in prevaccination, vaccinated and unvaccinated cohorts, with person-years of follow-up, by COVID-19 severity

Event	COVID-19 severity	Prevaccination cohort (1 January 2020 to 14 December 2021)		Vaccinated cohort (1 June to 14 December 2021)		Unvaccinated cohort (1 June to 14 December 2021)	
		Event/Person-years	Incidence rate*	Event/Person-years	Incidence rate*	Event/Person-years	Incidence rate*
Upper gastrointestinal bleeding	No COVID-19	136 005/33 831 788	402	33 747/6 268 653	538	2973/1 208 490	246
	Hospitalised COVID-19	1857/47 386	3919	327/2640	12 386	135/1801	7495
	Non-hospitalised COVID-19	5907/947 899	623	1131/156 814	721	165/25 292	652
Lower gastrointestinal bleeding	No COVID-19	169 383/33 796 400	501	40 947/6 268 304	653	3537/1 207 169	293
	Hospitalised COVID-19	1539/47 541	3237	273/2646	10 317	99/1808	5475
	Non-hospitalised COVID-19	6987/946 159	738	1299/156 730	829	177/25 285	700
Acute pancreatitis	No COVID-19	25 623/33 911 364	76	6597/6 276 227	105	825/1 207 345	68
	Hospitalised COVID-19	189/48 622	389	39/2686	1452	15/1822	823
	Non-hospitalised COVID-19	1125/952 428	118	243/157 043	155	45/25 314	178
Appendicitis	No COVID-19	26 511/33 911 143	78	4869/6 278 274	78	657/1 209 390	54
	Hospitalised COVID-19	69/48 737	142	3/2692	111	3/1825	164
	Non-hospitalised COVID-19	1221/952 017	128	225/157 043	143	39/25 316	154
Gastro-oesophageal reflux	No COVID-19	393 867/33 604 241	1172	111 645/6 251 470	1786	7605/1 208 409	629
	Hospitalised COVID-19	4365/45 118	9675	909/2525	35 996	405/1738	23 297
	Non-hospitalised COVID-19	15 495/936 857	1654	3099/156 165	1984	375/25 230	1486
Dyspepsia	No COVID-19	158 145/33 793 838	468	35 181/6 269 347	561	3699/1 209 307	306
	Hospitalised COVID-19	837/47 889	1748	99/2672	3706	75/1808	4148
	Non-hospitalised COVID-19	6417/945 661	679	975/156 776	622	189/25 271	748
Peptic ulcer	No COVID-19	23 835/33 904 867	70	6243/6 267 249	100	501/1 209 408	41
	Hospitalised COVID-19	183/48 634	376	33/2686	1228	15/1823	823
	Non-hospitalised COVID-19	939/952 713	99	177/157 060	113	21/25 319	83
Non-alcoholic steatohepatitis	No COVID-19	4143/33 925 228	12	1101/6 271 285	18	63/1 209 509	5
	Hospitalised COVID-19	87/48 771	178	15/2691	557	9/1823	494
	Non-hospitalised COVID-19	213/953 504	22	33/157 099	21	3/25 324	12
Gallstones	No COVID-19	145 203/33 814 998	429	42 717/6 265 904	682	3 105/1 208 687	257
	Hospitalised COVID-19	1371/47 568	2882	279/2638	10 575	105/1802	5826
	Non-hospitalised COVID-19	5793/947 292	612	1179/156 736	752	171/25 283	676
Irritable bowel syndrome	No COVID-19	144 213/33 815 506	426	37 935/6 269 432	605	3873/1 209 319	320
	Hospitalised COVID-19	1053/47 897	2198	261/2644	9871	177/1788	9898
	Non-hospitalised COVID-19	6261/946 670	661	1173/156 726	748	195/25 276	771

We rounded all counts to the nearest six then subtracted three to obscure counts. Counts less than six therefore appear as ‘3’. Counts of zero are maintained and considered structural for this output.

* Incidence rates are per 100 000 person-years.

### Comparisons of event rates after COVID-19 diagnosis versus before or without COVID-19

Maximally adjusted HRs (aHRs) comparing the incidence of each outcome post-COVID-19 with incidence before or without COVID-19 did not differ substantially from age-adjusted and sex-adjusted HRs in any cohort (table 3, online supplemental table 4). Incidence of all outcomes was extremely high on day 0 (table 3).

**Table 3:** Adjusted HRs (95% CI) comparing the incidence of gastrointestinal disease events after COVID-19 with the incidence before or without COVID-19, in the prevaccination, vaccinated and unvaccinated cohorts

Outcome	Time since COVID-19	Prevaccination cohort	Vaccinated cohort	Unvaccinated cohort
Upper gastrointestinal bleeding	Day 0	177 (168 to 186)	52.7 (48.3 to 57.6)	106 (87.6 to 128)
	Weeks 1–4	3.77 (3.53 to 4.03)	1.84 (1.67 to 2.03)	4.26 (3.51 to 5.19)
	Weeks 5–28	1.59 (1.52 to 1.65)	1.19 (1.10 to 1.30)	1.40 (1.10 to 1.77)
	Weeks 29–52	1.29 (1.23 to 1.35)	–	–
	Weeks 53–102	1.43 (1.33 to 1.54)	–	–
Lower gastrointestinal bleeding	Day 0	91.6 (86.5 to 97.1)	29.3 (26.5 to 32.4)	55.5 (44.5 to 69.2)
	Weeks 1–4	2.47 (2.31 to 2.65)	1.41 (1.28 to 1.55)	2.85 (2.32 to 3.50)
	Weeks 5–28	1.46 (1.41 to 1.52)	1.19 (1.11 to 1.28)	1.34 (1.09 to 1.65)
	Weeks 29–52	1.25 (1.20 to 1.30)	–	–
	Weeks 53–102	1.35 (1.26 to 1.45)	–	–
Acute pancreatitis	Day 0	109 (94.7 to 126)	42.1 (34.1 to 52.1)	50.0 (31.4 to 79.4)
	Weeks 1–4	2.80 (2.38 to 3.30)	1.70 (1.37 to 2.11)	2.75 (1.82 to 4.16)
	Weeks 5–28	1.33 (1.21 to 1.47)	1.12 (0.92 to 1.35)	1.14 (0.72 to 1.80)
	Weeks 29–52	1.22 (1.10 to 1.36)	–	–
	Weeks 53–102	1.63 (1.39 to 1.92)	–	–
Appendicitis	Day 0	50.5 (42.2 to 60.4)	17.7 (12.8 to 24.5)	31.0 (16.1 to 59.5)
	Weeks 1–4	1.79 (1.49 to 2.16)	1.59 (1.26 to 2.00)	2.43 (1.48 to 4.01)
	Weeks 5–28	1.23 (1.13 to 1.34)	1.30 (1.08 to 1.57)	1.78 (1.13 to 2.81)
	Weeks 29–52	1.18 (1.07 to 1.30)	–	–
	Weeks 53–102	1.24 (1.02 to 1.50)	–	–
Gastro-oesophageal reflux	Day 0	70.8 (67.8 to 74.0)	18.6 (17.3 to 20.1)	55.9 (48.5 to 64.5)
	Weeks 1–4	3.36 (3.23 to 3.50)	1.42 (1.34 to 1.51)	3.81 (3.39 to 4.29)
	Weeks 5–28	1.35 (1.32 to 1.38)	1.20 (1.15 to 1.25)	1.65 (1.46 to 1.87)
	Weeks 29–52	1.21 (1.18 to 1.24)	–	–
	Weeks 53–102	1.33 (1.28 to 1.39)	–	–
Dyspepsia	Day 0	28.4 (25.7 to 31.4)	7.31 (5.98 to 8.94)	31.3 (24.2 to 40.5)
	Weeks 1–4	1.65 (1.52 to 1.79)	1.04 (0.93 to 1.16)	2.21 (1.79 to 2.73)
	Weeks 5–28	1.25 (1.21 to 1.30)	1.13 (1.04 to 1.22)	1.53 (1.27 to 1.84)
	Weeks 29–52	1.22 (1.17 to 1.27)	–	–
	Weeks 53–102	1.25 (1.16 to 1.34)	–	–
Peptic ulcer	Day 0	50.4 (40.4 to 63.0)	15.9 (11.2 to 22.6)	33.2 (14.8 to 74.8)
	Weeks 1–4	4.38 (3.75 to 5.12)	2.20 (1.78 to 2.73)	2.97 (1.66 to 5.31)
	Weeks 5–28	1.73 (1.57 to 1.90)	1.20 (0.99 to 1.47)	1.97 (1.18 to 3.31)
	Weeks 29–52	1.30 (1.16 to 1.45)	–	–
	Weeks 53–102	1.30 (1.08 to 1.57)	–	–
Non-alcoholic steatohepatitis	Day 0	258 (198 to 337)	26.4 (14.5 to 48.0)	X
	Weeks 1–4	5.89 (4.01 to 8.66)	2.17 (1.40 to 3.36)	X
	Weeks 5–28	1.57 (1.05 to 2.35)	0.93 (0.59 to 1.48)	X
	Weeks 29–52	1.10 (0.73 to 1.65)	–	–
	Weeks 53–102	1.75 (0.95 to 3.22)	–	–
Gallstones	Day 0	98.9 (92.8 to 105)	26.3 (23.6 to 29.2)	41.9 (32.5 to 54.2)
	Weeks 1–4	2.73 (2.53 to 2.94)	1.38 (1.25 to 1.52)	2.89 (2.34 to 3.57)
	Weeks 5–28	1.45 (1.39 to 1.51)	1.10 (1.01 to 1.18)	1.72 (1.41 to 2.09)
	Weeks 29–52	1.21 (1.16 to 1.26)	–	–
	Weeks 53–102	1.35 (1.25 to 1.45)	–	–
Irritable bowel syndrome	Day 0	47.5 (44.0 to 51.3)	13.7 (12.1 to 15.5)	37.1 (29.8 to 46.2)
	Weeks 1–4	2.20 (2.04 to 2.37)	1.17 (1.06 to 1.29)	3.39 (2.90 to 3.96)
	Weeks 5–28	1.14 (1.10 to 1.19)	1.09 (1.01 to 1.16)	1.38 (1.15 to 1.65)
	Weeks 29–52	1.12 (1.08 to 1.17)	–	–
	Weeks 53–102	1.15 (1.07 to 1.23)	–	–

HRs are maximally adjusted.

X represents insufficient events for estimation.

#### Upper gastrointestinal bleeding

Incidence of upper gastrointestinal bleeding was elevated during weeks 1–4 post-COVID-19, compared with before or without COVID-19: aHRs were 3.77 (95% CI 3.53 to 4.03) in the prevaccination cohort, 1.84 (1.67 to 2.03) in the vaccinated cohort and 4.26 (3.51 to 5.19) in the unvaccinated cohort (figure 1, table 3). In the prevaccination cohort, incidence remained elevated through weeks 53–102: aHR 1.43 (95% CI 1.33 to 1.54). During weeks 1–4, aHRs were substantially higher after hospitalisation with COVID-19 (prevaccination: 20.1 (18.4 to 22.1); vaccinated: 15.2 (13.4 to 17.3); unvaccinated: 19.7 (15.0 to 25.9)) than after non-hospitalised COVID-19 (prevaccination: 1.92 (1.74 to 2.11); vaccinated: 1.20 (1.06 to 1.36); unvaccinated: 2.14 (1.61 to 2.85)) (figure 1, online supplemental tables 5 and 6). In the prevaccination cohort, aHRs remained higher after hospitalised than non-hospitalised COVID-19 throughout follow-up.

**Figure 1:**
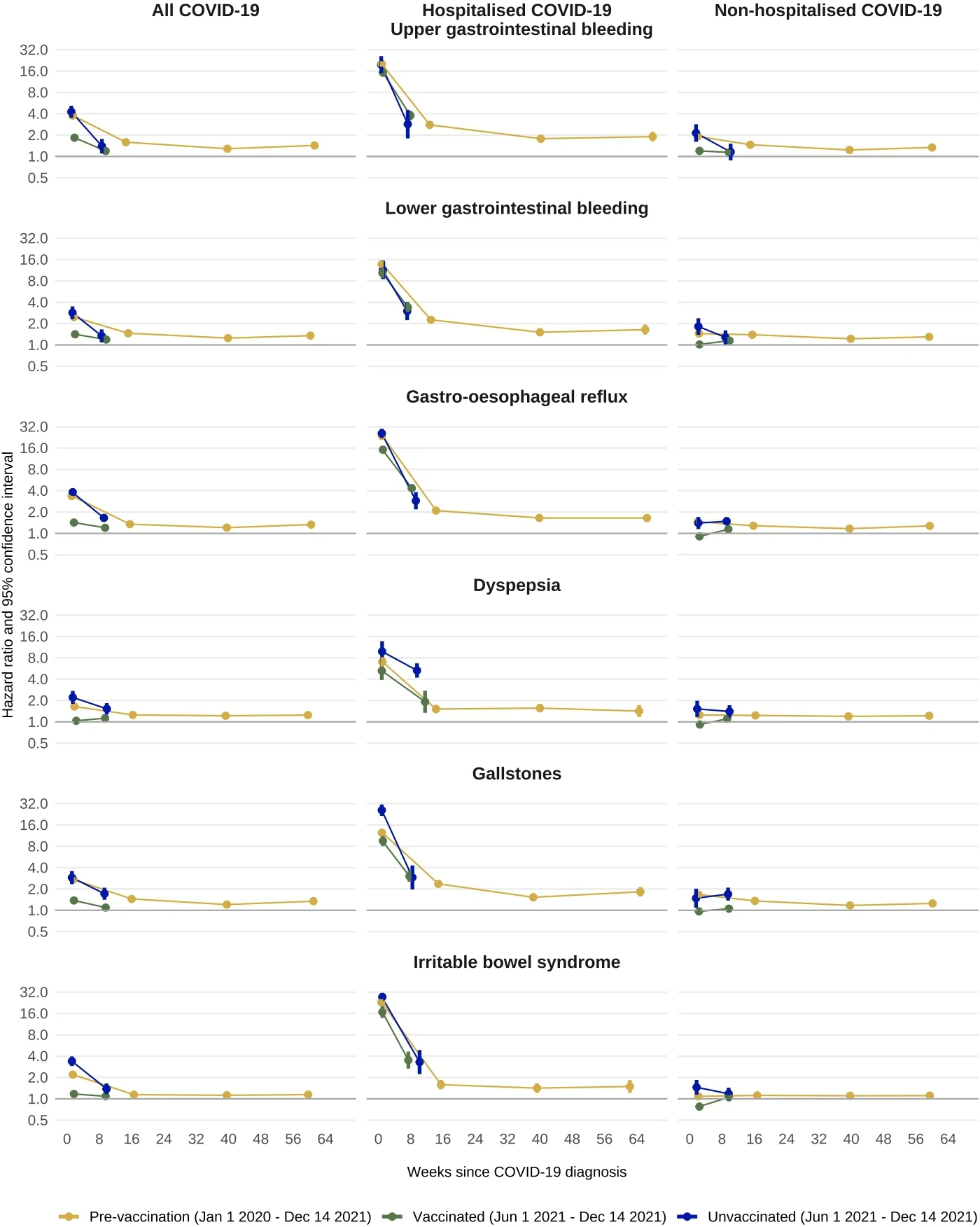
Maximally adjusted HRs and 95% CIs comparing the incidence of gastrointestinal disease events after COVID-19 with the incidence before or without COVID-19, in the prevaccination, vaccinated and unvaccinated cohorts, overall and by COVID-19 severity with day 0 excluded. Across cohorts, estimates were highest in the early weeks after diagnosis, particularly following hospitalised COVID-19, and declined over time, although remaining elevated in several outcomes.

#### Lower gastrointestinal bleeding

Across all cohorts, the incidence of lower gastrointestinal bleeding was elevated during weeks 1–4 post-COVID-19 compared with before or without COVID-19: prevaccination: 2.47 (95% CI 2.31 to 2.65); vaccinated: 1.41 (1.28 to 1.55); unvaccinated: 2.85 (2.32 to 3.50) (figure 1, table 3). In the prevaccination cohort, incidence remained elevated through weeks 53–102 (aHR 1.35 (1.26 to 1.45)). During weeks 1–4, aHRs were substantially higher after hospitalised COVID-19 (prevaccination: 13.7 (12.4 to 15.2); vaccinated: 10.4 (8.80 to 12.2); unvaccinated: 11.4 (8.48 to 15.4)) than after non-hospitalised COVID-19 (prevaccination: 1.44 (1.32 to 1.59); vaccinated: 1.02 (0.91 to 1.14); unvaccinated: 1.82 (1.39 to 2.38)) (figure 1, online supplemental tables 5 and 6). In the prevaccination cohort, aHRs remained higher after hospitalised than non-hospitalised COVID-19 throughout follow-up.

#### Subgroup analysis

Subgroup analyses showed notable variation in aHRs post-COVID-19 by age and by prior gastrointestinal events, whereas differences by sex, ethnicity, and prior gastrointestinal operations were minimal (online supplemental tables 5–18, online supplemental figures 5–9). aHRs were consistently higher in older versus younger age groups (online supplemental tables 7–10, online supplemental figure 5). For example, in the prevaccination cohort during weeks 1–4, aHRs for upper gastrointestinal bleeding rose from 1.69 (95% CI 1.40 to 2.04) in individuals aged 18–39 years to 5.04 (4.37 to 5.81) in those aged 60–79 years. A similar pattern was seen for lower gastrointestinal bleeding, with aHRs increasing from 1.16 (0.99 to 1.37) to 3.96 (3.49 to 4.49) across the same age groups. These age-related differences persisted through weeks 5–28 postdiagnosis.

aHRs were higher for individuals without than with prior gastrointestinal events (online supplemental tables 15 and 16, online supplemental figure 8). In the prevaccination cohort, for upper gastrointestinal bleeding during weeks 1–4, the aHRs for upper gastrointestinal bleeding during weeks 1–4 were 5.36 (95% CI 4.78 to 6.01) and 3.36 (3.09 to 3.64) among those without and with prior events, respectively. Similar patterns were observed for lower gastrointestinal bleeding (without prior event: 3.00 (2.65 to 3.40); with prior event: 2.31 (2.13 to 2.51)).

#### Other gastrointestinal diseases

aHRs for other gastrointestinal diseases were broadly similar to those for upper and lower gastrointestinal bleeding, both overall (table 3) and by COVID-19 severity (figure 1, online supplemental tables 5 and 6). As with upper and lower gastrointestinal bleeding, aHRs for these diseases were attenuated in the vaccinated compared with the prevaccination and unvaccinated cohorts during weeks 1–4 post-COVID-19.

### Absolute excess risk

Estimated excess risks of upper gastrointestinal bleeding 28 weeks after COVID-19, standardised to the age distribution of the prevaccination cohort, were 159 per 100 000 people diagnosed with COVID-19 in the prevaccination cohort, 60 in the vaccinated cohort and 112 in the unvaccinated cohort (online supplemental table 19, online supplemental figure 10). For lower gastrointestinal bleeding, estimated excess risks 28 weeks post-COVID-19 were 144 per 100 000 people diagnosed with COVID-19 in the prevaccination cohort, 64 in the vaccinated cohort and 91 in the unvaccinated cohort (online supplemental table 19, online supplemental figure 11). In all cohorts, for upper and lower gastrointestinal bleeding, excess risks increased with age: the oldest group (80–110 years) consistently had the highest risks, while the youngest group (18–39 years) had the lowest. Similar patterns were observed for other gastrointestinal outcomes (online supplemental figures 12–15).

## Discussion

### Main findings

In this population-based study of over 18 million people followed for up to 2 years, COVID-19 was associated with marked elevations in the incidence of gastrointestinal diseases during the first month after diagnosis in the prevaccination and unvaccinated cohorts. While associations declined with increasing time since COVID-19 diagnosis, the incidence of gastrointestinal disease remained higher for up to 2 years post-COVID-19 diagnosis, particularly among hospitalised patients, in the prevaccination cohort and for 6 months in the unvaccinated cohort. Incidence of gastrointestinal diseases after COVID-19 was elevated in the vaccinated cohort, but to a lesser extent than in the other cohorts. Overall differences between the vaccinated and unvaccinated cohorts were largely explained by whether COVID-19 led to hospitalisation, with the elevation in incidence of gastrointestinal diseases much greater after hospitalised than non-hospitalised COVID-19 in all cohorts. Among vaccinated individuals, there was little elevation in the incidence of gastrointestinal diseases after non-hospitalised COVID-19. Patterns of association were similar across all gastrointestinal diseases studied.

### Comparison with literature

Our findings are consistent with previous studies reporting an association of COVID-19 diagnosis with higher rates of long-term gastrointestinal diseases, including gastro-oesophageal reflux, dyspepsia, IBS, gastrointestinal bleeding and acute pancreatitis.^12,20,26–28^ We also observed associations with gallstones, peptic ulcer and non-alcoholic steatohepatitis, consistent with results reported in recent studies and meta-analyses.^4,13,19,26^ Another study reported gastrointestinal manifestations in the acute phase of infection across multiple cohorts.^13^ Additionally, systematic reviews have reported gastrointestinal bleeding following COVID-19, particularly among hospitalised individuals receiving anticoagulants, with prevalence ranging between 0.5% and 3% depending on disease severity.^26,27^

Our findings are consistent with those of Xu *et al*,^18^ who identified an increased risk of a broad range of gastrointestinal diseases post-COVID-19, particularly among those with severe illness, using US Veterans Affairs data. However, that cohort was composed mostly of older unvaccinated men, limiting generalisability. Our study used a large, nationally representative dataset from England, and accounted for vaccination status, COVID-19 severity and different population subgroups.

The attenuation of post-COVID-19 gastrointestinal rates among vaccinated individuals is likely due to a reduction of disease severity following immunisation, consistent with studies showing lower risk of long covid among vaccinated people.^22,29^ Breakthrough infection studies confirm that vaccinated individuals can still develop gastrointestinal symptoms, although the risk remains lower compared with unvaccinated individuals.^30^ This indicates that, while vaccination is unlikely to fully prevent post-COVID-19 gastrointestinal complications, it may substantially reduce the risk of these outcomes.

### Clinical implications

Findings from this study are consistent with COVID-19 being a systemic illness that affects multiple systems. Severely affected patients are more likely to have acute gastrointestinal diseases, including upper and lower gastrointestinal bleeding. Our results further highlight the importance of vaccination in reducing the risk of severe COVID-19, and thus the subsequent risk of long-term harm.

### Strengths and limitations

Strengths of this study include the large and representative study population, the availability of detailed linked EHR data, relatively long follow-up and the opportunity to examine the role of vaccination in the relationship between COVID-19 and gastrointestinal diseases.

We also note several limitations. First, while OpenSAFELY TPP is broadly representative of the English population,^31^ geographic variation in coverage may limit generalisability. In our models, we mitigated coverage-related differences by stratifying by region and adjusting for sex, age, ethnicity and deprivation. Clinical phenotypes were not externally validated and there may be some misclassification due to differences in coding practices across health services. However, code lists were defined using search terms and clinically reviewed, in line with best practice for observational EHR research. Additionally, differential healthcare contact may have affected outcome ascertainment. Unvaccinated individuals may have been less likely to access health services and to undergo COVID-19 testing, potentially leading to underestimation of an effect in those not hospitalised with COVID-19. In contrast, those with recorded COVID-19, particularly those hospitalised, may have been more likely to have gastrointestinal disease more frequently recorded due to increased healthcare contact, especially for non-acute outcomes, such as gallstones and non-alcoholic steatohepatitis. This may partially explain the high HRs observed shortly after diagnosis and their subsequent attenuation. However, such issues alone are unlikely to account for the persistent elevation in risk following hospitalised COVID-19 or the heterogeneity of associations across conditions.

Conversely, underascertainment of gastrointestinal diseases due to reduced use of primary care services during the pandemic and cessation of normal screening activities, together with unaccounted competing risks such as other hospitalisations or infections, may have influenced healthcare contact and outcome ascertainment.

A further limitation is the potential misclassification of COVID-19 exposure, reflecting variations in testing practices, absence of symptom data and incomplete capture of untested infections, which is likely to have biased the estimates towards the null. Characterisation of COVID-19 severity was restricted to hospital admissions due to limited information on symptom status in primary care data. Individual-level data on COVID-19 variants were also unavailable. However, the vaccinated and unvaccinated cohorts were aligned to a period dominated by the Delta variant, enhancing comparability, whereas the prevaccination cohort reflected an early phase of the pandemic, when wild-type and Alpha strains dominated, and was therefore not directly comparable with the vaccinated and unvaccinated cohorts.

In the vaccinated cohort, we did not account for time since vaccination, which may have modified the observed association. However, protection against severe COVID-19 generally persists for up to 6 months after a second dose,^32^ and the average follow-up in the vaccinated cohort was 5.5 months, suggesting limited impact from waning immunity. In secondary care, ICD-10 codes recorded in any diagnostic position were used to maximise capture, which may reduce specificity for some outcomes. Differential ascertainment or recording of gastrointestinal diagnoses around acute COVID-19 care (particularly during hospitalisation), which could inflate the estimates, cannot be excluded. Finally, residual confounding is possible despite adjustment for a wide range of demographic characteristics and prior morbidities, for example, obesity was measured using a binary indicator and may not fully capture variations in adiposity.

## Conclusions

The incidence of gastrointestinal disease was elevated for up to 2 years among individuals hospitalised following a COVID-19 diagnosis, especially in the first few weeks. Our findings are consistent with a role for COVID-19 vaccination in mitigating long-term gastrointestinal effects following COVID-19.

### Information governance

NHS England is the data controller of the NHS England OpenSAFELY COVID-19 Service; TPP is the data processor; all study authors using OpenSAFELY have the approval of NHS England.^33^ This implementation of OpenSAFELY is hosted within the TPP environment, which is accredited to the ISO 27001 information security standard and is NHS IG Toolkit compliant.^34^ Patient data has been pseudonymised for analysis and linkage using industry standard cryptographic hashing techniques; all pseudonymised datasets transmitted for linkage onto OpenSAFELY are encrypted; access to the NHS England OpenSAFELY COVID-19 service is via a virtual private network (VPN) connection; the researchers hold contracts with NHS England and only access the platform to initiate database queries and statistical models; all database activity is logged; only aggregate statistical outputs leave the platform environment following best practice for anonymisation of results such as statistical disclosure control for low cell counts.^35^

The service adheres to the obligations of the UK General Data Protection Regulation and the Data Protection Act 2018. The service previously operated under notices initially issued in February 2020 by the Secretary of State under Regulation 3(4) of the Health Service (Control of Patient Information) Regulations 2002, which required organisations to process confidential patient information for COVID-19 purposes; this set aside the requirement for patient consent.^36^As of 1 July 2023, the Secretary of State has requested that NHS England continue to operate the Service under the COVID-19 Directions 2020.^37^ In some cases of data sharing, the common law duty of confidence is met using, for example, patient consent or support from the Health Research Authority Confidentiality Advisory Group.^38^

Taken together, these provide the legal bases to link patient datasets using the service. GP practices, which provide access to the primary care data, are required to share relevant health information to support the public health response to the pandemic, and have been informed of how the service operates.

## Supplementary material

10.1136/bmjph-2025-004709 online supplemental file 1

10.1136/bmjph-2025-004709 online supplemental file 2

## Data Availability

Data may be obtained from a third party and are not publicly available.
