# Supplementary material for: Impact of COVID-19 on gastrointestinal disease in vaccinated and unvaccinated individuals: a population-based cohort study in England
Source: BMJ Public Health. 2026 Oct 20;4(4):e004709. doi: 10.1136/bmjph-2025-004709 (PMC13691888; doi:10.1136/bmjph-2025-004709)

The impact of COVID-19 on gastrointestinal disease in vaccinated and unvaccinated individuals: a population-based cohort study in England

Supplementary Figures

List of Figures

Supplementary Figure 1: COVID-19 cases over time . . . 2
Supplementary Figure 2: Gastrointestinal Bleeding DAG . . . 3
Supplementary Figure 3: Study design . . . 4
Supplementary Figure 4: Consort Diagram . . . 5
Supplementary Figure 5: Gastrointestinal outcomes by age . . . 6
Supplementary Figure 6: Gastrointestinal outcomes by sex . . . 7
Supplementary Figure 7: Gastrointestinal outcomes by ethnicity . . . 8
Supplementary Figure 8: Gastrointestinal outcomes by prior operations . . . 9
Supplementary Figure 9: Gastrointestinal outcomes by prior events . . . 10
Supplementary Figure 10: Upper Gastrointestinal bleeding risk . . . 11
Supplementary Figure 11: Lower Gastrointestinal bleeding risk . . . 12
Supplementary Figure 12: Gastrointetinal reflux absolute excess risk . . . 13
Supplementary Figure 13: Dyspepsia absolute excess risk . . . 14
Supplementary Figure 14: Gallstones absolute excess risk . . . 15
Supplementary Figure 15: IBS absolute excess risk . . . 16

Supplementary Figure 1. COVID-19 cases over time. Reproduced from Walker et al., 2024 (PMID: 39167370).

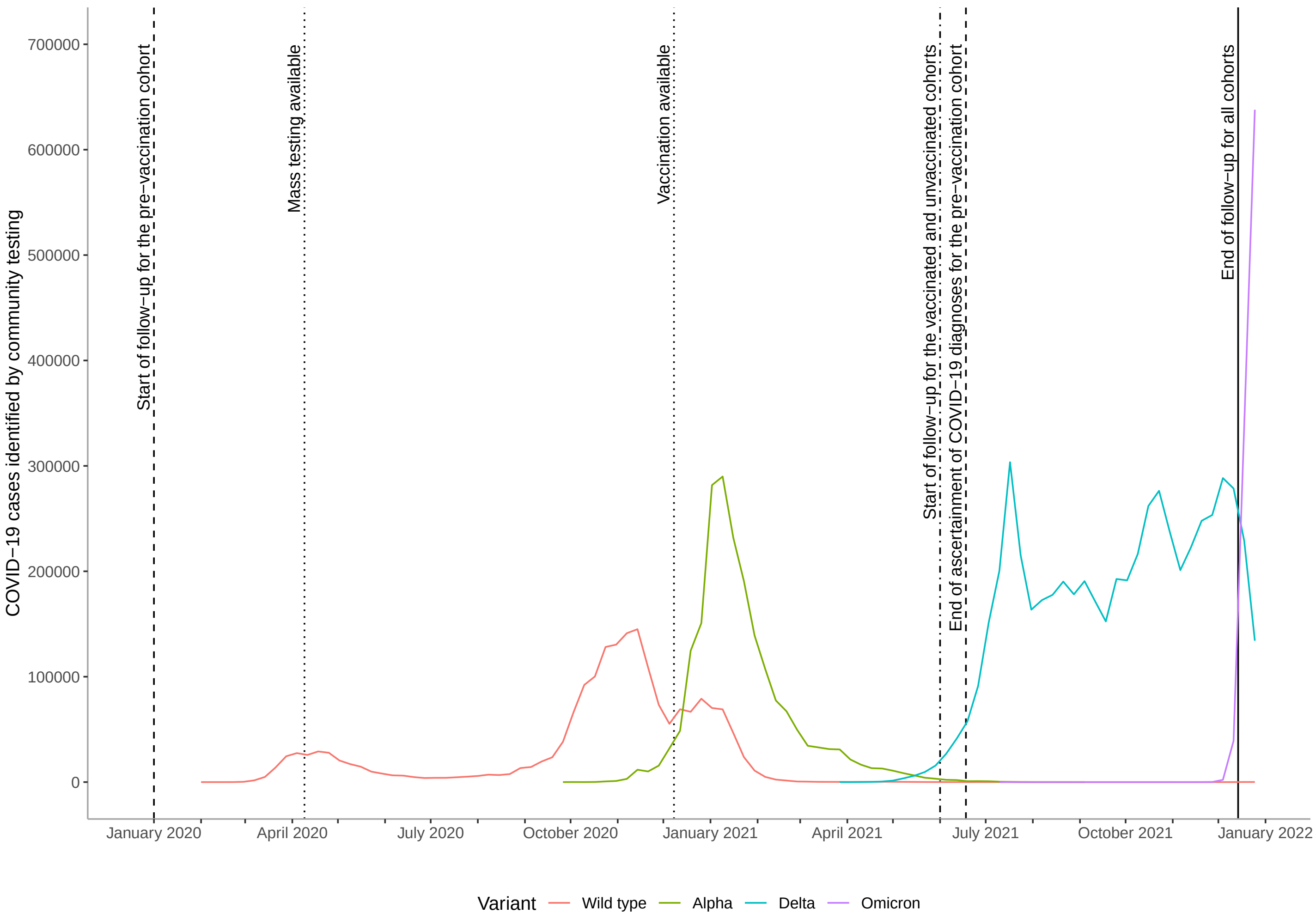

Supplementary Figure 2. Gastrointestinal Bleeding Directed Acyclic Graph (DAG).

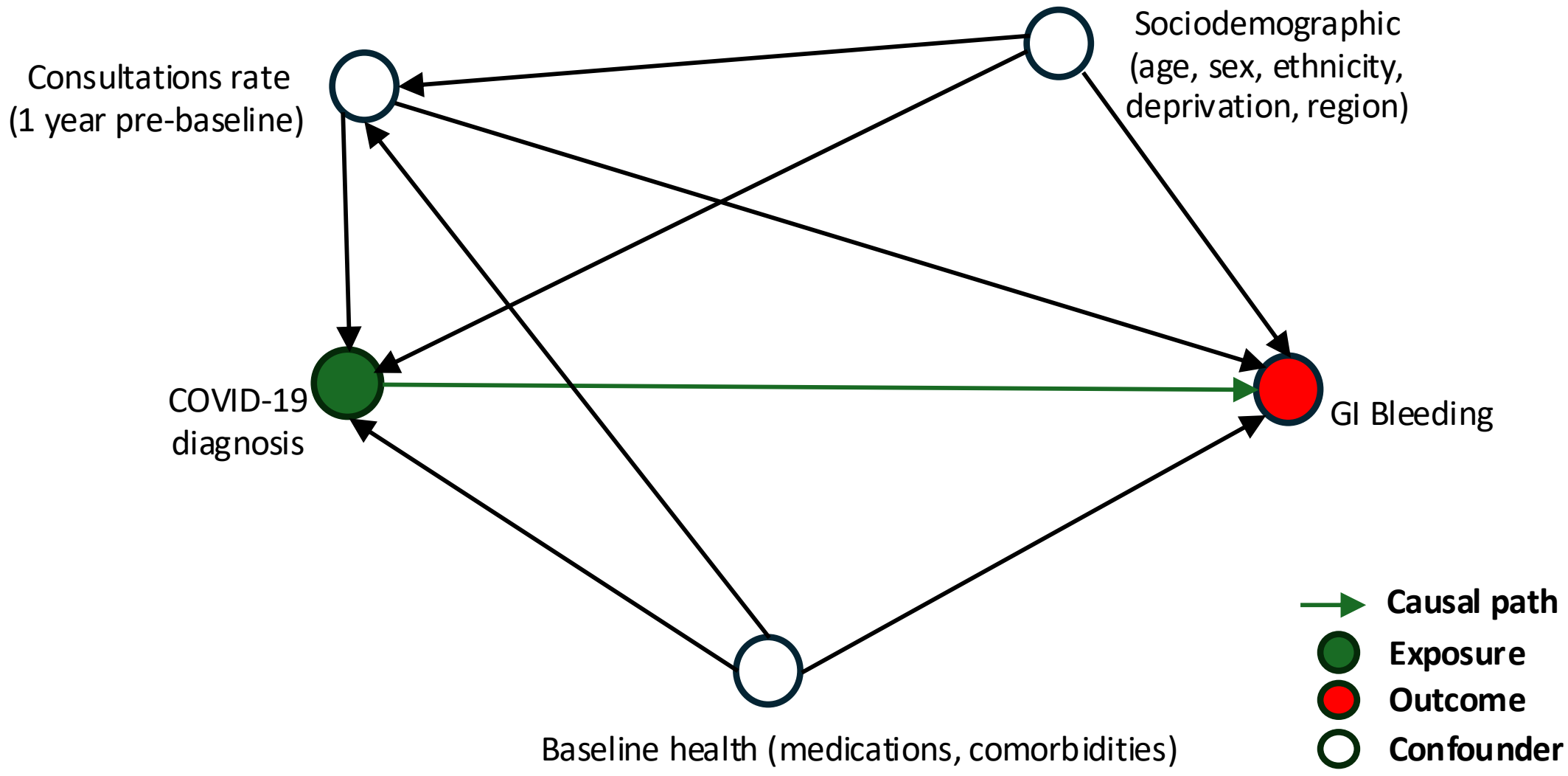

Supplementary Figure 3. Study design over time for Pre-vaccination, vaccinated and unvaccinated cohorts. ► Earliest of: death; outcome; end of exposure; deregistration; vaccination; or JCVI eligibility date. ►► Earliest of: death; outcome event; study end date; or deregistration. * Earliest of: death; outcome event; study end; or deregistration. ** Earliest of: death; outcome event; study end; or deregistration. ■ Earliest of: death; outcome event; study end; deregistration; or vaccination. ■■ Earliest of: death; outcome event; study end; or deregistration.

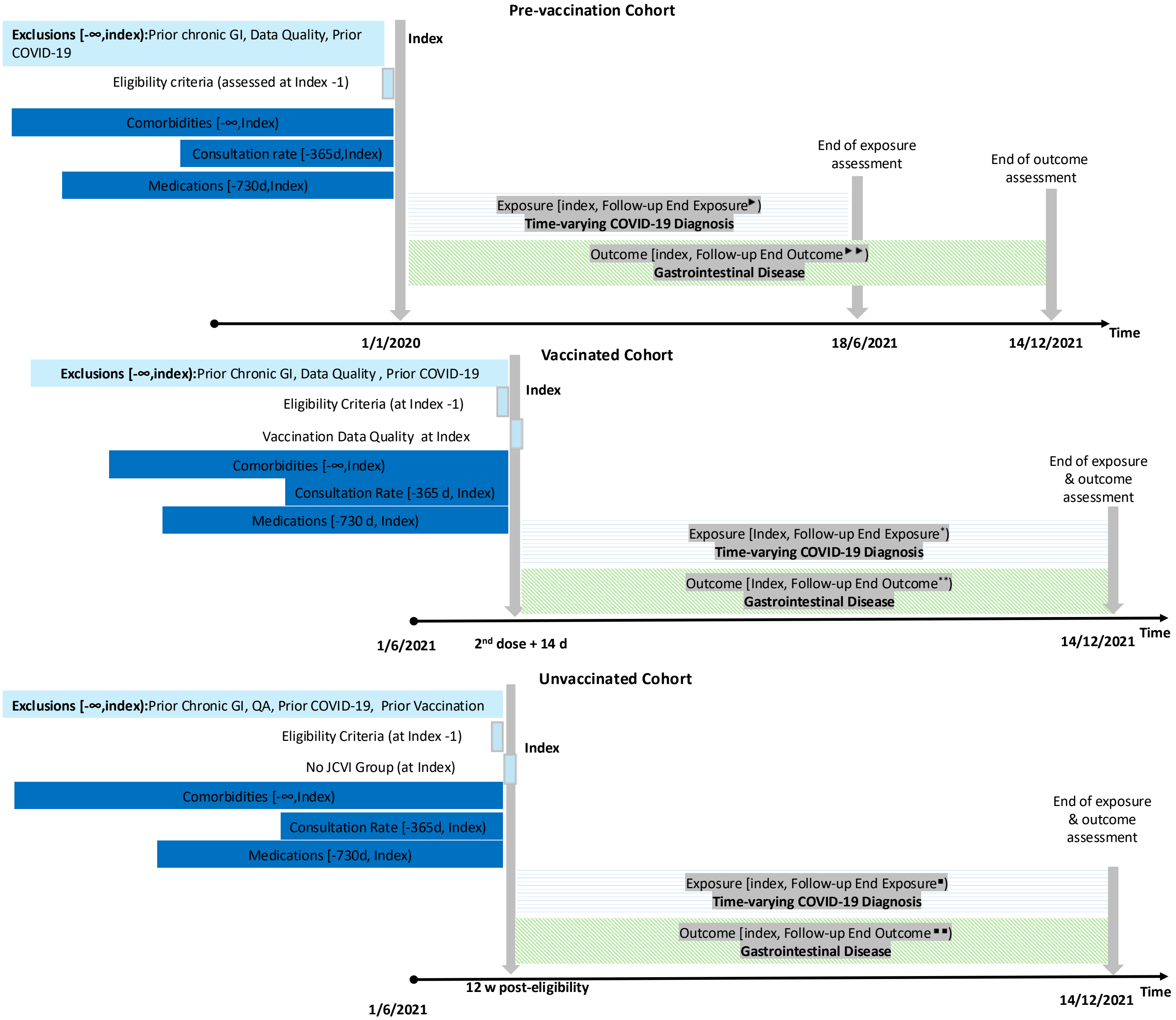

Supplementary Figure 4. Diagram showing cohort construction. * Vaccinated cohort: two-dose (≥21 days apart), no mixed products before 7 May 2021, index within study period. ** Unvaccinated cohort: no vaccination prior to index date and valid JCVI priority group.

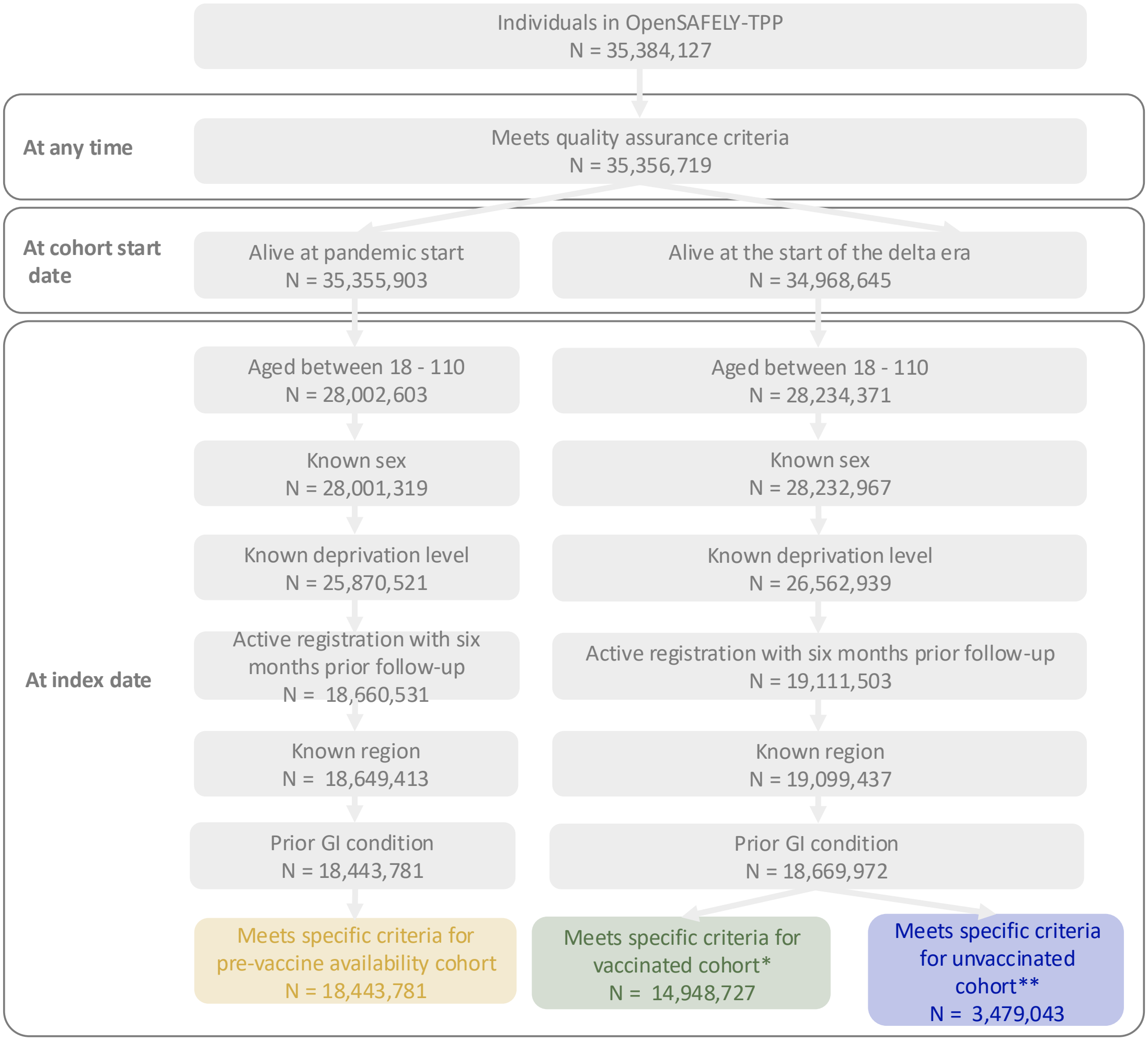

Supplementary Figure 5. Maximally adjusted hazard ratios and 95% CIs comparing the incidence of GI outcome diseases after COVID-19 with the incidence before or without COVID-19, in the pre-vaccination, vaccinated and unvaccinated cohorts, by age group.

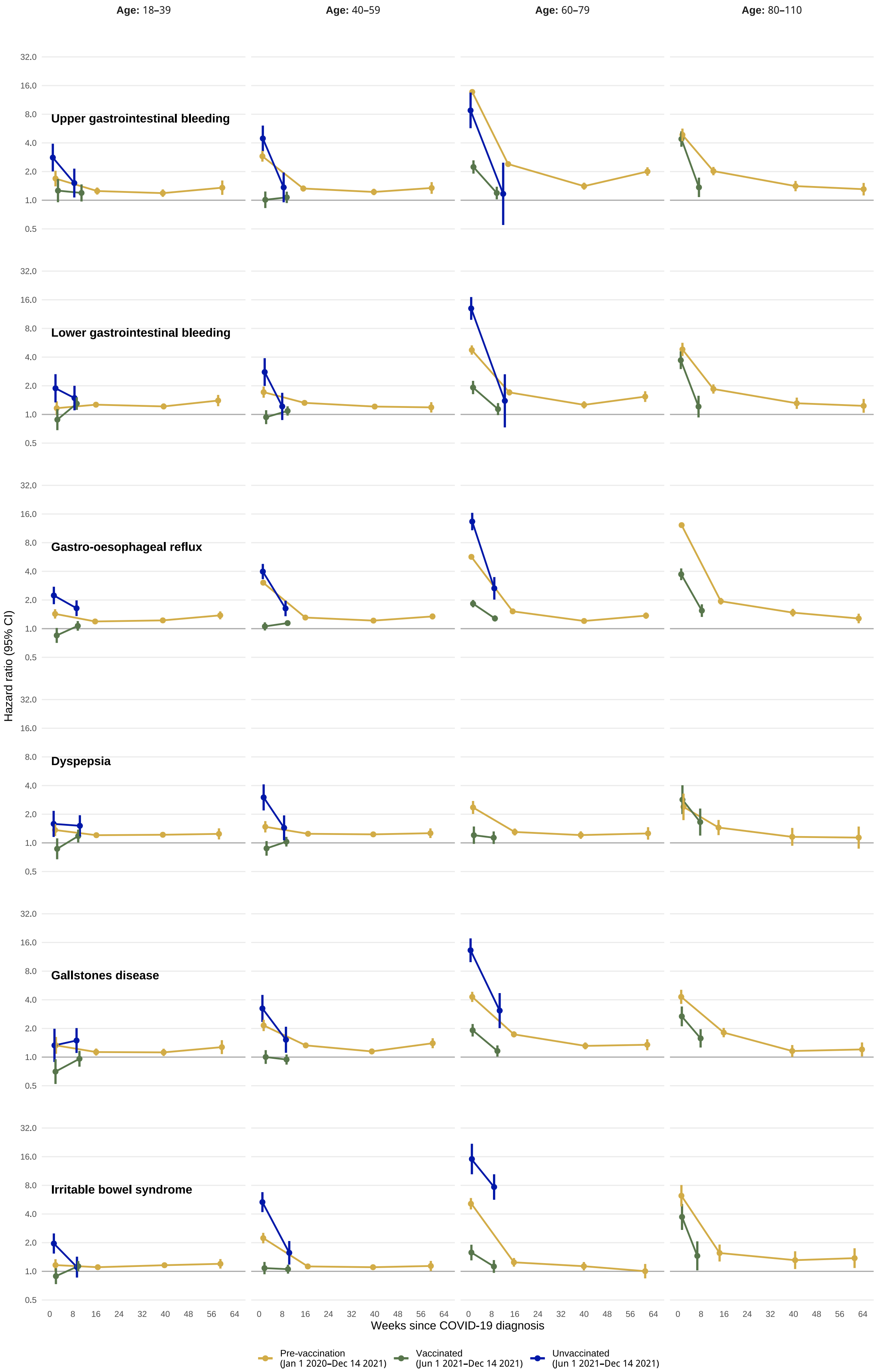

Supplementary Figure 6. Maximally adjusted hazard ratios and 95% CIs comparing the incidence of GI outcome diseases after COVID-19 with the incidence before or without COVID-19, in the pre-vaccination, vaccinated and unvaccinated cohorts, by sex.

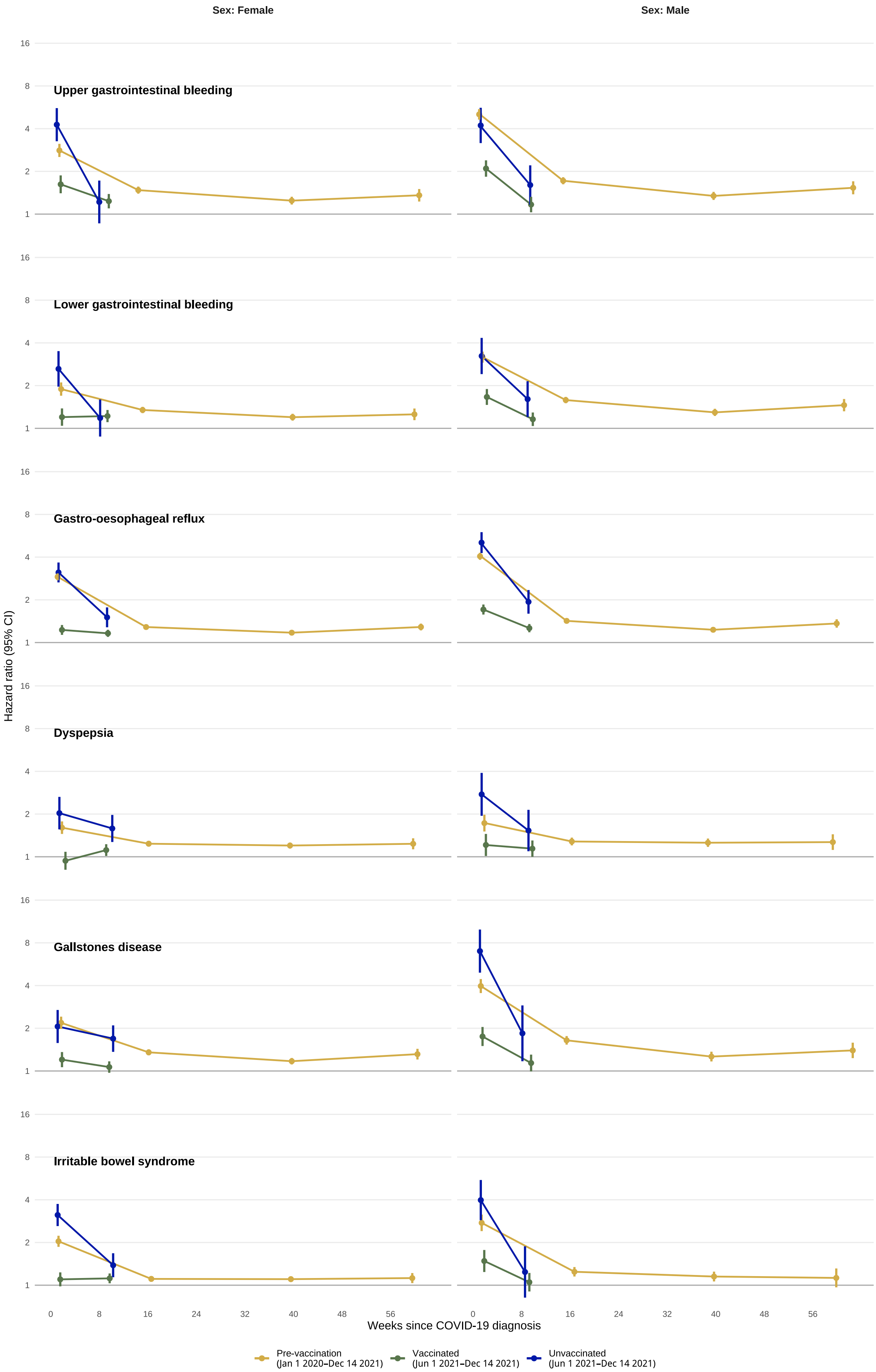

Supplementary Figure 7. Maximally adjusted hazard ratios and 95% CIs comparing the incidence of GI outcome diseases after COVID-19 with the incidence before or without COVID-19, in the pre-vaccination, vaccinated and unvaccinated cohorts, by ethnicity.

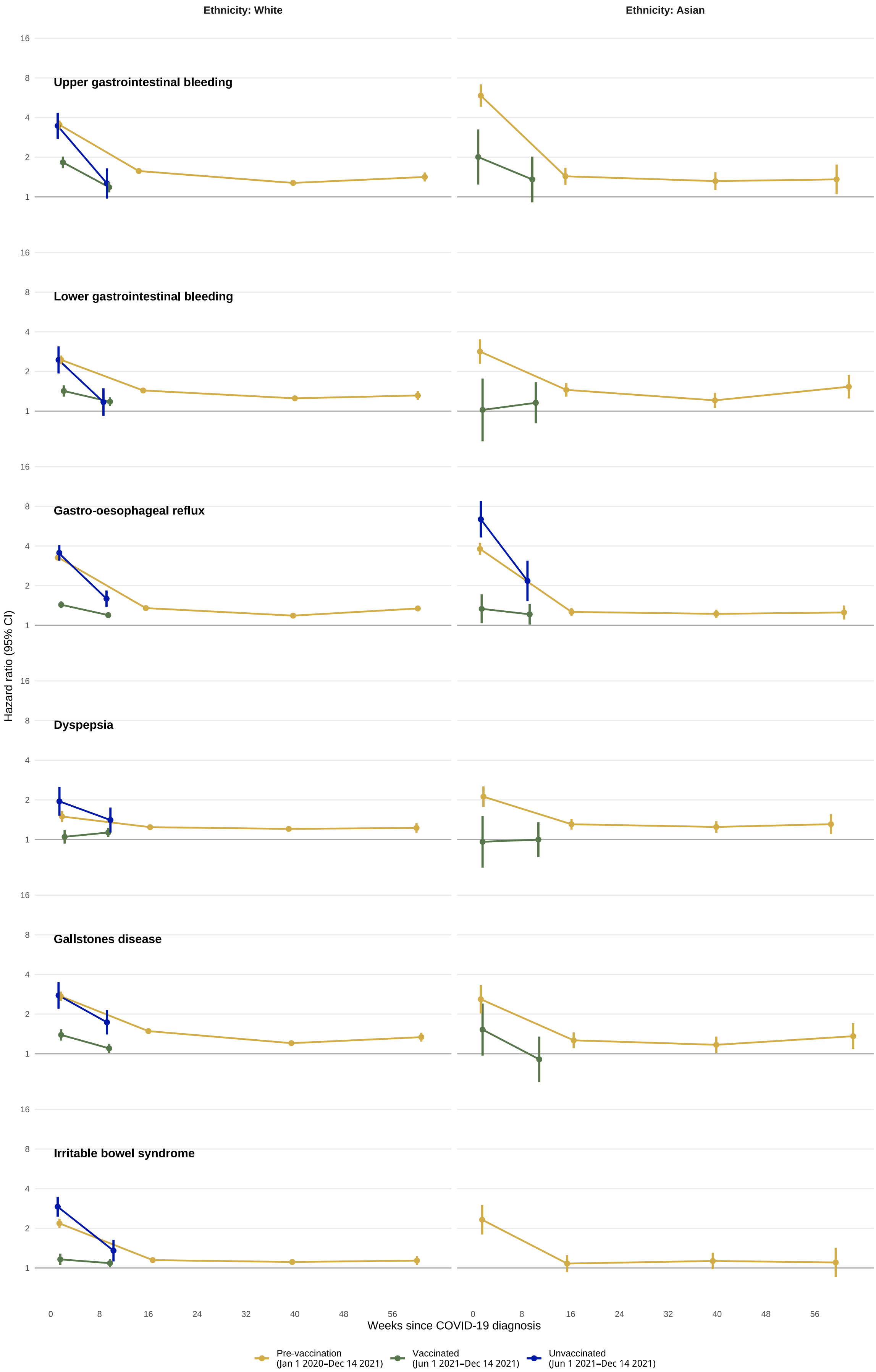

Supplementary Figure 8. Maximally adjusted hazard ratios and 95% CIs comparing the incidence of GI outcome diseases after COVID-19 with the incidence before or without COVID-19, in the pre-vaccination, vaccinated and unvaccinated cohorts, by prior history of gastrointestinal operations.

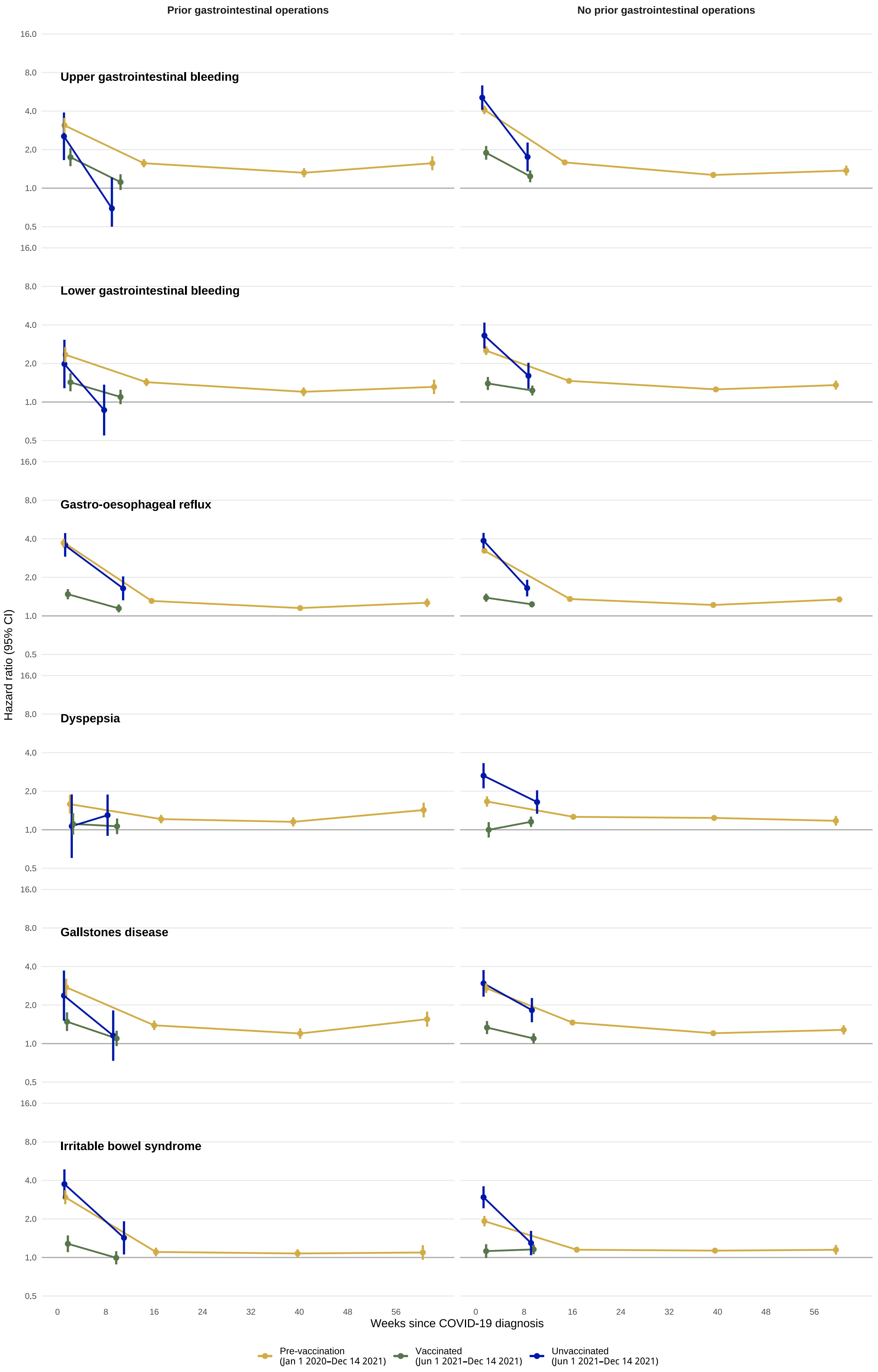

Supplementary Figure 9. Maximally adjusted hazard ratios and 95% CIs comparing the incidence of GI outcome diseases after COVID-19 with the incidence before or without COVID-19, in the pre-vaccination, vaccinated and unvaccinated cohorts, by prior history of gastrointestinal event.

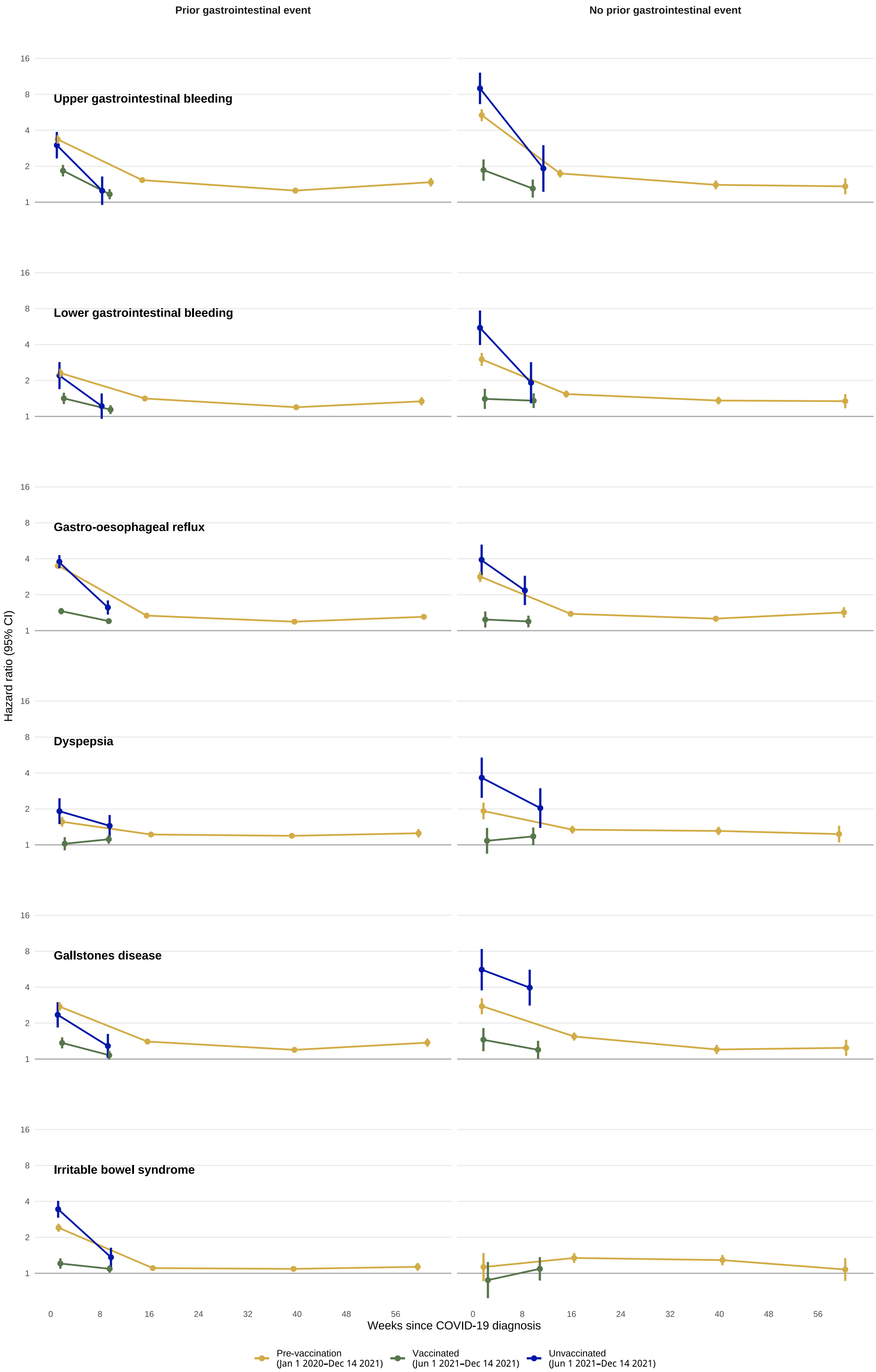

Supplementary Figure 10. Estimated absolute increase in risk of upper gastrointestinal bleeding over time since diagnosis of COVID-19, compared with no COVID-19 diagnosis, in the pre-vaccination, vaccinated and unvaccinated cohorts.

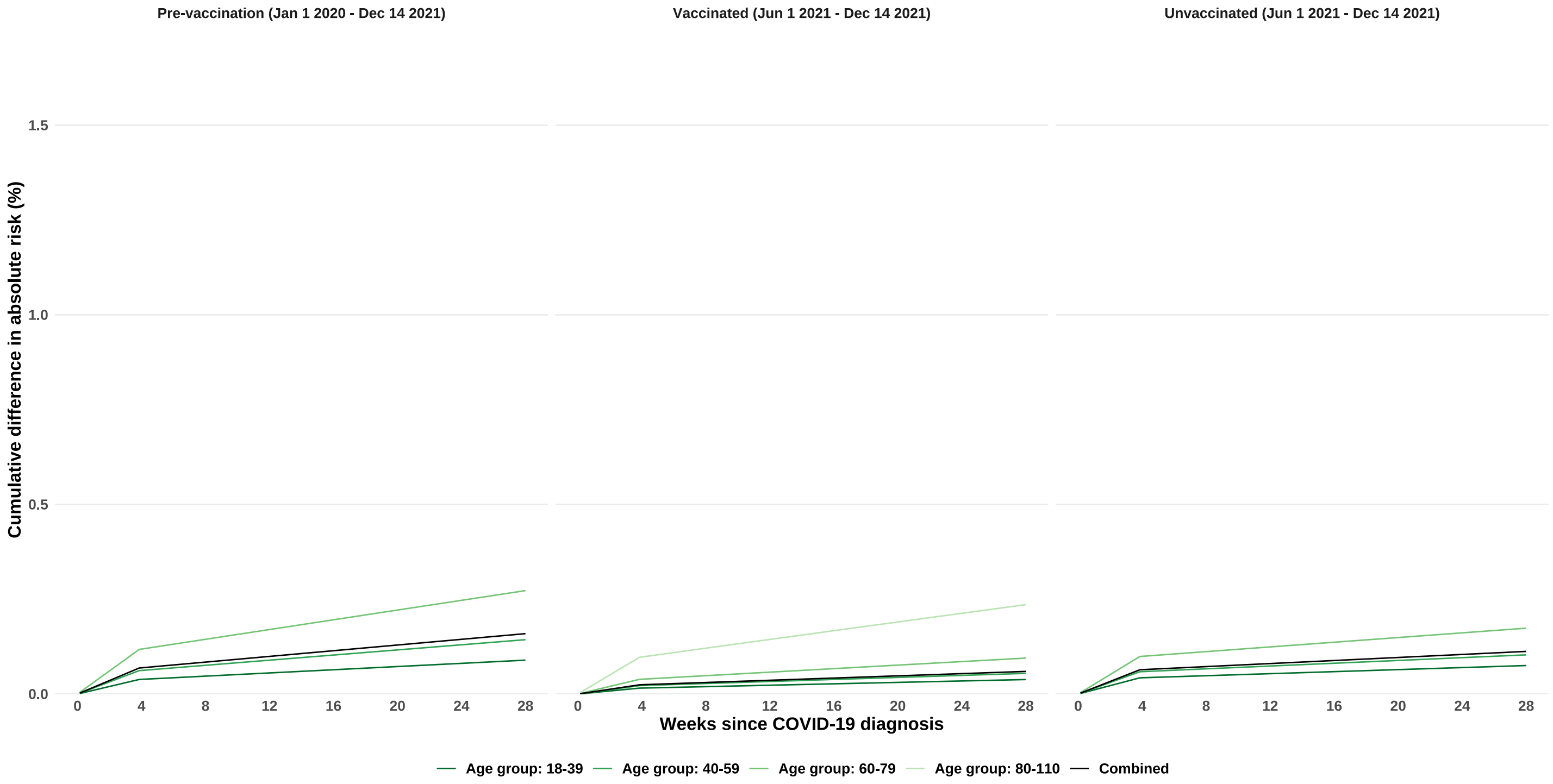

Supplementary Figure 11. Estimated absolute increase in risk of lower gastrointestinal bleeding over time since diagnosis of COVID-19, compared with no COVID-19 diagnosis, in the pre-vaccination, vaccinated and unvaccinated cohorts.

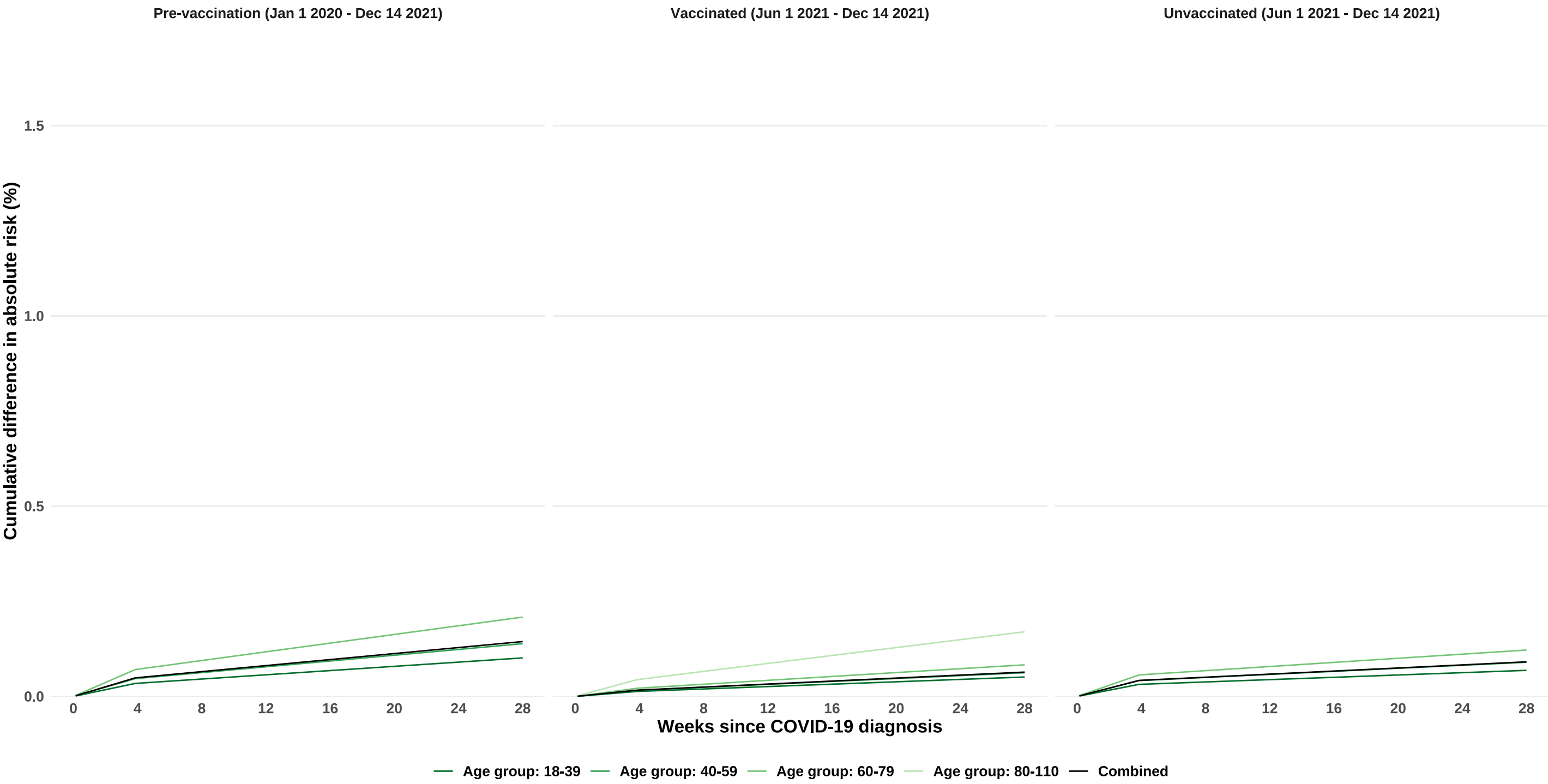

Supplementary Figure 12. Estimated absolute increase in risk of gastro-oesophageal reflux over time since diagnosis of COVID-19, compared with no COVID-19 diagnosis, in the pre-vaccination, vaccinated and unvaccinated cohorts.

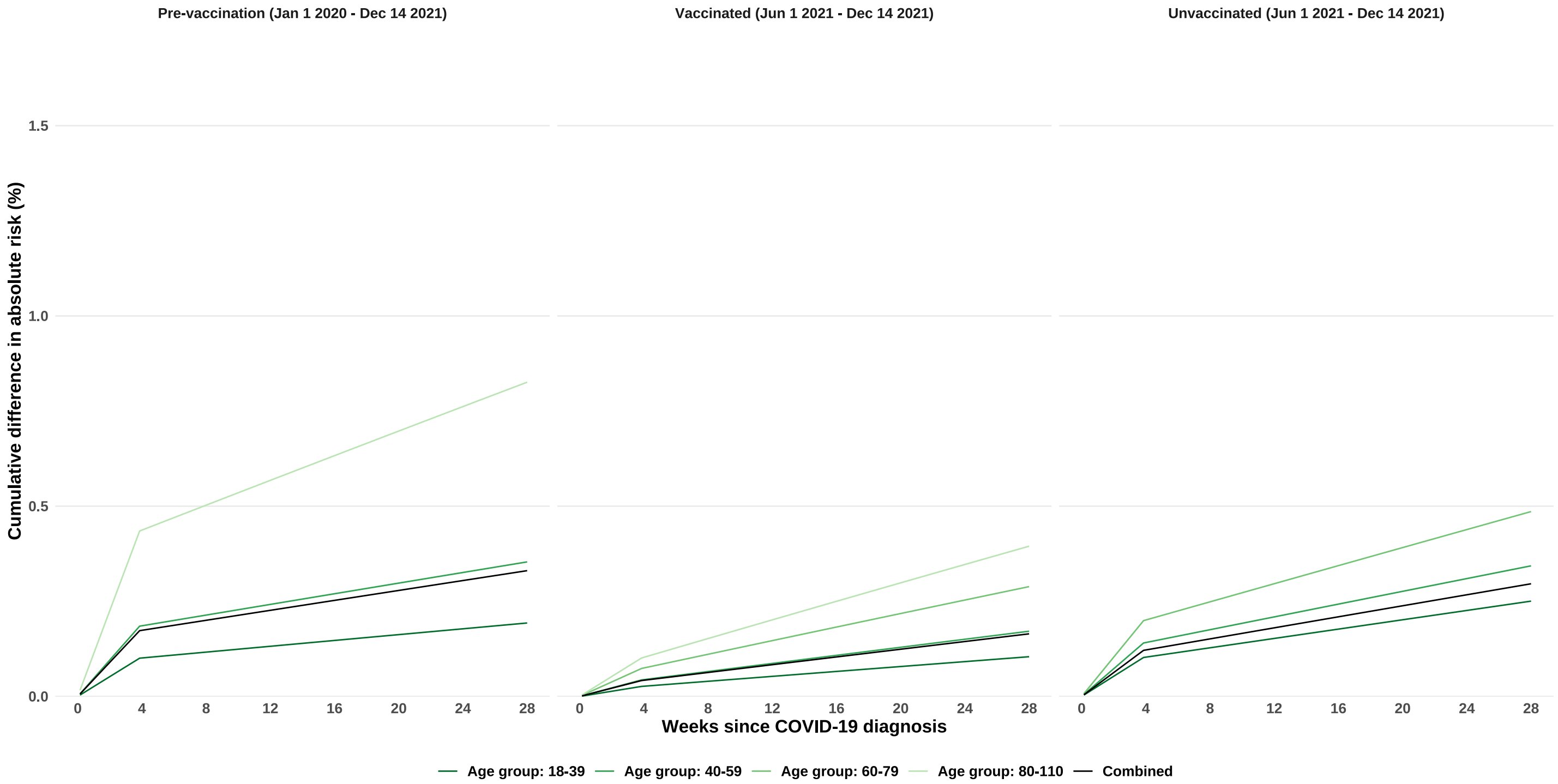

Supplementary Figure 13. Estimated absolute increase in risk of dyspepsia over time since diagnosis of COVID-19, compared with no COVID-19 diagnosis, in the pre-vaccination, vaccinated and unvaccinated cohorts.

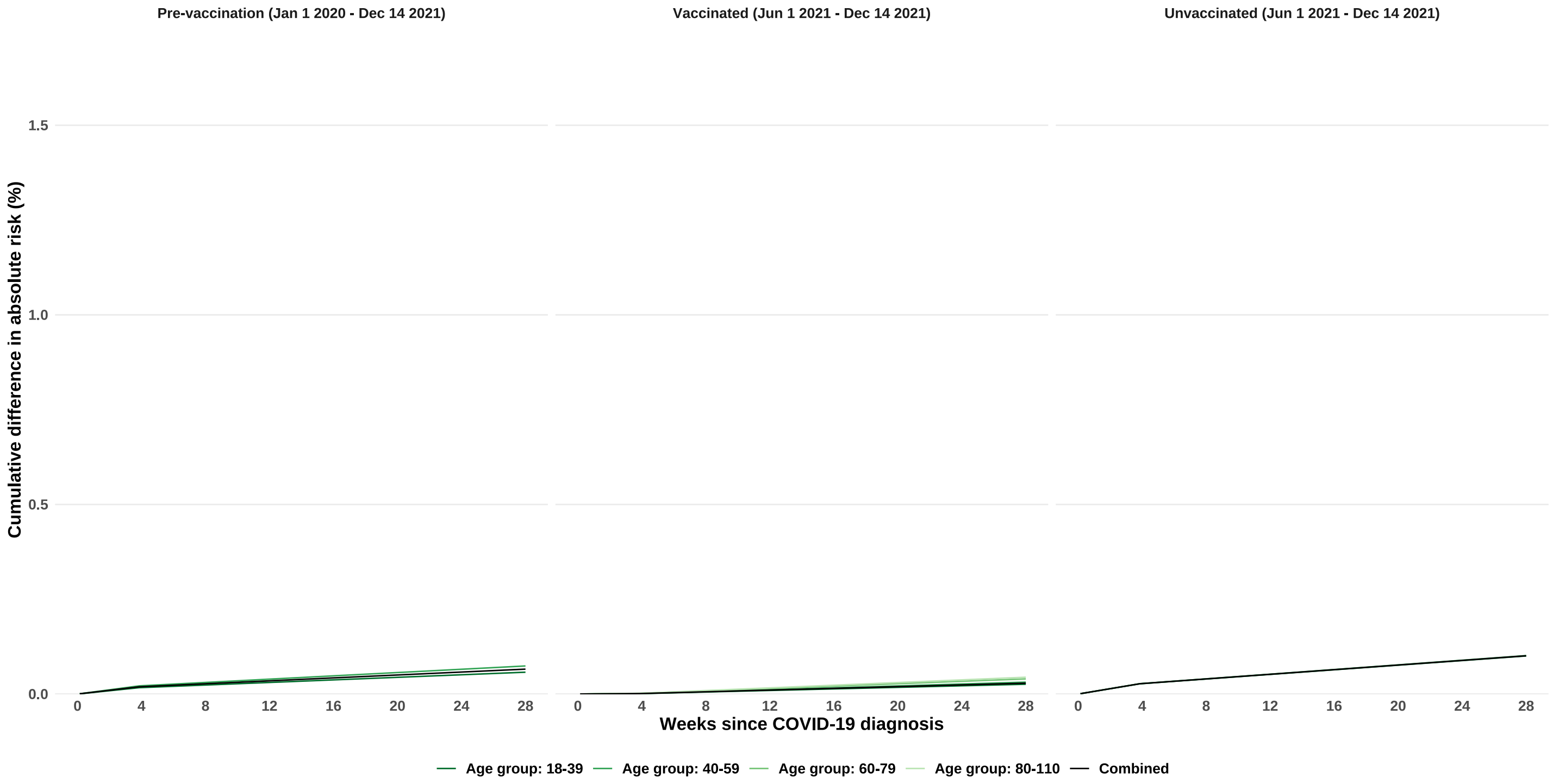

Supplementary Figure 14. Estimated absolute increase in risk of gallstones over time since diagnosis of COVID-19, compared with no COVID-19 diagnosis, in the pre-vaccination, vaccinated and unvaccinated cohorts.

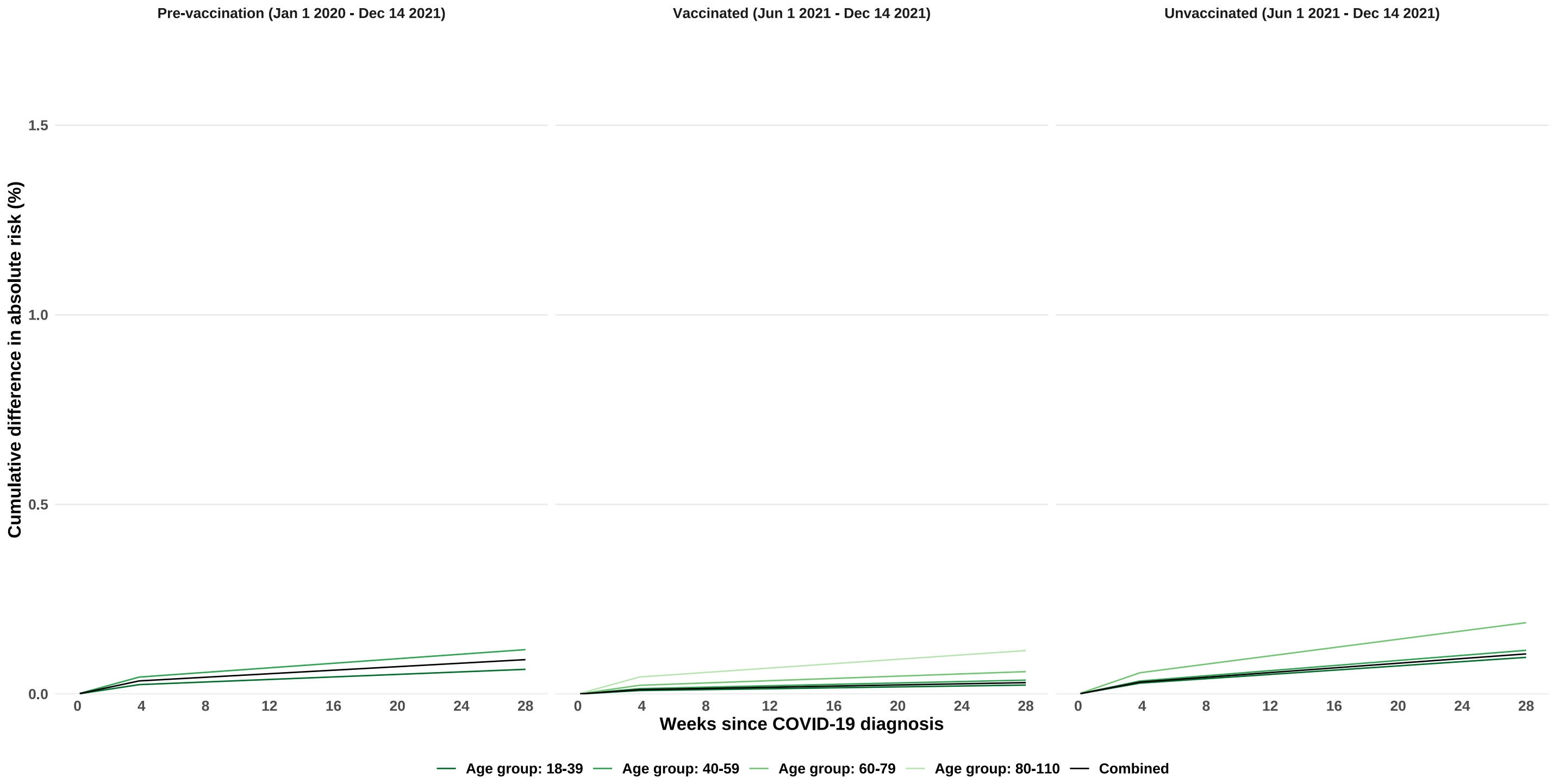

Supplementary Figure 15. Estimated absolute increase in risk of irritable bowel syndrome over time since diagnosis of COVID-19, compared with no COVID-19 diagnosis, in the pre-vaccination, vaccinated and unvaccinated cohorts.

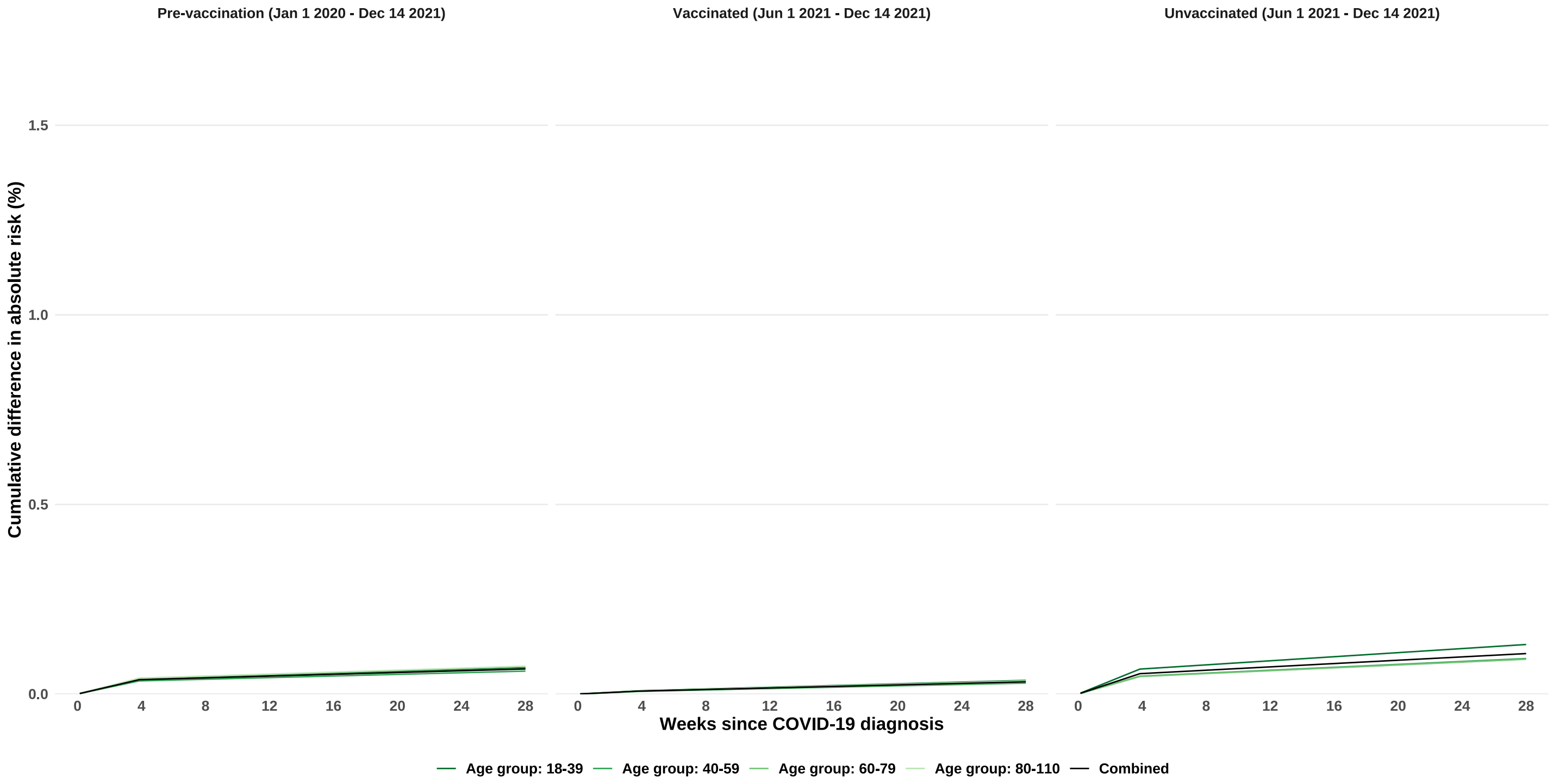

Supplement: Supplementary file 1 [file bmjph-4-4-s001.pdf]
