# Supplementary material for: Impact of COVID-19 on gastrointestinal disease in vaccinated and unvaccinated individuals: a population-based cohort study in England
Source: BMJ Public Health. 2026 Oct 20;4(4):e004709. doi: 10.1136/bmjph-2025-004709 (PMC13691888; doi:10.1136/bmjph-2025-004709)
Supplement: Supplementary file 2 [file bmjph-4-4-s002.docx]

**The impact of COVID-19 on gastrointestinal disease in vaccinated and unvaccinated individuals: a population-based cohort study in England**

[Supplementary Methods	3](#_Toc226999263)

[Quality assurance	3](#_Toc226999264)

[Absolute excess risk	3](#_Toc226999265)

[Information governance	3](#_Toc226999266)

[Supplementary Tables	4](#_Toc226999267)

[Supplementary Table 1. Summary of covariate definitions. *Index of multiple deprivation. ** gastrointestinal	4](#_Toc226999268)

[Supplementary Table 2. Codelists for outcomes, exclusion outcomes, symptoms and Exposure.	5](#_Toc226999269)

[Supplementary Table 3: Summary of cohort characteristics.	6](#_Toc226999270)

[Supplementary Table 4: Age-sex adjusted hazard ratios (95% CI) comparing the incidence of gastrointestinal disease events after COVID-19 with the incidence before or without COVID-19, in the pre-vaccination, vaccinated and unvaccinated cohorts.	9](#_Toc226999271)

[Supplementary Table 5. Adjusted hazard ratios (95% CI) comparing the incidence of gastrointestinal disease events after COVID-19 with the incidence before or without COVID-19, in the pre-vaccination, vaccinated and unvaccinated cohorts, in hospitalised subgroup. Hazard ratios are maximally adjusted.	10](#_Toc226999272)

[Supplementary Table 6. Adjusted hazard ratios (95% CI) comparing the incidence of gastrointestinal disease events after COVID-19 with the incidence before or without COVID-19, in the pre-vaccination, vaccinated and unvaccinated cohorts, in non-hospitalised subgroup. Hazard ratios are maximally adjusted.	11](#_Toc226999273)

[Supplementary Table 7. Adjusted hazard ratios (95% CI) comparing the incidence of gastrointestinal disease events after COVID-19 with the incidence before or without COVID-19, in the pre-vaccination, vaccinated and unvaccinated cohorts, in 18–39 age group. Hazard ratios are maximally adjusted.	12](#_Toc226999274)

[Supplementary Table 8. Adjusted hazard ratios (95% CI) comparing the incidence of gastrointestinal disease events after COVID-19 with the incidence before or without COVID-19, in the pre-vaccination, vaccinated and unvaccinated cohorts, in 40–59 age group. Hazard ratios are maximally adjusted.	13](#_Toc226999275)

[X insufficient events	13](#_Toc226999276)

[Supplementary Table 9. Adjusted hazard ratios (95% CI) comparing the incidence of gastrointestinal disease events after COVID-19 with the incidence before or without COVID-19, in the pre-vaccination, vaccinated and unvaccinated cohorts, in 60–79 age group. Hazard ratios are maximally adjusted.	14](#_Toc226999277)

[X insufficient events	14](#_Toc226999278)

[Supplementary Table 10. Adjusted hazard ratios (95% CI) comparing the incidence of gastrointestinal disease events after COVID-19 with the incidence before or without COVID-19, in the pre-vaccination, vaccinated and unvaccinated cohorts, in 80–110 age group. Hazard ratios are maximally adjusted.	15](#_Toc226999279)

[Supplementary Table 11 Adjusted hazard ratios (95% CI) comparing the incidence of gastrointestinal disease events after COVID-19 with the incidence before or without COVID-19, in the pre-vaccination, vaccinated and unvaccinated cohorts, in White ethnicity. Hazard ratios are maximally adjusted.	16](#_Toc226999280)

[X insufficient events	16](#_Toc226999281)

[Supplementary Table 12: Adjusted hazard ratios (95% CI) comparing the incidence of gastrointestinal disease events after COVID-19 with the incidence before or without COVID-19, in the pre-vaccination, vaccinated and unvaccinated cohorts, in South Asian ethnicity. Hazard ratios are maximally adjusted.	17](#_Toc226999282)

[Supplementary Table 13: Adjusted hazard ratios (95% CI) comparing the incidence of gastrointestinal disease events after COVID-19 with the incidence before or without COVID-19, in the pre-vaccination, vaccinated and unvaccinated cohorts, in Female sex. Hazard ratios are maximally adjusted.	18](#_Toc226999283)

[Supplementary Table 14: Adjusted hazard ratios (95% CI) comparing the incidence of gastrointestinal disease events after COVID-19 with the incidence before or without COVID-19, in the pre-vaccination, vaccinated and unvaccinated cohorts, in Male sex. Hazard ratios are maximally adjusted.	19](#_Toc226999284)

[Supplementary Table 15: Adjusted hazard ratios (95% CI) comparing the incidence of gastrointestinal disease events after COVID-19 with the incidence before or without COVID-19, in the pre-vaccination, vaccinated and unvaccinated cohorts, in individuals with prior history of gastrointestinal diseases. Hazard ratios are maximally adjusted.	20](#_Toc226999285)

[Supplementary Table 16: Adjusted hazard ratios (95% CI) comparing the incidence of gastrointestinal disease events after COVID-19 with the incidence before or without COVID-19, in the pre-vaccination, vaccinated and unvaccinated cohorts, in individuals without prior history of gastrointestinal diseases. Hazard ratios are maximally adjusted.	21](#_Toc226999286)

[Supplementary Table 17: Adjusted hazard ratios (95% CI) comparing the incidence of gastrointestinal disease events after COVID-19 with the incidence before or without COVID-19, in the pre-vaccination, vaccinated and unvaccinated cohorts, in individuals with prior history of gastrointestinal operations. Hazard ratios are maximally adjusted.	22](#_Toc226999287)

[Supplementary Table 18: Adjusted hazard ratios (95% CI) comparing the incidence of gastrointestinal disease events after COVID-19 with the incidence before or without COVID-19, in the pre-vaccination, vaccinated and unvaccinated cohorts, in individuals without prior history of gastrointestinal operations. Hazard ratios are maximally adjusted.	23](#_Toc226999288)

[Supplementary Table 19. Estimated number of excess diseases for each outcome per 100,000 COVID-19 diagnoses, in the pre-vaccination, vaccinated and unvaccinated cohorts. Increases in risks were estimated within age groups, and the estimated overall increase in risk is the average of these, weighted according to the proportions in each age group in the pre-vaccination cohort.	24](#_Toc226999289)

[The RECORD checklist.	25](#_Toc226999290)

# Supplementary Methods

## Quality assurance

We applied the following quality assurance rules:

1. Remove individuals who are missing year of birth
2. Remove individuals whose year of birth is after their year of death
3. Remove individuals whose year of birth is after today
4. Remove individuals whose date of death is after today
5. Remove men whose records contain pregnancy and/or birth codes
6. Remove men whose records contain HRT or COCP medication codes
7. Remove women whose records contain prostate cancer codes

## Absolute excess risk

The absolute excess risk analysis was performed for each outcome in each cohort using the hazard ratio from the main analysis. To compare the outcomes across the cohorts, each of which have different lengths of follow-up, we calculated the absolute excess risk at 28 weeks.  We accounted for age in the analysis by using event counts and person-days from four age groups: 18–39, 40–59, 60–79, and 80–110 years. For each group, we calculated the average daily incidence of the outcome in the unexposed and the cumulative risk over time. We then used the relevant hazard ratio for that day (e.g., days 0 to 27 will have the coefficient for the term ‘days0_28’, while days 28 to 196 will have the coefficient for the term ‘days28_197’) to predict the expected cumulative survival in the exposed. Finally, we calculated the daily excess risk as the difference in cumulative survival for the unexposed and the expected cumulative survival in the exposed. The overall absolute excess risk was estimated using a weighted sum of the excess risks in each group, weighted by the proportions of individuals in age strata in the pre-vaccine availability cohort.

## Information governance

NHS England is the data controller of the NHS England OpenSAFELY COVID-19 Service; TPP is the data processor; all study authors using OpenSAFELY have the approval of NHS England. This implementation of OpenSAFELY is hosted within the TPP environment which is accredited to the ISO 27001 information security standard and is NHS IG Toolkit compliant.

Patient data have been pseudonymised for analysis and linkage using industry standard cryptographic hashing techniques; all pseudonymised datasets transmitted for linkage onto OpenSAFELY are encrypted; access to the NHS England OpenSAFELY COVID-19 service is via a virtual private network (VPN) connection; the researchers hold contracts with NHS England and only access the platform to initiate database queries and statistical models; all database activity is logged; only aggregate statistical outputs leave the platform environment following best practice for anonymisation of results such as statistical disclosure control for low cell counts.

The service adheres to the obligations of the UK General Data Protection Regulation (UK GDPR) and the Data Protection Act 2018. The service previously operated under notices initially issued in February 2020 by the Secretary of State under Regulation 3(4) of the Health Service (Control of Patient Information) Regulations 2002 (COPI Regulations), which required organisations to process confidential patient information for COVID-19 purposes; this set aside the requirement for patient consent. As of 1 July 2023, the Secretary of State has requested that NHS England continue to operate the Service under the COVID-19 Directions 2020. In some cases of data sharing, the common law duty of confidence is met using, for example, patient consent or support from the Health Research Authority Confidentiality Advisory Group.

Taken together, these provide the legal bases to link patient datasets using the service. GP practices, which provide access to the primary care data, are required to share relevant health information to support the public health response to the pandemic and have been informed of how the service operates.

This study was approved by the Health Research Authority [REC reference 22/PR/0095] and by the University of Bristol's Faculty of Health Sciences Ethics Committee [reference 117269].

# Supplementary Tables

## Supplementary Table 1: Summary of covariate definitions. *Index of multiple deprivation. ** gastrointestinal

| Confounder | Type | Definition | Data source/Codelists |
| --- | --- | --- | --- |
| Sex | Categorical | Male, Female | [Primary care](https://docs.opensafely.org/legacy/study-def-variables/#:~:text=sex(return_expectations%3DNone)) |
| Age | Continuous | Modelled as age in years using a restricted cubic spline with 3 knots at the 10^th^, 50^th^ and 90^th^ percentiles | [All](https://docs.opensafely.org/legacy/study-def-variables/#:~:text=0.49%2C%20%22F%22%3A%200.51%7D%7D%2C%0A%20%20%20%20%7D%0A)-,age_as_of,-(reference_date%2C)(OpenSafely) |
| Ethnicity | Categorical | 1: White  2: Mixed  3: South Asian  4: Black  5: Other  Missing | [Primary care](https://www.opencodelists.org/codelist/opensafely/ethnicity/2020-04-27/), [primis](https://www.opencodelists.org/codelist/primis-covid19-vacc-uptake/eth2001/v1/), Sus (OpenSafely) |
| Deprivation | Categorical | Area-level socioeconomic deprivation measured using IMD* 2019 quintiles (1 = most deprived, 5 = least deprived) based on residential area level at baseline. | Index of Multiple Deprivation (OpenSafely) |
| Region | Categorical | East of England  London  Midlands  North East and Yorkshire  North West  South East  South West | [Primary care](https://docs.opensafely.org/legacy/study-def-variables/#:~:text=registered_practice_as_of(date%2C%20returning%3DNone%2C%20return_expectations%3DNone))(OpenSafely) |
| Consultation rate | Continuous | Number of primary care contacts in the year prior to pandemic start (2019). | [Primary care](https://docs.opensafely.org/legacy/study-def-variables/#:~:text=with_gp_consultations(on_or_before%3DNone%2C%20on_or_after%3DNone%2C%20between%3DNone%2C)(Opensafely) |
| Smoking status | Categorical | E: Ever smoker  M: Missing  N: Never smoker  S: Current smoker | [Primary care](https://www.opencodelists.org/codelist/opensafely/smoking-clear/2020-04-29/) |
| Obesity | Binary | 1: coded diagnosis of obesity recorded in primary care or secondary care at any time prior to baseline; 0: otherwise | [Primary care](https://github.com/opensafely/post-covid-vaccinated/blob/79f1d781dafbc3bbb1980f9675e2bc27fdfdcb15/codelists/user-elsie_horne-bmi_obesity_snomed.csv) [ICD10](https://github.com/opensafely/post-covid-vaccinated/blob/79f1d781dafbc3bbb1980f9675e2bc27fdfdcb15/codelists/user-elsie_horne-bmi_obesity_icd10.csv) |
| Healthcare worker | Binary | 1: if healthcare worker; 0: otherwise | [NHS E COVID-19 data store](https://docs.opensafely.org/legacy/study-def-variables/#cohortextractor.patients.with_healthcare_worker_flag_on_covid_vaccine_record):~:text=NHS%20England%20COVID%2D19%20data%20store%2C) |
| Care home resident | Binary | 1: if care home resident; 0: otherwise | [CQC database](https://docs.opensafely.org/legacy/study-def-variables/#cohortextractor.patients.with_healthcare_worker_flag_on_covid_vaccine_record):~:text=care_home_status_as_of(date,using%2Dcqc%2Ddata) |
| Alcohol above limits | Binary | 1: exceeds limits 5 years prior to baseline; 0: within limits | [Primary care](https://www.opencodelists.org/codelist/bristol/alcohol-snomed/261cd28e/#full-list) [CTV3](https://www.opencodelists.org/codelist/opensafely/hazardous-alcohol-drinking/6364474b/#full-list) |
| Cholelithiasis | Binary | 1: diagnosis present at any time prior to baseline; 0: otherwise | [Primary care](https://www.opencodelists.org/codelist/bristol/cholelisthiasis-snomed/1fa4cbc4/#full-list) [ICD10](https://www.opencodelists.org/codelist/bristol/cholelisthiasis-icd10/0695588f/#full-list) |
| H pylori infection | Binary | 1: diagnosis present at any time prior to baseline; 0: otherwise | [Primary care](https://www.opencodelists.org/codelist/bristol/h-pylori-infection-snomed/481e0b2e/#tree) [ICD10](https://www.opencodelists.org/codelist/bristol/cholelisthiasis-icd10/0695588f/#full-list) |
| NSAIDS medication | Binary | 1: ≥1 prescription issued within two years prior to baseline; 0: otherwise | [Pseudo BNF](https://www.opencodelists.org/codelist/bristol/nsaids/05cea500/#tree) |
| Aspirin medication | Binary | 1: ≥1 prescription issued within two years prior to baseline; 0: otherwise | [Pseudo BNF](https://www.opencodelists.org/codelist/bristol/aspirin/6cbf31ca/#tree) |
| Anticoagulants medications | Binary | 1: ≥1 prescription issued within two years prior to baseline; 0: otherwise | [Pseudo BNF](https://www.opencodelists.org/codelist/bristol/anticoagulants/1099a4d8/#tree) |
| Antidepressants | Binary | 1: ≥1 prescription issued within two years prior to baseline; 0: otherwise | [Pseudo BNF](https://www.opencodelists.org/codelist/bristol/antidepressants-bnf/7a3cf198/#tree) |
| Prior GI** symptoms and diseases | Binary | 1: if diagnosis at any time prior to baseline; 0: otherwise | See Supplementary Table 2. |
| Previous gastrointestinal operation | Binary | 1: if existing operation at any time prior to baseline; 0: otherwise | [OPCS4](https://www.opencodelists.org/codelist/bristol/gi-operations-opcs4/6f5f1fc6/#full-list) |
| Hypercalcemia | Binary | 1: diagnosis present at any time prior to baseline, 0: otherwise | [Primary care](https://www.opencodelists.org/codelist/bristol/hypercalcemia-snomed/735cdc88/#full-list) |
| Hypertriglyceridemia | Binary | 1: diagnosis present at any time prior to baseline, 0: otherwise | [Primary care](https://www.opencodelists.org/codelist/bristol/hypertriglyceridemia-snomed/282e82e9/) |
| HDL level | Continuous | Most recent record 5 years prior to baseline | [Primary care](https://www.opencodelists.org/codelist/bristol/hdl-cholesterol/64775990/#full-list) |
| Systolic blood pressure | Continuous | Most recent record 5 years prior to baseline | CTV3 ["2469."] |

Study design, including selection of outcomes and potential confounders, was informed by prior literature review, clinical input and data availability.

## Supplementary Table 2: Codelists for outcomes, exclusion outcomes, symptoms and Exposure.

| **Outcomes** | **Primary care** | **Secondary care** |
| --- | --- | --- |
| Upper gastrointestinal bleeding | [Primary care](https://www.opencodelists.org/codelist/bristol/upper-gi-bleeding-snomed/2c490609/#full-list) | [ICD10](https://www.opencodelists.org/codelist/bristol/upper-gi-bleeding-icd10/77775fa8/#full-list) |
| Lower gastrointestinal bleeding | [Primary care](https://www.opencodelists.org/codelist/bristol/lower-gi-bleeding-snomed/31ae85f5/#full-list) | [ICD10](https://www.opencodelists.org/codelist/bristol/lower-gi-bleeding-icd10/6e986c37/#full-list) |
| Acute pancreatitis | [Primary care](https://www.opencodelists.org/codelist/bristol/acute-pancreatitis-snomed/075c0c1e/#full-list) | [ICD10](https://www.opencodelists.org/codelist/bristol/acute-pancreatitis-icd10/67d4921e/) |
| Appendicitis | [Primary care](https://www.opencodelists.org/codelist/bristol/appendicitis-snomed/729628f9/#tree) | [ICD10](https://www.opencodelists.org/codelist/bristol/appendicitis-icd10/0e61c522/) |
| Gastro-oesophageal reflux | [Primary care](https://www.opencodelists.org/codelist/bristol/gastro-oesophageal-reflux-disease-snomed/11148518/) | [ICD10](https://www.opencodelists.org/codelist/bristol/gastro-oesophageal-reflux-disease-icd10/35b5abb4/) |
| Dyspepsia | [Primary care](https://www.opencodelists.org/codelist/bristol/dyspepsia-snomed/4193326a/#full-list) | [ICD10](https://www.opencodelists.org/codelist/bristol/dyspesia-icd10/3b66feef/#full-list) |
| Peptic ulcer | [Primary care](https://www.opencodelists.org/codelist/bristol/peptic-ulcer-snomed/3e06eceb/#full-list) | [ICD10](https://www.opencodelists.org/codelist/bristol/peptic-ulcer-icd10/24f779b6/#full-list) |
| Non-alcoholic steatohepatitis | [Primary care](https://www.opencodelists.org/codelist/bristol/nonalcoholic-steatohepatitis-snomed/46f8b256/#tree) | [ICD10](https://www.opencodelists.org/codelist/bristol/nonalcoholic-steatohepatitis-icd10/22578bba/#full-list) |
| Gallstones | [Primary care](https://www.opencodelists.org/codelist/bristol/gallstones-disease-snomed/1300917f/#tree) | [ICD10](https://www.opencodelists.org/codelist/bristol/gallstone-disease-icd10/6cd203c4/#tree) |
| Irritable bowel syndrome | [Primary care](https://www.opencodelists.org/codelist/bristol/ibs-snomed/5d556595/#full-list) | [ICD10](https://www.opencodelists.org/codelist/bristol/ibs-icd10/220bb86b/) |
| **Exclusion outcomes** | | |
| Crohn’s disease | [Primary care](https://www.opencodelists.org/codelist/bristol/crohn-snomed/7025d355/#full-list) | [ICD10](https://www.opencodelists.org/codelist/bristol/crohn-icd10/57166020/#full-list) |
| Coeliac disease | [Primary care](https://www.opencodelists.org/codelist/bristol/coeliac-disease-snomed/6e00c599/#full-list) | [ICD10](https://www.opencodelists.org/codelist/bristol/coeliac-disease-icd10/3dc48299/#full-list) |
| Cirrhosis | [Primary care](https://www.opencodelists.org/codelist/bristol/cirrhosis-snomed/3b542cf5/#full-list) | [ICD10](https://www.opencodelists.org/codelist/bristol/cirrhosis-icd10/2244b9c0/#full-list) |
| **Symptoms** | | |
| Diarrhoea | [Primary care](https://www.opencodelists.org/codelist/bristol/diarrhoea-snomed/7e7d18ac/) | [ICD10](https://www.opencodelists.org/codelist/bristol/diarrhoea-icd10/6d85e559/) |
| Nausea | [Primary care](https://www.opencodelists.org/codelist/bristol/nausea-snomed/62bae581/) | [ICD10](https://www.opencodelists.org/codelist/bristol/nausea-icd10/09c2f8c5/) |
| Vomiting | [Primary care](https://www.opencodelists.org/codelist/bristol/vomiting-snomed/791798c0/) | [ICD10](https://www.opencodelists.org/codelist/bristol/vomiting-icd10/2430c627/) |
| Abdominal pain and discomfort | [Primary care](https://www.opencodelists.org/codelist/bristol/abdominal-paindiscomfort-snomed/70b3858f/) | [ICD10](https://www.opencodelists.org/codelist/bristol/abdominal-paindiscomfort-icd10/03e29899/) |
| Bowel ischaemia | [Primary care](https://www.opencodelists.org/codelist/bristol/bowel-ischaemia-snomed/6140505d/) | [ICD10](https://www.opencodelists.org/codelist/bristol/bowel-ischaemia-icd10/38b43ef9/) |
| Intestinal obstruction | [Primary care](ttps://www.opencodelists.org/codelist/bristol/intestinal-obstruction-snomed/2b367f2b/) | [ICD10](https://www.opencodelists.org/codelist/bristol/intestinal-obstruction-icd10/3ab31d5a/) |
| Belching | [Primary care](https://www.opencodelists.org/codelist/bristol/belching-snomed/0baf052c/) | [ICD10](https://www.opencodelists.org/codelist/bristol/belching-icd10/51c3b22e/) |
| Abdominal distension | [Primary care](https://www.opencodelists.org/codelist/bristol/abdominal-distension-snomed/3e949f25/) | [ICD10](https://www.opencodelists.org/codelist/bristol/abdominal-distension-icd10/1cf20bce/) |
| Bloody stools | [Primary care](https://www.opencodelists.org/codelist/bristol/bloody-stools-snomed/70ff58de/) | [ICD10](https://www.opencodelists.org/codelist/bristol/bloody-stools-icd10/6ad32563/) |
| **Exposure** |  |  |
| COVID-19 diagnosis | [Positive tests](%22https:/www.opencodelists.org/codelist/opensafely/covid-identification-in-primary-care-probable-covid-positive-test/2020-07-16), [diagnosis](https://www.opencodelists.org/codelist/opensafely/covid-identification-in-primary-care-probable-covid-clinical-code/2020-07-16/), [sequalae](https://www.opencodelists.org/codelist/opensafely/covid-identification-in-primary-care-probable-covid-sequelae/2020-07-16/) | [ICD10](https://www.opencodelists.org/codelist/user/RochelleKnight/confirmed-hospitalised-covid-19/1f0d2526/) |

Study design, including selection of outcomes and potential confounders, was informed by prior literature review, clinical input and data availability.

## Supplementary Table 3: Summary of cohort characteristics.

| Characteristic | Pre-vaccination cohort | Vaccinated cohort | Unvaccinated cohort |
| --- | --- | --- | --- |
| Start date | 01/01/2020, which is the approximate start date of the pandemic in the UK. | 01/06/2021, which is the date that the delta variant was thought to be ubiquitous in England. | 01/06/2021, which is the date that the delta variant was thought to be ubiquitous in England. |
| End date - exposure | 18/06/2021, which is the date when the Joint Committee for Vaccination and Immunisation (JCVI) phase 2, group 12 (all adults aged 18 years and older) become eligible for a COVID-19 vaccination. | 14/12/2021, which is the day that the UK Health Security Agency stated that over half of English cases they sampled have S Gene Target Failure, meaning they were likely Omicron.* | 14/12/2021, which is the day that the UK Health Security Agency stated that over half of English cases they sampled have S Gene Target Failure, meaning they were likely Omicron.* |
| End date - outcome | 14/12/2021, which is the day that the UK Health Security Agency stated that over half of English cases they sampled have S Gene Target Failure, meaning they were likely Omicron.* | 14/12/2021, which is the day that the UK Health Security Agency stated that over half of English cases they sampled have S Gene Target Failure, meaning they were likely Omicron.* | 14/12/2021, which is the day that the UK Health Security Agency stated that over half of English cases they sampled have S Gene Target Failure, meaning they were likely Omicron.* |
| Exclusion criteria | Patients will be excluded if they meet any of the following criteria:  COVID-19 diagnosis recorded prior to their index date | Patients will be excluded if they meet any of the following criteria:  COVID-19 diagnosis recorded prior to their index date  They do not have a record of two vaccination doses prior to the study end date  They received a vaccination prior to 08-12-2020 (i.e., the start of the vaccination program)  They received a second dose vaccination before their first dose vaccination  They received a second dose vaccination less than three weeks after their first dose  They received mixed vaccine products before 07-05-2021 | Patients will be excluded if they meet any of the following criteria:  COVID-19 diagnosis recorded prior to their index date  They have a record of one or more vaccination doses prior to their index date  They could not be assigned to a vaccination group as defined by the Joint Committee on Vaccination and Immunisation (JCVI) |
| Index date (time 0) | Study start date. | Index date is the latest of the following dates (i.e., an individual’s index date): Two weeks after their second vaccination; Study start date. | Index date is the latest of the following dates (i.e., an individual’s index date):  12 weeks after they became eligible for vaccination; Study start date. |
| Follow-up end for exposure | Follow-up will end at the earliest of the following dates: Death; Outcome event; Study end date exposure; Deregistration date; Vaccination; Date when eligible for vaccination according to JCVI priority groupings. | Follow-up will end at the earliest of the following dates: Death; Outcome event; Study end date exposure; Deregistration date. | Follow-up will end at the earliest of the following dates: Death; Outcome event; Study end date exposure; Deregistration date; Vaccination. |
| Follow-up end for outcomes | Follow-up will end at the earliest of the following dates: Death; Outcome event; Study end date outcome; Deregistration date. | Follow-up will end at the earliest of the following dates: Death; Outcome event; Study end date outcome; Deregistration date. | Follow-up will end at the earliest of the following dates: Death; Outcome event; Study end date outcome; Deregistration date. |
| Cox regression time periods | [0,28), [28,197), [197, 365), [365,714) | [0,28), [28,197) | [0,28), [28,197) |
| * UK Health Security Agency. (2021). *Omicron daily overview: 17 December 2021*. Retrieved 26 April 2024, from <https://assets.publishing.service.gov.uk/government/uploads/system/uploads/attachment_data/file/1042100/20211217_OS_Daily_Omicron_Overview.pdf> | | | |

## Supplementary Table 4: Age-sex adjusted hazard ratios (95% CI) comparing the incidence of gastrointestinal disease events after COVID-19 with the incidence before or without COVID-19, in the pre-vaccination, vaccinated and unvaccinated cohorts.

X insufficient events
Study design, including selection of outcomes and potential confounders, was informed by prior literature review, clinical input and data availability.

| Outcome | Time since COVID-19 | Pre-vaccination cohort | Vaccinated cohort | Unvaccinated cohort |
| --- | --- | --- | --- | --- |
| Upper gastrointestinal bleeding | Day 0 | 202 (192-212) | 55.3 (50.6-60.4) | 138 (114-166) |
|  | Weeks 1-4 | 4.44 (4.16-4.73) | 1.95 (1.77-2.15) | 5.65 (4.66-6.85) |
|  | Weeks 5-28 | 1.79 (1.72-1.86) | 1.30 (1.20-1.42) | 1.92 (1.52-2.43) |
|  | Weeks 29-52 | 1.45 (1.39-1.52) | - | - |
|  | Weeks 53-102 | 1.74 (1.62-1.87) | - | - |
| Lower gastrointestinal bleeding | Day 0 | 104 (98.3-110) | 31.3 (28.4-34.5) | 74.6 (59.9-92.9) |
|  | Weeks 1-4 | 2.77 (2.59-2.97) | 1.50 (1.36-1.65) | 3.86 (3.14-4.73) |
|  | Weeks 5-28 | 1.63 (1.58-1.69) | 1.30 (1.20-1.39) | 1.92 (1.56-2.36) |
|  | Weeks 29-52 | 1.40 (1.35-1.46) | - | - |
|  | Weeks 53-102 | 1.61 (1.50-1.72) | - | - |
| Acute pancreatitis | Day 0 | 137 (120-157) | 44.3 (35.9-54.6) | 72.0 (45.5-114) |
|  | Weeks 1-4 | 3.21 (2.71-3.81) | 1.78 (1.43-2.22) | 4.02 (2.67-6.06) |
|  | Weeks 5-28 | 1.50 (1.36-1.66) | 1.21 (1.00-1.47) | 1.82 (1.16-2.86) |
|  | Weeks 29-52 | 1.38 (1.24-1.53) | - | - |
|  | Weeks 53-102 | 1.97 (1.68-2.32) | - | - |
| Appendicitis | Day 0 | 53.3 (44.6-63.8) | 18.1 (13.0-25.0) | 37.3 (19.5-71.5) |
|  | Weeks 1-4 | 1.89 (1.57-2.28) | 1.64 (1.31-2.06) | 2.96 (1.80-4.87) |
|  | Weeks 5-28 | 1.30 (1.19-1.42) | 1.36 (1.13-1.64) | 2.25 (1.43-3.54) |
|  | Weeks 29-52 | 1.25 (1.14-1.37) | - | - |
|  | Weeks 53-102 | 1.34 (1.11-1.63) | - | - |
| Gastro-oesophageal reflux | Day 0 | 85.6 (82.1-89.3) | 19.7 (18.3-21.2) | 79.2 (68.8-91.3) |
|  | Weeks 1-4 | 3.91 (3.76-4.07) | 1.52 (1.44-1.61) | 5.46 (4.86-6.13) |
|  | Weeks 5-28 | 1.57 (1.53-1.60) | 1.33 (1.27-1.39) | 2.54 (2.25-2.87) |
|  | Weeks 29-52 | 1.41 (1.37-1.44) | - | - |
|  | Weeks 53-102 | 1.64 (1.57-1.71) | - | - |
| Dyspepsia | Day 0 | 39.2 (36.1-42.6) | 8.14 (6.75-9.83) | 40.8 (31.3-53.1) |
|  | Weeks 1-4 | 1.92 (1.77-2.08) | 1.09 (0.97-1.22) | 3.01 (2.44-3.71) |
|  | Weeks 5-28 | 1.45 (1.40-1.51) | 1.23 (1.14-1.32) | 2.23 (1.86-2.68) |
|  | Weeks 29-52 | 1.43 (1.37-1.48) | - | - |
|  | Weeks 53-102 | 1.55 (1.44-1.67) | - | - |
| Peptic ulcer | Day 0 | 69.1 (57.7-82.8) | 16.2 (11.4-23.1) | 43.9 (20.2-95.4) |
|  | Weeks 1-4 | 4.87 (4.17-5.67) | 2.26 (1.83-2.80) | 3.80 (2.13-6.76) |
|  | Weeks 5-28 | 1.89 (1.72-2.08) | 1.27 (1.04-1.56) | 2.62 (1.57-4.38) |
|  | Weeks 29-52 | 1.42 (1.28-1.59) | - | - |
|  | Weeks 53-102 | 1.50 (1.24-1.81) | - | - |
| Non-alcoholic steatohepatitis | Day 0 | 191 (142-257) | 29.5 (16.2-53.7) | X |
|  | Weeks 1-4 | 6.18 (4.49-8.50) | 2.47 (1.60-3.82) | X |
|  | Weeks 5-28 | 2.25 (1.85-2.73) | 1.13 (0.71-1.79) | X |
|  | Weeks 29-52 | 1.40 (1.11-1.78) | - | - |
|  | Weeks 53-102 | 2.55 (1.85-3.52) | - | - |
| Gallstones | Day 0 | 116 (109-123) | 28.2 (25.4-31.3) | 59.6 (46.4-76.6) |
|  | Weeks 1-4 | 3.18 (2.95-3.42) | 1.49 (1.36-1.64) | 4.10 (3.34-5.05) |
|  | Weeks 5-28 | 1.68 (1.61-1.74) | 1.22 (1.13-1.32) | 2.65 (2.19-3.20) |
|  | Weeks 29-52 | 1.40 (1.34-1.47) | - | - |
|  | Weeks 53-102 | 1.66 (1.54-1.78) | - | - |
| Irritable bowel syndrome | Day 0 | 49.8 (45.9-54.1) | 13.2 (11.4-15.1) | 52.2 (41.9-65.1) |
|  | Weeks 1-4 | 2.48 (2.31-2.68) | 1.29 (1.17-1.42) | 4.76 (4.06-5.57) |
|  | Weeks 5-28 | 1.29 (1.24-1.34) | 1.25 (1.16-1.34) | 2.14 (1.ß2.55) |
|  | Weeks 29-52 | 1.28 (1.23-1.33) | - | - |
|  | Weeks 53-102 | 1.38 (1.29-1.49) | - | - |

## Supplementary Table 5: Adjusted hazard ratios (95% CI) comparing the incidence of gastrointestinal disease events after COVID-19 with the incidence before or without COVID-19, in the pre-vaccination, vaccinated and unvaccinated cohorts, in hospitalised subgroup. Hazard ratios are maximally adjusted.

| Outcome | Time since COVID-19 | Pre-vaccination cohort | Vaccinated cohort | Unvaccinated cohort |
| --- | --- | --- | --- | --- |
| Upper gastrointestinal bleeding | Day 0 | 516 (475-560) | 391 (354-431) | 398 (302-524) |
|  | Weeks 1-4 | 20.1 (18.4-22.1) | 15.2 (13.4-17.3) | 19.7 (15.0-25.9) |
|  | Weeks 5-28 | 2.79 (2.52-3.09) | 3.78 (3.24-4.41) | 2.85 (1.80-4.53) |
|  | Weeks 29-52 | 1.78 (1.57-2.02) | - | - |
|  | Weeks 53-102 | 1.90 (1.63-2.22) | - | - |
| Lower gastrointestinal bleeding | Day 0 | 332 (302-366) | 160 (132-194) | 456 (383-543) |
|  | Weeks 1-4 | 13.7 (12.4-15.2) | 10.4 (8.80-12.2) | 11.4 (8.48-15.4) |
|  | Weeks 5-28 | 2.26 (2.04-2.51) | 3.40 (2.89-4.00) | 3.00 (2.23-4.04) |
|  | Weeks 29-52 | 1.51 (1.32-1.72) | - | - |
|  | Weeks 53-102 | 1.64 (1.39-1.93) | - | - |
| Gastro-oesophageal reflux | Day 0 | 373 (351-395) | 196 (176-218) | 396 (335-468) |
|  | Weeks 1-4 | 23.8 (22.6-25.1) | 15.2 (13.8-16.6) | 25.8 (22.2-29.9) |
|  | Weeks 5-28 | 2.10 (1.95-2.25) | 4.35 (3.88-4.87) | 2.89 (2.18-3.81) |
|  | Weeks 29-52 | 1.65 (1.53-1.78) | - | - |
|  | Weeks 53-102 | 1.65 (1.49-1.83) | - | - |
| Dyspepsia | Day 0 | 128 (108-152) | 73.0 (50.0-106) | 210 (158-280) |
|  | Weeks 1-4 | 7.03 (6.05-8.17) | 5.25 (3.90-7.07) | 9.83 (7.03-13.7) |
|  | Weeks 5-28 | 1.52 (1.33-1.73) | 1.92 (1.34-2.75) | 5.31 (4.21-6.69) |
|  | Weeks 29-52 | 1.56 (1.36-1.79) | - | - |
|  | Weeks 53-102 | 1.42 (1.16-1.73) | - | - |
| Gallstones | Day 0 | 261 (233-292) | 139 (115-169) | 176 (123-253) |
|  | Weeks 1-4 | 12.4 (11.1-13.9) | 9.53 (8.05-11.3) | 25.8 (21.5-31.1) |
|  | Weeks 5-28 | 2.36 (2.13-2.62) | 3.02 (2.53-3.61) | 2.91 (1.97-4.30) |
|  | Weeks 29-52 | 1.53 (1.34-1.74) | - | - |
|  | Weeks 53-102 | 1.83 (1.57-2.13) | - | - |
| Irritable bowel syndrome | Day 0 | 369 (327-417) | 232 (189-284) | 378 (290-493) |
|  | Weeks 1-4 | 23.0 (20.8-25.5) | 16.7 (13.8-20.3) | 27.3 (22.1-33.9) |
|  | Weeks 5-28 | 1.58 (1.36-1.84) | 3.52 (2.67-4.64) | 3.30 (2.23-4.88) |
|  | Weeks 29-52 | 1.42 (1.21-1.66) | - | - |
|  | Weeks 53-102 | 1.49 (1.21-1.84) | - | - |

X insufficient events

## Supplementary Table 6: Adjusted hazard ratios (95% CI) comparing the incidence of gastrointestinal disease events after COVID-19 with the incidence before or without COVID-19, in the pre-vaccination, vaccinated and unvaccinated cohorts, in non-hospitalised subgroup. Hazard ratios are maximally adjusted.

| Outcome | Time since COVID-19 | Pre-vaccination cohort | Vaccinated cohort | Unvaccinated cohort |
| --- | --- | --- | --- | --- |
| Upper gastrointestinal bleeding | Day 0 | 131 (123-139) | 41.1 (37.1-45.6) | 61.7 (47.7-79.7) |
|  | Weeks 1-4 | 1.92 (1.74-2.11) | 1.20 (1.06-1.36) | 2.14 (1.61-2.85) |
|  | Weeks 5-28 | 1.46 (1.40-1.53) | 1.14 (1.04-1.25) | 1.15 (0.88-1.51) |
|  | Weeks 29-52 | 1.23 (1.18-1.29) | - | - |
|  | Weeks 53-102 | 1.34 (1.23-1.45) | - | - |
| Lower gastrointestinal bleeding | Day 0 | 65.7 (61.2-70.5) | 23.0 (20.5-25.8) | 31.2 (23.0-42.4) |
|  | Weeks 1-4 | 1.44 (1.32-1.59) | 1.02 (0.91-1.14) | 1.82 (1.39-2.38) |
|  | Weeks 5-28 | 1.39 (1.34-1.44) | 1.15 (1.07-1.24) | 1.28 (1.02-1.60) |
|  | Weeks 29-52 | 1.22 (1.17-1.27) | - | - |
|  | Weeks 53-102 | 1.30 (1.21-1.40) | - | - |
| Gastro-oesophageal reflux | Day 0 | 37.4 (35.2-39.9) | 11.8 (10.8-13.0) | 16.1 (12.3-21.2) |
|  | Weeks 1-4 | 1.46 (1.37-1.55) | 0.91 (0.84-0.97) | 1.40 (1.15-1.70) |
|  | Weeks 5-28 | 1.28 (1.25-1.32) | 1.14 (1.09-1.20) | 1.48 (1.29-1.70) |
|  | Weeks 29-52 | 1.17 (1.13-1.20) | - | - |
|  | Weeks 53-102 | 1.28 (1.22-1.34) | - | - |
| Dyspepsia | Day 0 | 20.3 (18.0-22.9) | 5.37 (4.23-6.81) | 20.2 (14.4-28.4) |
|  | Weeks 1-4 | 1.25 (1.14-1.38) | 0.92 (0.81-1.04) | 1.52 (1.17-1.98) |
|  | Weeks 5-28 | 1.23 (1.19-1.28) | 1.10 (1.02-1.20) | 1.40 (1.15-1.72) |
|  | Weeks 29-52 | 1.20 (1.15-1.25) | - | - |
|  | Weeks 53-102 | 1.22 (1.13-1.32) | - | - |
| Gallstones | Day 0 | 78.0 (72.4-84.1) | 20.5 (18.1-23.1) | 27.2 (19.5-37.9) |
|  | Weeks 1-4 | 1.67 (1.51-1.85) | 0.97 (0.86-1.09) | 1.48 (1.09-2.02) |
|  | Weeks 5-28 | 1.36 (1.30-1.42) | 1.06 (0.98-1.14) | 1.69 (1.37-2.09) |
|  | Weeks 29-52 | 1.17 (1.12-1.23) | - | - |
|  | Weeks 53-102 | 1.25 (1.16-1.36) | - | - |
| Irritable bowel syndrome | Day 0 | 22.3 (19.7-25.1) | 7.47 (6.24-8.94) | 10.8 (7.13-16.2) |
|  | Weeks 1-4 | 1.08 (0.97-1.21) | 0.78 (0.69-0.87) | 1.45 (1.14-1.85) |
|  | Weeks 5-28 | 1.12 (1.07-1.16) | 1.05 (0.98-1.13) | 1.18 (0.97-1.43) |
|  | Weeks 29-52 | 1.10 (1.06-1.15) | - | - |
|  | Weeks 53-102 | 1.11 (1.03-1.20) | - | - |

X insufficient events

## Supplementary Table 7: Adjusted hazard ratios (95% CI) comparing the incidence of gastrointestinal disease events after COVID-19 with the incidence before or without COVID-19, in the pre-vaccination, vaccinated and unvaccinated cohorts, in 18–39 age group. Hazard ratios are maximally adjusted.

| Outcome | Time since COVID-19 | Pre-vaccination cohort | Vaccinated cohort | Unvaccinated cohort |
| --- | --- | --- | --- | --- |
| Upper gastrointestinal bleeding | Day 0 | 20.3 (15.3-26.9) | 7.61 (4.50-12.9) | 37.9 (24.9-57.9) |
|  | Weeks 1-4 | 1.69 (1.40-2.04) | 1.26 (0.95-1.67) | 2.81 (2.01-3.92) |
|  | Weeks 5-28 | 1.25 (1.15-1.36) | 1.19 (0.97-1.47) | 1.51 (1.07-2.15) |
|  | Weeks 29-52 | 1.19 (1.08-1.30) | - | - |
|  | Weeks 53-102 | 1.35 (1.14-1.61) | - | - |
| Lower gastrointestinal bleeding | Day 0 | 8.91 (6.56-12.1) | 2.78 (1.45-5.36) | 11.4 (6.05-21.6) |
|  | Weeks 1-4 | 1.16 (0.99-1.37) | 0.88 (0.68-1.14) | 1.88 (1.34-2.65) |
|  | Weeks 5-28 | 1.27 (1.19-1.35) | 1.30 (1.12-1.51) | 1.49 (1.10-2.00) |
|  | Weeks 29-52 | 1.21 (1.13-1.30) | - | - |
|  | Weeks 53-102 | 1.40 (1.22-1.60) | - | - |
| Gastro-oesophageal reflux | Day 0 | 1.43 (1.28-1.60) | 0.85 (0.71-1.02) | 2.23 (1.81-2.76) |
|  | Weeks 1-4 | 1.19 (1.13-1.25) | 1.07 (0.95-1.20) | 1.64 (1.36-1.98) |
|  | Weeks 5-28 | 1.22 (1.16-1.28) | - | - |
|  | Weeks 29-52 | 1.38 (1.25-1.52) | - | - |
|  | Weeks 53-102 | 1.43 (1.28-1.60) | 0.85 (0.71-1.02) | 2.23 (1.81-2.76) |
| Dyspepsia | Day 0 | 12.6 (9.90-16.1) | 3.05 (1.64-5.67) | 20.9 (14.0-31.4) |
|  | Weeks 1-4 | 1.36 (1.18-1.58) | 0.87 (0.67-1.12) | 1.58 (1.15-2.17) |
|  | Weeks 5-28 | 1.20 (1.13-1.28) | 1.18 (1.01-1.38) | 1.51 (1.17-1.95) |
|  | Weeks 29-52 | 1.22 (1.14-1.30) | - | - |
|  | Weeks 53-102 | 1.24 (1.09-1.42) | - | - |
| Gallstones | Day 0 | 14.3 (10.4-19.6) | 3.51 (1.89-6.53) | 8.94 (4.43-18.0) |
|  | Weeks 1-4 | 1.33 (1.08-1.63) | 0.70 (0.52-0.95) | 1.33 (0.89-1.99) |
|  | Weeks 5-28 | 1.13 (1.04-1.23) | 0.96 (0.79-1.16) | 1.49 (1.11-2.02) |
|  | Weeks 29-52 | 1.12 (1.03-1.22) | - | - |
|  | Weeks 53-102 | 1.27 (1.08-1.51) | - | - |
| Irritable bowel syndrome | Day 0 | 10.7 (8.39-13.6) | 2.50 (1.48-4.23) | 10.2 (6.20-16.7) |
|  | Weeks 1-4 | 1.16 (1.01-1.34) | 0.89 (0.73-1.08) | 1.96 (1.54-2.50) |
|  | Weeks 5-28 | 1.10 (1.04-1.17) | 1.13 (1.00-1.28) | 1.11 (0.86-1.43) |
|  | Weeks 29-52 | 1.16 (1.09-1.23) | - | - |
|  | Weeks 53-102 | 1.20 (1.07-1.35) | - | - |

X insufficient events

## Supplementary Table 8: Adjusted hazard ratios (95% CI) comparing the incidence of gastrointestinal disease events after COVID-19 with the incidence before or without COVID-19, in the pre-vaccination, vaccinated and unvaccinated cohorts, in 40–59 age group. Hazard ratios are maximally adjusted.

| Outcome | Time since COVID-19 | Pre-vaccination cohort | Vaccinated cohort | Unvaccinated cohort |
| --- | --- | --- | --- | --- |
| Upper gastrointestinal bleeding | Day 0 | 20.3 (15.3-26.9) | 7.61 (4.50-12.9) | 37.9 (24.9-57.9) |
|  | Weeks 1-4 | 1.69 (1.40-2.04) | 1.26 (0.95-1.67) | 2.81 (2.01-3.92) |
|  | Weeks 5-28 | 1.25 (1.15-1.36) | 1.19 (0.97-1.47) | 1.51 (1.07-2.15) |
|  | Weeks 29-52 | 1.19 (1.08-1.30) | - | - |
|  | Weeks 53-102 | 1.35 (1.14-1.61) | - | - |
| Lower gastrointestinal bleeding | Day 0 | 8.91 (6.56-12.1) | 2.78 (1.45-5.36) | 11.4 (6.05-21.6) |
|  | Weeks 1-4 | 1.16 (0.99-1.37) | 0.88 (0.68-1.14) | 1.88 (1.34-2.65) |
|  | Weeks 5-28 | 1.27 (1.19-1.35) | 1.30 (1.12-1.51) | 1.49 (1.10-2.00) |
|  | Weeks 29-52 | 1.21 (1.13-1.30) | - | - |
|  | Weeks 53-102 | 1.40 (1.22-1.60) | - | - |
| Gastro-oesophageal reflux | Day 0 | 1.43 (1.28-1.60) | 0.85 (0.71-1.02) | 2.23 (1.81-2.76) |
|  | Weeks 1-4 | 1.19 (1.13-1.25) | 1.07 (0.95-1.20) | 1.64 (1.36-1.98) |
|  | Weeks 5-28 | 1.22 (1.16-1.28) | - | - |
|  | Weeks 29-52 | 1.38 (1.25-1.52) | - | - |
|  | Weeks 53-102 | 1.43 (1.28-1.60) | 0.85 (0.71-1.02) | 2.23 (1.81-2.76) |
| Dyspepsia | Day 0 | 12.6 (9.90-16.1) | 3.05 (1.64-5.67) | 20.9 (14.0-31.4) |
|  | Weeks 1-4 | 1.36 (1.18-1.58) | 0.87 (0.67-1.12) | 1.58 (1.15-2.17) |
|  | Weeks 5-28 | 1.20 (1.13-1.28) | 1.18 (1.01-1.38) | 1.51 (1.17-1.95) |
|  | Weeks 29-52 | 1.22 (1.14-1.30) | - | - |
|  | Weeks 53-102 | 1.24 (1.09-1.42) | - | - |
| Gallstones | Day 0 | 14.3 (10.4-19.6) | 3.51 (1.89-6.53) | 8.94 (4.43-18.0) |
|  | Weeks 1-4 | 1.33 (1.08-1.63) | 0.70 (0.52-0.95) | 1.33 (0.89-1.99) |
|  | Weeks 5-28 | 1.13 (1.04-1.23) | 0.96 (0.79-1.16) | 1.49 (1.11-2.02) |
|  | Weeks 29-52 | 1.12 (1.03-1.22) | - | - |
|  | Weeks 53-102 | 1.27 (1.08-1.51) | - | - |
| Irritable bowel syndrome | Day 0 | 10.7 (8.39-13.6) | 2.50 (1.48-4.23) | 10.2 (6.20-16.7) |
|  | Weeks 1-4 | 1.16 (1.01-1.34) | 0.89 (0.73-1.08) | 1.96 (1.54-2.50) |
|  | Weeks 5-28 | 1.10 (1.04-1.17) | 1.13 (1.00-1.28) | 1.11 (0.86-1.43) |
|  | Weeks 29-52 | 1.16 (1.09-1.23) | - | - |
|  | Weeks 53-102 | 1.20 (1.07-1.35) | - | - |

X insufficient events

## Supplementary Table 9: Adjusted hazard ratios (95% CI) comparing the incidence of gastrointestinal disease events after COVID-19 with the incidence before or without COVID-19, in the pre-vaccination, vaccinated and unvaccinated cohorts, in 60–79 age group. Hazard ratios are maximally adjusted.

| Outcome | Time since COVID-19 | Pre-vaccination cohort | Vaccinated cohort | Unvaccinated cohort |
| --- | --- | --- | --- | --- |
| Upper gastrointestinal bleeding | Day 0 | 322 (299-347) | 70.2 (60.9-81.0) | 382 (278-524) |
|  | Weeks 1-4 | 13.7 (12.9-14.5) | 2.23 (1.90-2.62) | 8.78 (5.71-13.5) |
|  | Weeks 5-28 | 2.40 (2.25-2.56) | 1.19 (1.02-1.39) | 1.17 (0.55-2.48) |
|  | Weeks 29-52 | 1.41 (1.29-1.53) | - | - |
|  | Weeks 53-102 | 2.00 (1.81-2.22) | - | - |
| Lower gastrointestinal bleeding | Day 0 | 192 (176-210) | 48.5 (41.6-56.4) | 260 (181-373) |
|  | Weeks 1-4 | 4.75 (4.24-5.31) | 1.92 (1.63-2.25) | 13.0 (9.86-17.1) |
|  | Weeks 5-28 | 1.70 (1.58-1.83) | 1.14 (0.98-1.32) | 1.39 (0.73-2.64) |
|  | Weeks 29-52 | 1.26 (1.16-1.38) | - | - |
|  | Weeks 53-102 | 1.54 (1.35-1.75) | - | - |
| Gastro-oesophageal reflux | Day 0 | 131 (123-140) | 25.7 (22.9-28.9) | 243 (194-305) |
|  | Weeks 1-4 | 5.68 (5.33-6.05) | 1.83 (1.67-2.00) | 13.3 (10.8-16.5) |
|  | Weeks 5-28 | 1.52 (1.45-1.59) | 1.28 (1.19-1.38) | 2.65 (2.02-3.48) |
|  | Weeks 29-52 | 1.20 (1.15-1.27) | - | - |
|  | Weeks 53-102 | 1.37 (1.27-1.48) | - | - |
| Dyspepsia | Day 0 | 51.4 (43.4-60.7) | 12.6 (9.32-17.1) | X |
|  | Weeks 1-4 | 2.35 (2.01-2.76) | 1.20 (0.98-1.49) | X |
|  | Weeks 5-28 | 1.30 (1.20-1.42) | 1.13 (0.97-1.32) | X |
|  | Weeks 29-52 | 1.21 (1.10-1.32) | - | - |
|  | Weeks 53-102 | 1.26 (1.08-1.46) | - | - |
| Gallstones | Day 0 | 182 (166-200) | 40.7 (34.7-47.7) | 285 (209-388) |
|  | Weeks 1-4 | 4.29 (3.79-4.85) | 1.91 (1.65-2.23) | 13.2 (9.93-17.7) |
|  | Weeks 5-28 | 1.73 (1.61-1.87) | 1.16 (1.01-1.33) | 3.08 (2.01-4.70) |
|  | Weeks 29-52 | 1.31 (1.21-1.42) | - | - |
|  | Weeks 53-102 | 1.35 (1.18-1.54) | - | - |
| Irritable bowel syndrome | Day 0 | 122 (106-140) | 28.4 (22.8-35.3) | 342 (248-472) |
|  | Weeks 1-4 | 5.13 (4.47-5.89) | 1.57 (1.30-1.90) | 15.1 (10.5-21.9) |
|  | Weeks 5-28 | 1.24 (1.12-1.38) | 1.12 (0.96-1.31) | 7.68 (5.65-10.5) |
|  | Weeks 29-52 | 1.13 (1.02-1.25) | - | - |
|  | Weeks 53-102 | 1.00 (0.84-1.19) | - | - |

X insufficient events

## Supplementary Table 10: Adjusted hazard ratios (95% CI) comparing the incidence of gastrointestinal disease events after COVID-19 with the incidence before or without COVID-19, in the pre-vaccination, vaccinated and unvaccinated cohorts, in 80–110 age group. Hazard ratios are maximally adjusted.

| Outcome | Time since COVID-19 | Pre-vaccination cohort | Vaccinated cohort | Unvaccinated cohort |
| --- | --- | --- | --- | --- |
| Upper gastrointestinal bleeding | Day 0 | 398 (366-432) | 213 (187-242) | - |
|  | Weeks 1-4 | 4.86 (4.18-5.65) | 4.39 (3.64-5.30) | - |
|  | Weeks 5-28 | 2.02 (1.83-2.24) | 1.37 (1.08-1.73) | - |
|  | Weeks 29-52 | 1.41 (1.24-1.59) | - | - |
|  | Weeks 53-102 | 1.31 (1.12-1.52) | - | - |
| Lower gastrointestinal bleeding | Day 0 | 316 (287-348) | 196 (170-226) | - |
|  | Weeks 1-4 | 4.82 (4.11-5.66) | 3.71 (3.00-4.59) | - |
|  | Weeks 5-28 | 1.85 (1.65-2.07) | 1.21 (0.93-1.57) | - |
|  | Weeks 29-52 | 1.31 (1.14-1.50) | - | - |
|  | Weeks 53-102 | 1.23 (1.04-1.46) | - | - |
| Gastro-oesophageal reflux | Day 0 | 399 (376-424) | 123 (109-138) | - |
|  | Weeks 1-4 | 12.2 (11.5-13.0) | 3.72 (3.23-4.29) | - |
|  | Weeks 5-28 | 1.94 (1.79-2.10) | 1.55 (1.33-1.80) | - |
|  | Weeks 29-52 | 1.47 (1.34-1.62) | - | - |
|  | Weeks 53-102 | 1.28 (1.14-1.44) | - | - |
| Dyspepsia | Day 0 | 98.1 (77.5-124) | 65.3 (46.6-91.5) | - |
|  | Weeks 1-4 | 2.39 (1.73-3.29) | 2.84 (2.01-4.03) | - |
|  | Weeks 5-28 | 1.45 (1.21-1.74) | 1.65 (1.19-2.29) | - |
|  | Weeks 29-52 | 1.16 (0.93-1.44) | - | - |
|  | Weeks 53-102 | 1.14 (0.87-1.48) | - | - |
| Gallstones | Day 0 | 252 (226-281) | 135 (115-159) | - |
|  | Weeks 1-4 | 4.29 (3.62-5.09) | 2.68 (2.11-3.40) | - |
|  | Weeks 5-28 | 1.81 (1.62-2.03) | 1.57 (1.26-1.97) | - |
|  | Weeks 29-52 | 1.16 (1.00-1.34) | - | - |
|  | Weeks 53-102 | 1.20 (1.02-1.42) | - | - |
| Irritable bowel syndrome | Day 0 | 284 (235-342) | 109 (82.6-145) | - |
|  | Weeks 1-4 | 6.22 (4.80-8.06) | 3.73 (2.72-5.10) | - |
|  | Weeks 5-28 | 1.56 (1.27-1.91) | 1.45 (1.02-2.06) | - |
|  | Weeks 29-52 | 1.31 (1.06-1.62) | - | - |
|  | Weeks 53-102 | 1.38 (1.08-1.75) | - | - |

X insufficient events

## Supplementary Table 11: Adjusted hazard ratios (95% CI) comparing the incidence of gastrointestinal disease events after COVID-19 with the incidence before or without COVID-19, in the pre-vaccination, vaccinated and unvaccinated cohorts, in White ethnicity. Hazard ratios are maximally adjusted.

| Subgroup and outcome | Time since COVID-19 | Pre-vaccination cohort | Vaccinated cohort | Unvaccinated cohort |
| --- | --- | --- | --- | --- |
| Upper gastrointestinal bleeding | Day 0 | 187 (177-197) | 50.9 (46.3-55.9) | 94.9 (76.9-117) |
|  | Weeks 1-4 | 3.53 (3.28-3.80) | 1.83 (1.65-2.02) | 3.46 (2.75-4.35) |
|  | Weeks 5-28 | 1.57 (1.50-1.64) | 1.18 (1.08-1.29) | 1.26 (0.97-1.65) |
|  | Weeks 29-52 | 1.28 (1.22-1.34) | - | - |
|  | Weeks 53-102 | 1.42 (1.31-1.53) | - | - |
| Lower gastrointestinal bleeding | Day 0 | 96.3 (90.6-102) | 29.2 (26.3-32.4) | 52.9 (41.6-67.4) |
|  | Weeks 1-4 | 2.45 (2.27-2.64) | 1.42 (1.29-1.57) | 2.45 (1.93-3.10) |
|  | Weeks 5-28 | 1.43 (1.38-1.49) | 1.18 (1.09-1.27) | 1.17 (0.92-1.49) |
|  | Weeks 29-52 | 1.25 (1.20-1.31) | - | - |
|  | Weeks 53-102 | 1.31 (1.22-1.42) | - | - |
|  | Day 0 | 75.0 (71.5-78.6) | 18.4 (17.0-20.0) | 47.5 (40.0-56.3) |
| Gastro-oesophageal reflux | Weeks 1-4 | 3.26 (3.12-3.41) | 1.43 (1.35-1.52) | 3.55 (3.10-4.06) |
|  | Weeks 5-28 | 1.35 (1.32-1.39) | 1.19 (1.14-1.25) | 1.59 (1.38-1.84) |
|  | Weeks 29-52 | 1.18 (1.15-1.22) | - | - |
|  | Weeks 53-102 | 1.34 (1.28-1.41) | - | - |
| Dyspepsia | Day 0 | 30.0 (26.9-33.5) | 8.42 (6.96-10.2) | 21.3 (14.9-30.4) |
|  | Weeks 1-4 | 1.50 (1.36-1.65) | 1.05 (0.93-1.18) | 1.95 (1.52-2.51) |
|  | Weeks 5-28 | 1.24 (1.19-1.30) | 1.13 (1.04-1.23) | 1.41 (1.13-1.75) |
|  | Weeks 29-52 | 1.20 (1.15-1.26) | - | - |
|  | Weeks 53-102 | 1.23 (1.13-1.33) | - | - |
| Gallstones | Day 0 | 104 (97.7-111) | 26.6 (23.9-29.7) | 37.2 (27.9-49.7) |
|  | Weeks 1-4 | 2.73 (2.52-2.96) | 1.39 (1.26-1.54) | 2.77 (2.20-3.50) |
|  | Weeks 5-28 | 1.49 (1.42-1.55) | 1.10 (1.01-1.19) | 1.73 (1.40-2.14) |
|  | Weeks 29-52 | 1.20 (1.15-1.26) | - | - |
|  | Weeks 53-102 | 1.34 (1.24-1.45) | - | - |
| Irritable bowel syndrome | Day 0 | 44.3 (40.5-48.5) | 13.8 (12.1-15.7) | 32.6 (25.6-41.5) |
|  | Weeks 1-4 | 2.18 (2.01-2.36) | 1.16 (1.05-1.28) | 2.92 (2.45-3.48) |
|  | Weeks 5-28 | 1.15 (1.10-1.20) | 1.09 (1.01-1.17) | 1.35 (1.12-1.63) |
|  | Weeks 29-52 | 1.11 (1.06-1.16) | - | - |
|  | Weeks 53-102 | 1.14 (1.05-1.23) | - | - |

X insufficient events

## Supplementary Table 12: Adjusted hazard ratios (95% CI) comparing the incidence of gastrointestinal disease events after COVID-19 with the incidence before or without COVID-19, in the pre-vaccination, vaccinated and unvaccinated cohorts, in South Asian ethnicity. Hazard ratios are maximally adjusted.

| Subgroup and outcome | Time since COVID-19 | Pre-vaccination cohort | Vaccinated cohort | Unvaccinated cohort |
| --- | --- | --- | --- | --- |
| Upper gastrointestinal bleeding | Day 0 | 120 (96.3-149) | 72.7 (49.0-108) | X |
|  | Weeks 1-4 | 5.87 (4.83-7.14) | 2.01 (1.24-3.25) | X |
|  | Weeks 5-28 | 1.43 (1.23-1.67) | 1.36 (0.91-2.02) | X |
|  | Weeks 29-52 | 1.32 (1.13-1.54) | - | - |
|  | Weeks 53-102 | 1.36 (1.05-1.76) | - | - |
| Lower gastrointestinal bleeding | Day 0 | 57.4 (45.2-72.9) | 37.1 (24.2-57.0) | X |
|  | Weeks 1-4 | 2.83 (2.29-3.51) | 1.02 (0.59-1.77) | X |
|  | Weeks 5-28 | 1.45 (1.29-1.63) | 1.16 (0.81-1.65) | X |
|  | Weeks 29-52 | 1.21 (1.06-1.38) | - | - |
|  | Weeks 53-102 | 1.53 (1.25-1.88) | - | - |
|  | Day 0 | 54.1 (47.0-62.3) | 20.7 (15.3-28.2) | 127 (90.4-177) |
| Gastro-oesophageal reflux | Weeks 1-4 | 3.81 (3.42-4.23) | 1.33 (1.04-1.72) | 6.38 (4.64-8.77) |
|  | Weeks 5-28 | 1.26 (1.18-1.36) | 1.21 (1.01-1.45) | 2.18 (1.53-3.10) |
|  | Weeks 29-52 | 1.22 (1.14-1.31) | - | - |
|  | Weeks 53-102 | 1.25 (1.11-1.41) | - | - |
| Dyspepsia | Day 0 | 21.4 (16.0-28.5) | 7.05 (3.17-15.7) | X |
|  | Weeks 1-4 | 2.12 (1.77-2.54) | 0.96 (0.61-1.51) | X |
|  | Weeks 5-28 | 1.31 (1.19-1.43) | 1.00 (0.74-1.35) | X |
|  | Weeks 29-52 | 1.25 (1.13-1.38) | - | - |
|  | Weeks 53-102 | 1.31 (1.10-1.56) | - | - |
| Gallstones | Day 0 | 56.3 (42.9-73.9) | 16.8 (8.69-32.3) | X |
|  | Weeks 1-4 | 2.59 (2.02-3.33) | 1.53 (0.97-2.40) | X |
|  | Weeks 5-28 | 1.27 (1.10-1.45) | 0.91 (0.61-1.35) | X |
|  | Weeks 29-52 | 1.17 (1.01-1.35) | - | - |
|  | Weeks 53-102 | 1.36 (1.08-1.70) | - | - |
| Irritable bowel syndrome | Day 0 | 30.1 (20.9-43.3) | X | X |
|  | Weeks 1-4 | 2.32 (1.79-3.01) | X | X |
|  | Weeks 5-28 | 1.08 (0.93-1.25) | X | X |
|  | Weeks 29-52 | 1.13 (0.98-1.31) | - | - |
|  | Weeks 53-102 | 1.10 (0.85-1.42) | - | - |

X insufficient events

## Supplementary Table 13: Adjusted hazard ratios (95% CI) comparing the incidence of gastrointestinal disease events after COVID-19 with the incidence before or without COVID-19, in the pre-vaccination, vaccinated and unvaccinated cohorts, in Female sex. Hazard ratios are maximally adjusted.

| Subgroup and outcome | Time since COVID-19 | Pre-vaccination cohort | Vaccinated cohort | Unvaccinated cohort |
| --- | --- | --- | --- | --- |
| Upper gastrointestinal bleeding | Day 0 | 148 (137-160) | 44.0 (38.4-50.4) | 98.4 (75.3-129) |
|  | Weeks 1-4 | 2.81 (2.53-3.13) | 1.62 (1.40-1.87) | 4.27 (3.26-5.58) |
|  | Weeks 5-28 | 1.48 (1.39-1.56) | 1.23 (1.10-1.39) | 1.22 (0.86-1.72) |
|  | Weeks 29-52 | 1.24 (1.17-1.33) | - | - |
|  | Weeks 53-102 | 1.36 (1.23-1.50) | - | - |
| Lower gastrointestinal bleeding | Day 0 | 76.1 (69.7-83.0) | 23.5 (20.2-27.4) | 50.4 (36.9-68.8) |
|  | Weeks 1-4 | 1.89 (1.70-2.11) | 1.20 (1.04-1.38) | 2.62 (1.97-3.50) |
|  | Weeks 5-28 | 1.35 (1.28-1.42) | 1.22 (1.11-1.35) | 1.18 (0.88-1.60) |
|  | Weeks 29-52 | 1.20 (1.13-1.27) | - | - |
|  | Weeks 53-102 | 1.25 (1.14-1.38) | - | - |
| Gastro-oesophageal reflux | Day 0 | 58.5 (54.9-62.2) | 18.7 (17.1-20.5) | 46.8 (38.5-56.8) |
|  | Weeks 1-4 | 2.90 (2.75-3.07) | 1.23 (1.13-1.33) | 3.12 (2.65-3.67) |
|  | Weeks 5-28 | 1.29 (1.25-1.33) | 1.16 (1.10-1.23) | 1.51 (1.28-1.77) |
|  | Weeks 29-52 | 1.17 (1.14-1.21) | - | - |
|  | Weeks 53-102 | 1.29 (1.22-1.36) | - | - |
| Dyspepsia | Day 0 | 24.6 (21.5-28.0) | 7.51 (5.92-9.52) | 30.7 (22.4-42.2) |
|  | Weeks 1-4 | 1.60 (1.45-1.78) | 0.94 (0.81-1.08) | 2.03 (1.56-2.64) |
|  | Weeks 5-28 | 1.24 (1.18-1.29) | 1.11 (1.01-1.23) | 1.58 (1.27-1.97) |
|  | Weeks 29-52 | 1.20 (1.14-1.26) | - | - |
|  | Weeks 53-102 | 1.23 (1.13-1.35) | - | - |
| Gallstones | Day 0 | 75.1 (68.9-81.8) | 20.2 (17.5-23.4) | 35.1 (26.0-47.4) |
|  | Weeks 1-4 | 2.19 (1.98-2.42) | 1.20 (1.06-1.36) | 2.06 (1.57-2.69) |
|  | Weeks 5-28 | 1.35 (1.29-1.42) | 1.07 (0.97-1.17) | 1.69 (1.36-2.10) |
|  | Weeks 29-52 | 1.17 (1.11-1.24) | - | - |
|  | Weeks 53-102 | 1.31 (1.21-1.43) | - | - |
| Irritable bowel syndrome | Day 0 | 38.3 (34.5-42.6) | 11.8 (10.0-13.8) | 32.2 (24.8-41.7) |
|  | Weeks 1-4 | 2.04 (1.86-2.23) | 1.10 (0.98-1.23) | 3.12 (2.61-3.74) |
|  | Weeks 5-28 | 1.11 (1.06-1.16) | 1.12 (1.03-1.21) | 1.38 (1.14-1.68) |
|  | Weeks 29-52 | 1.10 (1.05-1.15) | - | - |
|  | Weeks 53-102 | 1.12 (1.03-1.22) | - | - |

X insufficient events

## Supplementary Table 14: Adjusted hazard ratios (95% CI) comparing the incidence of gastrointestinal disease events after COVID-19 with the incidence before or without COVID-19, in the pre-vaccination, vaccinated and unvaccinated cohorts, in Male sex. Hazard ratios are maximally adjusted.

| Subgroup and outcome | Time since COVID-19 | Pre-vaccination cohort | Vaccinated cohort | Unvaccinated cohort |
| --- | --- | --- | --- | --- |
| Upper gastrointestinal bleeding | Day 0 | 216 (202-231) | 64.4 (57.5-72.1) | 114 (87.3-148) |
|  | Weeks 1-4 | 5.04 (4.63-5.49) | 2.10 (1.83-2.39) | 4.22 (3.17-5.61) |
|  | Weeks 5-28 | 1.72 (1.62-1.82) | 1.17 (1.03-1.32) | 1.60 (1.16-2.21) |
|  | Weeks 29-52 | 1.34 (1.26-1.43) | - | - |
|  | Weeks 53-102 | 1.53 (1.38-1.70) | - | - |
| Lower gastrointestinal bleeding | Day 0 | 110 (102-119) | 36.6 (32.1-41.7) | 64.2 (47.0-87.6) |
|  | Weeks 1-4 | 3.17 (2.90-3.47) | 1.66 (1.46-1.89) | 3.23 (2.41-4.34) |
|  | Weeks 5-28 | 1.58 (1.50-1.67) | 1.16 (1.04-1.29) | 1.61 (1.20-2.15) |
|  | Weeks 29-52 | 1.30 (1.22-1.37) | - | - |
|  | Weeks 53-102 | 1.46 (1.32-1.61) | - | - |
| Gastro-oesophageal reflux | Day 0 | 88.9 (83.6-94.5) | 22.5 (20.2-25.0) | 71.8 (58.4-88.3) |
|  | Weeks 1-4 | 4.05 (3.83-4.29) | 1.71 (1.58-1.85) | 5.06 (4.27-6.00) |
|  | Weeks 5-28 | 1.42 (1.37-1.48) | 1.26 (1.18-1.35) | 1.94 (1.60-2.34) |
|  | Weeks 29-52 | 1.23 (1.18-1.28) | - | - |
|  | Weeks 53-102 | 1.36 (1.27-1.46) | - | - |
| Dyspepsia | Day 0 | 35.8 (30.7-41.6) | 8.22 (6.02-11.2) | 32.3 (20.2-51.6) |
|  | Weeks 1-4 | 1.73 (1.51-1.98) | 1.21 (1.01-1.45) | 2.75 (1.94-3.90) |
|  | Weeks 5-28 | 1.28 (1.20-1.36) | 1.14 (1.00-1.30) | 1.53 (1.09-2.14) |
|  | Weeks 29-52 | 1.26 (1.17-1.34) | - | - |
|  | Weeks 53-102 | 1.27 (1.12-1.44) | - | - |
| Gallstones | Day 0 | 149 (136-164) | 39.8 (34.1-46.5) | 80.0 (50.4-127) |
|  | Weeks 1-4 | 3.97 (3.54-4.44) | 1.75 (1.50-2.04) | 7.00 (4.93-9.93) |
|  | Weeks 5-28 | 1.64 (1.53-1.76) | 1.14 (0.99-1.30) | 1.84 (1.17-2.90) |
|  | Weeks 29-52 | 1.26 (1.17-1.37) | - | - |
|  | Weeks 53-102 | 1.40 (1.23-1.58) | - | - |
| Irritable bowel syndrome | Day 0 | 53.7 (46.0-62.6) | 16.2 (12.7-20.8) | 54.9 (36.4-83.0) |
|  | Weeks 1-4 | 2.75 (2.41-3.15) | 1.48 (1.24-1.77) | 3.98 (2.87-5.51) |
|  | Weeks 5-28 | 1.24 (1.15-1.34) | 1.05 (0.90-1.22) | 1.24 (0.82-1.88) |
|  | Weeks 29-52 | 1.15 (1.06-1.25) | - | - |
|  | Weeks 53-102 | 1.13 (0.97-1.31) | - | - |

X insufficient events

## Supplementary Table 15: Adjusted hazard ratios (95% CI) comparing the incidence of gastrointestinal disease events after COVID-19 with the incidence before or without COVID-19, in the pre-vaccination, vaccinated and unvaccinated cohorts, in individuals with prior history of gastrointestinal diseases. Hazard ratios are maximally adjusted.

| Outcome | Time since COVID-19 | Pre-vaccination cohort | Vaccinated cohort | Unvaccinated cohort |
| --- | --- | --- | --- | --- |
| Upper gastrointestinal bleeding | Day 0 | 160 (151-170) | 47.4 (42.6-52.7) | 84.1 (66.8-106) |
|  | Weeks 1-4 | 3.36 (3.09-3.64) | 1.84 (1.64-2.05) | 3.00 (2.33-3.88) |
|  | Weeks 5-28 | 1.53 (1.46-1.61) | 1.17 (1.06-1.29) | 1.25 (0.95-1.64) |
|  | Weeks 29-52 | 1.25 (1.18-1.32) | - | - |
|  | Weeks 53-102 | 1.47 (1.35-1.60) | - | - |
| Lower gastrointestinal bleeding | Day 0 | 83.7 (78.0-89.8) | 27.6 (24.5-31.1) | 44.3 (33.7-58.2) |
|  | Weeks 1-4 | 2.31 (2.13-2.51) | 1.42 (1.27-1.58) | 2.20 (1.69-2.85) |
|  | Weeks 5-28 | 1.41 (1.35-1.47) | 1.14 (1.05-1.24) | 1.22 (0.95-1.56) |
|  | Weeks 29-52 | 1.19 (1.14-1.25) | - | - |
|  | Weeks 53-102 | 1.34 (1.24-1.45) | - | - |
| Gastro-oesophageal reflux | Weeks 1-4 | 76.3 (72.8-79.9) | 19.8 (18.3-21.4) | 56.8 (48.8-66.2) |
|  | Weeks 5-28 | 3.49 (3.34-3.64) | 1.46 (1.37-1.55) | 3.78 (3.33-4.29) |
|  | Weeks 29-52 | 1.34 (1.30-1.37) | 1.20 (1.15-1.26) | 1.56 (1.36-1.79) |
|  | Weeks 53-102 | 1.19 (1.15-1.22) | - | - |
|  | Weeks 1-4 | 1.31 (1.25-1.37) | - | - |
| Dyspepsia | Day 0 | 26.7 (23.8-29.9) | 9.39 (7.80-11.3) | 26.0 (18.9-35.7) |
|  | Weeks 1-4 | 1.56 (1.42-1.72) | 1.02 (0.90-1.16) | 1.91 (1.49-2.46) |
|  | Weeks 5-28 | 1.22 (1.17-1.27) | 1.11 (1.02-1.21) | 1.44 (1.17-1.78) |
|  | Weeks 29-52 | 1.19 (1.14-1.25) | - | - |
|  | Weeks 53-102 | 1.25 (1.15-1.36) | - | - |
| Gallstones | Day 0 | 94.6 (87.9-102) | 24.4 (21.6-27.5) | 33.2 (24.6-44.9) |
|  | Weeks 1-4 | 2.75 (2.53-3.00) | 1.37 (1.23-1.52) | 2.35 (1.84-3.00) |
|  | Weeks 5-28 | 1.40 (1.34-1.47) | 1.08 (0.99-1.17) | 1.29 (1.02-1.62) |
|  | Weeks 29-52 | 1.19 (1.14-1.26) | - | - |
|  | Weeks 53-102 | 94.6 (87.9-102) | 24.4 (21.6-27.5) | 33.2 (24.6-44.9) |
| Irritable bowel syndrome | Day 0 | 46.1 (42.1-50.4) | 13.2 (11.5-15.2) | 36.5 (29.0-46.0) |
|  | Weeks 1-4 | 2.41 (2.23-2.60) | 1.21 (1.09-1.33) | 3.44 (2.92-4.05) |
|  | Weeks 5-28 | 1.11 (1.06-1.15) | 1.09 (1.01-1.17) | 1.36 (1.13-1.64) |
|  | Weeks 29-52 | 1.09 (1.04-1.14) | - | - |
|  | Weeks 53-102 | 1.13 (1.05-1.22) | - | - |

X insufficient events

## Supplementary Table 16: Adjusted hazard ratios (95% CI) comparing the incidence of gastrointestinal disease events after COVID-19 with the incidence before or without COVID-19, in the pre-vaccination, vaccinated and unvaccinated cohorts, in individuals without prior history of gastrointestinal diseases. Hazard ratios are maximally adjusted.

| Subgroup and outcome | Time since COVID-19 | Pre-vaccination cohort | Vaccinated cohort | Unvaccinated cohort |
| --- | --- | --- | --- | --- |
| Upper gastrointestinal bleeding | Day 0 | 243 (222-265) | 72.0 (61.4-84.4) | 183 (133-252) |
|  | Weeks 1-4 | 5.36 (4.78-6.01) | 1.86 (1.51-2.28) | 8.98 (6.63-12.2) |
|  | Weeks 5-28 | 1.74 (1.61-1.88) | 1.30 (1.09-1.55) | 1.92 (1.22-3.00) |
|  | Weeks 29-52 | 1.40 (1.28-1.52) | - | - |
|  | Weeks 53-102 | 1.36 (1.16-1.58) | - | - |
| Lower gastrointestinal bleeding | Day 0 | 116 (105-129) | 34.6 (28.7-41.8) | 102 (71.0-147) |
|  | Weeks 1-4 | 3.00 (2.65-3.40) | 1.40 (1.15-1.70) | 5.53 (3.96-7.71) |
|  | Weeks 5-28 | 1.54 (1.44-1.65) | 1.35 (1.17-1.56) | 1.91 (1.29-2.85) |
|  | Weeks 29-52 | 1.36 (1.26-1.46) | - | - |
|  | Weeks 53-102 | 1.34 (1.17-1.54) | - | - |
| Gastro-oesophageal reflux | Weeks 1-4 | 47.4 (42.0-53.5) | 15.3 (12.7-18.4) | 49.9 (34.2-72.8) |
|  | Weeks 5-28 | 2.82 (2.56-3.12) | 1.24 (1.06-1.45) | 3.92 (2.93-5.26) |
|  | Weeks 29-52 | 1.38 (1.31-1.46) | 1.19 (1.07-1.34) | 2.17 (1.64-2.88) |
|  | Weeks 53-102 | 1.26 (1.19-1.33) | - | - |
|  | Weeks 1-4 | 1.42 (1.28-1.57) | - | - |
| Dyspepsia | Day 0 | 34.2 (28.2-41.5) | 3.83 (2.06-7.13) | 50.6 (31.2-82.1) |
|  | Weeks 1-4 | 1.92 (1.63-2.25) | 1.08 (0.84-1.39) | 3.65 (2.48-5.38) |
|  | Weeks 5-28 | 1.34 (1.24-1.45) | 1.18 (0.99-1.40) | 2.03 (1.39-2.98) |
|  | Weeks 29-52 | 1.31 (1.21-1.42) | - | - |
|  | Weeks 53-102 | 1.23 (1.05-1.44) | - | - |
| Gallstones | Day 0 | 117 (104-133) | 35.3 (28.4-43.9) | 88.1 (55.1-141) |
|  | Weeks 1-4 | 2.77 (2.37-3.23) | 1.45 (1.16-1.82) | 5.61 (3.76-8.36) |
|  | Weeks 5-28 | 1.55 (1.43-1.67) | 1.20 (1.00-1.42) | 3.97 (2.80-5.61) |
|  | Weeks 29-52 | 1.20 (1.10-1.31) | - | - |
|  | Weeks 53-102 | 1.24 (1.06-1.45) | - | - |
| Irritable bowel syndrome | Day 0 | 19.5 (14.0-27.2) | 3.54 (1.59-7.88) | X |
|  | Weeks 1-4 | 1.13 (0.86-1.48) | 0.88 (0.62-1.24) | X |
|  | Weeks 5-28 | 1.34 (1.22-1.48) | 1.09 (0.87-1.37) | X |
|  | Weeks 29-52 | 1.29 (1.17-1.42) | - | - |
|  | Weeks 53-102 | 1.08 (0.86-1.34) | - | - |

X insufficient events

## Supplementary Table 17: Adjusted hazard ratios (95% CI) comparing the incidence of gastrointestinal disease events after COVID-19 with the incidence before or without COVID-19, in the pre-vaccination, vaccinated and unvaccinated cohorts, in individuals with prior history of gastrointestinal operations. Hazard ratios are maximally adjusted.

| Outcome | Time since COVID-19 | Pre-vaccination cohort | Vaccinated cohort | Unvaccinated cohort |
| --- | --- | --- | --- | --- |
| Upper gastrointestinal bleeding | Day 0 | 158 (144-174) | 42.1 (35.9-49.3) | 58.8 (38.8-89.0) |
|  | Weeks 1-4 | 3.10 (2.71-3.54) | 1.74 (1.49-2.05) | 2.54 (1.66-3.90) |
|  | Weeks 5-28 | 1.57 (1.45-1.69) | 1.11 (0.97-1.28) | 0.69 (0.40-1.21) |
|  | Weeks 29-52 | 1.32 (1.21-1.43) | - | - |
|  | Weeks 53-102 | 1.57 (1.38-1.78) | - | - |
| Lower gastrointestinal bleeding | Day 0 | 94.6 (84.8-106) | 26.4 (22.0-31.7) | 42.8 (27.5-66.6) |
|  | Weeks 1-4 | 2.34 (2.04-2.69) | 1.43 (1.21-1.68) | 1.98 (1.28-3.06) |
|  | Weeks 5-28 | 1.43 (1.33-1.53) | 1.10 (0.96-1.25) | 0.87 (0.55-1.37) |
|  | Weeks 29-52 | 1.20 (1.11-1.30) | - | - |
|  | Weeks 53-102 | 1.31 (1.15-1.49) | - | - |
| Gastro-oesophageal reflux | Weeks 1-4 | 83.4 (77.3-90.1) | 19.9 (17.7-22.4) | 53.5 (41.5-69.0) |
|  | Weeks 5-28 | 3.71 (3.45-3.99) | 1.48 (1.35-1.62) | 3.59 (2.90-4.43) |
|  | Weeks 29-52 | 1.31 (1.25-1.37) | 1.14 (1.06-1.23) | 1.64 (1.32-2.03) |
|  | Weeks 53-102 | 1.15 (1.10-1.21) | - | - |
|  | Weeks 1-4 | 1.27 (1.17-1.37) | - | - |
| Dyspepsia | Day 0 | 33.0 (27.4-39.7) | 11.5 (8.82-15.1) | 20.4 (10.9-38.2) |
|  | Weeks 1-4 | 1.58 (1.34-1.88) | 1.11 (0.91-1.35) | 1.07 (0.60-1.89) |
|  | Weeks 5-28 | 1.21 (1.12-1.31) | 1.06 (0.93-1.22) | 1.30 (0.90-1.88) |
|  | Weeks 29-52 | 1.15 (1.06-1.25) | - | - |
|  | Weeks 53-102 | 1.43 (1.25-1.63) | - | - |
| Gallstones | Day 0 | 50.2 (43.1-58.5) | 13.0 (10.4-16.3) | 40.2 (27.6-58.7) |
|  | Weeks 1-4 | 2.96 (2.61-3.35) | 1.28 (1.10-1.49) | 3.74 (2.87-4.88) |
|  | Weeks 5-28 | 1.10 (1.02-1.19) | 0.99 (0.88-1.12) | 1.42 (1.06-1.92) |
|  | Weeks 29-52 | 1.07 (1.00-1.16) | - | - |
|  | Weeks 53-102 | 1.09 (0.96-1.25) | - | - |
| Irritable bowel syndrome | Day 0 | 108 (95.3-121) | 27.5 (22.8-33.1) | 33.9 (19.5-59.0) |
|  | Weeks 1-4 | 2.76 (2.37-3.21) | 1.48 (1.25-1.76) | 2.37 (1.51-3.72) |
|  | Weeks 5-28 | 1.39 (1.28-1.51) | 1.10 (0.96-1.26) | 1.15 (0.73-1.82) |
|  | Weeks 29-52 | 1.20 (1.09-1.32) | - | - |
|  | Weeks 53-102 | 1.55 (1.36-1.78) | - | - |

X insufficient events

## Supplementary Table 18: Adjusted hazard ratios (95% CI) comparing the incidence of gastrointestinal disease events after COVID-19 with the incidence before or without COVID-19, in the pre-vaccination, vaccinated and unvaccinated cohorts, in individuals without prior history of gastrointestinal operations. Hazard ratios are maximally adjusted.

| Outcome | Time since COVID-19 | Pre-vaccination cohort | Vaccinated cohort | Unvaccinated cohort |
| --- | --- | --- | --- | --- |
| Upper gastrointestinal bleeding | Day 0 | 186 (176-197) | 58.4 (52.5-65.1) | 130 (105-160) |
|  | Weeks 1-4 | 4.09 (3.79-4.41) | 1.89 (1.67-2.14) | 5.09 (4.09-6.35) |
|  | Weeks 5-28 | 1.59 (1.51-1.67) | 1.24 (1.11-1.38) | 1.75 (1.35-2.27) |
|  | Weeks 29-52 | 1.27 (1.20-1.34) | - | - |
|  | Weeks 53-102 | 1.37 (1.25-1.50) | - | - |
| Lower gastrointestinal bleeding | Day 0 | 90.5 (84.6-96.9) | 30.5 (27.0-34.4) | 62.9 (48.8-81.0) |
|  | Weeks 1-4 | 2.52 (2.33-2.73) | 1.40 (1.24-1.57) | 3.30 (2.62-4.17) |
|  | Weeks 5-28 | 1.46 (1.40-1.53) | 1.23 (1.13-1.34) | 1.60 (1.27-2.03) |
|  | Weeks 29-52 | 1.25 (1.20-1.31) | - | - |
|  | Weeks 53-102 | 1.36 (1.25-1.47) | - | - |
| Gastro-oesophageal reflux | Weeks 1-4 | 65.9 (62.5-69.5) | 17.8 (16.2-19.6) | 56.5 (47.6-66.9) |
|  | Weeks 5-28 | 3.23 (3.08-3.39) | 1.39 (1.29-1.49) | 3.87 (3.36-4.45) |
|  | Weeks 29-52 | 1.35 (1.32-1.39) | 1.23 (1.17-1.30) | 1.65 (1.42-1.92) |
|  | Weeks 53-102 | 1.22 (1.18-1.25) | - | - |
|  | Weeks 1-4 | 1.34 (1.28-1.42) | - | - |
| Dyspepsia | Day 0 | 26.9 (24.0-30.3) | 6.18 (4.76-8.02) | 33.9 (25.2-45.4) |
|  | Weeks 1-4 | 1.66 (1.51-1.82) | 1.00 (0.87-1.15) | 2.64 (2.11-3.31) |
|  | Weeks 5-28 | 1.26 (1.21-1.32) | 1.15 (1.05-1.27) | 1.65 (1.33-2.03) |
|  | Weeks 29-52 | 1.24 (1.18-1.29) | - | - |
|  | Weeks 53-102 | 1.17 (1.08-1.28) | - | - |
| Gallstones | Day 0 | 95.7 (88.9-103) | 25.8 (22.7-29.3) | 43.0 (32.2-57.4) |
|  | Weeks 1-4 | 2.71 (2.49-2.96) | 1.33 (1.19-1.50) | 2.96 (2.33-3.75) |
|  | Weeks 5-28 | 1.46 (1.40-1.53) | 1.10 (1.00-1.20) | 1.83 (1.47-2.27) |
|  | Weeks 29-52 | 1.21 (1.15-1.27) | - | - |
|  | Weeks 53-102 | 1.28 (1.18-1.39) | - | - |
| Irritable bowel syndrome | Day 0 | 38.8 (35.0-43.1) | 13.2 (11.3-15.5) | 33.7 (25.7-44.2) |
|  | Weeks 1-4 | 1.92 (1.75-2.11) | 1.12 (0.99-1.27) | 2.95 (2.42-3.60) |
|  | Weeks 5-28 | 1.15 (1.10-1.20) | 1.16 (1.06-1.26) | 1.30 (1.04-1.61) |
|  | Weeks 29-52 | 1.13 (1.08-1.18) | - | - |
|  | Weeks 53-102 | 1.15 (1.05-1.25) | - | - |

X insufficient events

## Supplementary Table 19: Estimated number of excess diseases for each outcome per 100,000 COVID-19 diagnoses, in the pre-vaccination, vaccinated and unvaccinated cohorts. Increases in risks were estimated within age groups, and the estimated overall increase in risk is the average of these, weighted according to the proportions in each age group in the pre-vaccination cohort.

| Outcome | Pre-vaccination cohort | Vaccinated cohort | Unvaccinated cohort |
| --- | --- | --- | --- |
| Upper gastrointestinal bleeding | 159 | 60 | 112 |
| Lower gastrointestinal bleeding | 144 | 64 | 91 |
| Gastro-oesophageal reflux | 331 | 165 | 296 |
| Dyspepsia | 66 | 29 | 101 |
| Gallstones | 91 | 30 | 106 |
| Irritable bowel syndrome | 66 | 32 | 107 |

# The RECORD checklist

|  | **Item No.** | **STROBE items** | **Location in manuscript where items are reported** | **RECORD items** | **Location in manuscript where items are reported** |
| --- | --- | --- | --- | --- | --- |
| **Title and abstract** | | | | | |
|  | 1 | (a) Indicate the study’s design with a commonly used term in the title or the abstract (b) Provide in the abstract an informative and balanced summary of what was done and what was found | Title; Abstract (Background, Design, Results, Conclusion). **Pages (1,2)** | RECORD 1.1: The type of data used should be specified in the title or abstract. When possible, the name of the databases used should be included.  RECORD 1.2: If applicable, the geographic region and timeframe within which the study took place should be reported in the title or abstract.  RECORD 1.3: If linkage between databases was conducted for the study, this should be clearly stated in the title or abstract. | 1.1Abstract; Methods *Study design and data sources*; *Data sharing.* **Pages (2,4,5,7)**  1.2 Title (England); Abstract;  **Pages (1,2)**  1.3 Linkage already done by OpenSafely (Methods *Study design and data sources*; *Information governance and ethical approval)* **Pages (4-5, 16-18)** |
| **Introduction** | | | | | |
| Background rationale | 2 | Explain the scientific background and rationale for the investigation being reported | Introduction first three paragraphs. **Pages (3-4)** |  |  |
| Objectives | 3 | State specific objectives, including any prespecified hypotheses | Introduction final paragraph **Pages (4)** |  |  |
| **Methods** | | | | | |
| Study Design | 4 | Present key elements of study design early in the paper | Methods *Study design and data sources*; *Study population.* **Pages (4-5)** |  |  |
| Setting | 5 | Describe the setting, locations, and relevant dates, including periods of recruitment, exposure, follow-up, and data collection | Methods *Study design and data sources*; *Study population* (definition of 3 cohorts and follow-up dates). **Pages (4-6)** |  |  |
| Participants | 6 | *(a) Cohort study* - Give the eligibility criteria, and the sources and methods of selection of participants. Describe methods of follow-up  *Case-control study* - Give the eligibility criteria, and the sources and methods of case ascertainment and control selection. Give the rationale for the choice of cases and controls  *Cross-sectional study* - Give the eligibility criteria, and the sources and methods of selection of participants  *(b) Cohort study* - For matched studies, give matching criteria and number of exposed and unexposed  *Case-control study* - For matched studies, give matching criteria and the number of controls per case | Methods *Study population* (eligibility criteria, inclusion/exclusion, cohort definitions). **Pages (4,5)** | RECORD 6.1: The methods of study population selection (such as codes or algorithms used to identify subjects) should be listed in detail. If this is not possible, an explanation should be provided.  RECORD 6.2: Any validation studies of the codes or algorithms used to select the population should be referenced. If validation was conducted for this study and not published elsewhere, detailed methods and results should be provided.  RECORD 6.3: If the study involved linkage of databases, consider use of a flow diagram or other graphical display to demonstrate the data linkage process, including the number of individuals with linked data at each stage. | 6.1Methods  *Study population.* **Page (5-7)**  6.2 Methods *Study design and data sources.* **Page (4-5)**  No separate validation study conducted for code lists in this manuscript; outcomes and exposure were defined using pre-specified, openly available codelists validated by a clinician developed for this study (URL in Methods)  6.3*Supplementary Figures 3-4*  **Page (Supplements p 27-28)** |
| Variables | 7 | Clearly define all outcomes, exposures, predictors, potential confounders, and effect modifiers. Give diagnostic criteria, if applicable. | Methods Gastrointestinal *diseases studied*; *Date of COVID-19 diagnosis*; *Covariates*; *Study population* (hospitalised vs non-hospitalised COVID-19).  **Pages (5-7) & supplementary Tables 1-2** | RECORD 7.1: A complete list of codes and algorithms used to classify exposures, outcomes, confounders, and effect modifiers should be provided. If these cannot be reported, an explanation should be provided. | Methods *Gastrointestinal diseases studied* (codelists URL); *Covariates*; *Data sharing.*  **Pages(5-7) & supplementary Tables 1-2** |
| Data sources/ measurement | 8 | For each variable of interest, give sources of data and details of methods of assessment (measurement).  Describe comparability of assessment methods if there is more than one group | Methods *Study design and*  *data sources*  *Gastrointestinal diseases*  *studied*; *Date of COVID-19*  *diagnosis*;  *Covariates*.  **Pages (4-6) & supplementary Tables 1-2** |  |  |
| Bias | 9 | Describe any efforts to address potential sources of bias | Methods Statistical analyses (sampling, weighting, adjustment); **Pages (7-8)** |  |  |
| Study size | 10 | Explain how the study size was arrived at | Methods Study population (numbers in each cohort). **Page (5)** |  |  |
| Quantitative variables | 11 | Explain how quantitative variables were handled in the analyses. If applicable, describe which groupings were chosen, and why | Methods Covariates; Statistical analyses (handling of continuous variables, age standardisation…).  **Pages (7-8)** |  |  |
| Statistical methods | 12 | (a) Describe all statistical methods, including those used to control for confounding  (b) Describe any methods used to examine subgroups and interactions  (c) Explain how missing data were addressed  (d) *Cohort study* - If applicable, explain how loss to follow-up was addressed  *Case-control study* - If applicable, explain how matching of cases and controls was addressed  *Cross-sectional study* - If applicable, describe analytical methods taking account of sampling strategy  (e) Describe any sensitivity analyses | Methods Statistical analyses (Cox models, calendar time scale, sampling and weighting, subgroup analyses, excess risk)  **Pages (7-8)** |  |  |
| Data access and cleaning methods |  | .. |  | RECORD 12.1: Authors should describe the extent to which the investigators had access to the database population used to create the study population.  RECORD 12.2: Authors should provide information on the data cleaning methods used in the study. | 12.1 Information governance and ethical approval (access arrangements, contracts with NHS England). **Pages (16-18)**  12.2 Data cleaning Methods Study design and data sources / Statistical analyses; Supplementary Methods (details of sampling, exclusions, quality filters). **Pages (5-8) & Supplementary Page 4 )** |
| Linkage |  | .. |  | RECORD 12.3: State whether the study included person-level, institutional-level, or other data linkage across two or more databases. The methods of linkage and methods of linkage quality evaluation should be provided. | 12.3 Linkage methods & quality Methods Study design and data sources; Information governance and ethical approval (linkage using pseudonymised NHS number, encryption, disclosure control).  **Pages (4-5,16-18)** |
| **Results** | | | | | |
| Participants | 13 | (a) Report the numbers of individuals at each stage of the study (*e.g.*, numbers potentially eligible, examined for eligibility, confirmed eligible, included in the study, completing follow-up, and analysed)  (b) Give reasons for non-participation at each stage.  (c) Consider use of a flow diagram | Results Cohort characteristics (numbers in each cohort, COVID-19 diagnosis counts and percentages); Overall incidence; Supplementary Figure 4 (cohort flow). **Pages (8-9 & supplements p 28)** | RECORD 13.1: Describe in detail the selection of the persons included in the study (*i.e.,* study population selection) including filtering based on data quality, data availability and linkage. The selection of included persons can be described in the text and/or by means of the study flow diagram. | 13.1 Detailed selection / filtering Methods Study population; Results Cohort characteristics; Supplementary Figure 1–2 (study flow)  **Pages (5–7, 8–9) & Supplement p 4, 27–28** |
| Descriptive data | 14 | (a) Give characteristics of study participants (*e.g.*, demographic, clinical, social) and information on exposures and potential confounders  (b) Indicate the number of participants with missing data for each variable of interest  (c) *Cohort study* - summarise follow-up time (*e.g.*, average and total amount) | Results Cohort characteristics; Table 1 (demographic and clinical characteristics by cohort). **Pages (8-9); Tables and figures p 2)** |  |  |
| Outcome data | 15 | *Cohort study* - Report numbers of outcome events or summary measures over time  *Case-control study* - Report numbers in each exposure category, or summary measures of exposure  *Cross-sectional study* - Report numbers of outcome events or summary measures | Results: **Pages (9-11)** |  |  |
| Main results | 16 | (a) Give unadjusted estimates and, if applicable, confounder-adjusted estimates and their precision (e.g., 95% confidence interval). Make clear which confounders were adjusted for and why they were included  (b) Report category boundaries when continuous variables were categorized  (c) If relevant, consider translating estimates of relative risk into absolute risk for a meaningful time period | Abstract Results; Results disease-specific subsections and absolute excess risk;. **Pages (2,9-11)** |  |  |
| Other analyses | 17 | Report other analyses done—e.g., analyses of subgroups and interactions, and sensitivity analyses | Results Subgroup analysis (age, prior GI events, etc.); Absolute excess risk; Supplementary Tables and Figures.  **Pages (10-11 & Supplements p 9–36)** |  |  |
| **Discussion** | | | | | |
| Key results | 18 | Summarise key results with reference to study objectives | Discussion Main findings (first paragraph). **Page (11-12)** |  |  |
| Limitations | 19 | Discuss limitations of the study, taking into account sources of potential bias or imprecision. Discuss both direction and magnitude of any potential bias | Discussion Strengths and limitations. **Pages (13-14)** | RECORD 19.1: Discuss the implications of using data that were not created or collected to answer the specific research question(s). Include discussion of misclassification bias, unmeasured confounding, missing data, and changing eligibility over time, as they pertain to the study being reported. | 19.1 Discussion Strengths and limitations. **Pages(13-14)** |
| Interpretation | 20 | Give a cautious overall interpretation of results considering objectives, limitations, multiplicity of analyses, results from similar studies, and other relevant evidence | Discussion Main findings; Comparison with literature; Clinical implications.  **Pages (11-13)** |  |  |
| Generalisability | 21 | Discuss the generalisability (external validity) of the study results | Discussion Comparison with literature and Strengths and limitations (representativeness of OpenSAFELY-TPP population, applicability to wider UK/other settings). **Pages (12-14)** |  |  |
| **Other Information** | | | | | |
| Funding | 22 | Give the source of funding and the role of the funders for the present study and, if applicable, for the original study on which the present article is based | Funding section. **Pages (15-16)** |  |  |
| Accessibility of protocol, raw data, and programming code |  |  |  | RECORD 22.1: Authors should provide information on how to access any supplemental information such as the study protocol, raw data, or programming code. | **22.1 Data sharing and code on GitHub; statement that raw data cannot be shared); GitHub URL in Gastrointestinal diseases studied and Data sharing. Pages (7,16-18)** |

*Reference: Benchimol EI, Smeeth L, Guttmann A, Harron K, Moher D, Petersen I, Sørensen HT, von Elm E, Langan SM, the RECORD Working Committee. The Reporting of studies Conducted using Observational Routinely-collected health Data (RECORD) Statement. *PLoS Medicine* 2015; in press.

*Checklist is protected under Creative Commons Attribution ([CC BY](http://creativecommons.org/licenses/by/4.0/)) license.
